# Exploring Structure–Activity Relationships and Modes of Action of Laterocidine

**DOI:** 10.1021/acscentsci.4c00776

**Published:** 2024-08-06

**Authors:** Varsha J. Thombare, James D. Swarbrick, Mohammad A. K. Azad, Yan Zhu, Jing Lu, Heidi Y. Yu, Hasini Wickremasinghe, Xiaoji He, Mahimna Bandiatmakur, Rong Li, Phillip J. Bergen, Tony Velkov, Jiping Wang, Kade D. Roberts, Jian Li, Nitin A. Patil

**Affiliations:** Biomedicine Discovery Institute, ^†^Infection Program and Department of Pharmacology and ^‡^Infection Program and Department of Microbiology, Monash University, Melbourne, VIC 3800, Australia

## Abstract

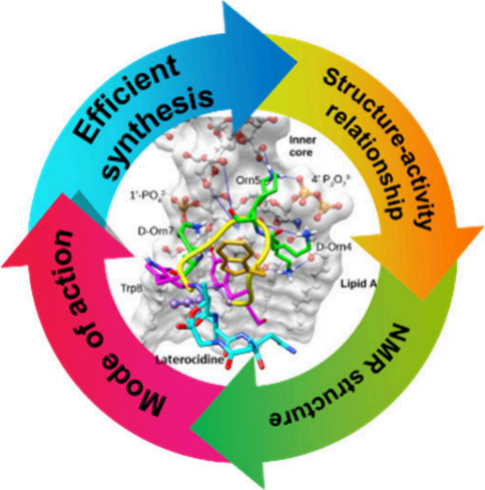

A significant increase in life-threatening infections caused by Gram-negative “superbugs” is a serious threat to global health. With a dearth of new antibiotics in the developmental pipeline, antibiotics with novel mechanisms of action are urgently required to prevent a return to the preantibiotic era. A key strategy to develop novel anti-infective treatments is to discover new natural scaffolds with distinct mechanisms of action. Laterocidine is a unique cyclic lipodepsipeptide with activity against multiple problematic multidrug-resistant Gram-negative pathogens, including *Pseudomonas aeruginosa*, *Acinetobacter baumannii*, and *Enterobacterales*. Here, we developed a total chemical synthesis methodology for laterocidine and undertook systematic structure–activity relationship studies with chemical biology and NMR. We discovered important structural features that drive the antimicrobial activity of laterocidine, leading to the discovery of an engineered peptide surpassing the efficacy of the original peptide. This engineered peptide demonstrated complete inhibition of the growth of a polymyxin-resistant strain of *Pseudomonas aeruginosa* in static time-kill experiments.

## Abstract 2

We report a superior laterocidine analogue with *in vivo* efficacy against highly resistant Gram-negative pathogens, discovered via comprehensive SAR studies and streamlined total chemical synthesis.

## Introduction

Increasing antibiotic resistance which has been compounded by a lack of new anti-infective agents in development foretell a looming preantibiotic apocalypse,^1−3^ predicted to cause 10 million deaths annually by 2050.^4,5^ The World Health Organization (WHO) has identified multidrug-resistant (MDR) Gram-negative bacteria, especially carbapenem-resistant *Pseudomonas aeruginosa*, *Acinetobacter baumannii*, and *Enterobacterales* (e.g., *Klebsiella pneumoniae* and *Enterobacter cloacae*), as the highest priority (critical) pathogens urgently requiring the development of new antibiotic treatments.^5−9^ These Gram-negative “superbugs” present significant medical challenges due to their resistance to almost all antibiotics, including the last-line polymyxins (polymyxin B and colistin).^10,11^ Therefore, novel antibacterial lead scaffolds are urgently needed to tackle the challenge of life-threatening infections by these problematic MDR pathogens.

**Figure 1:**
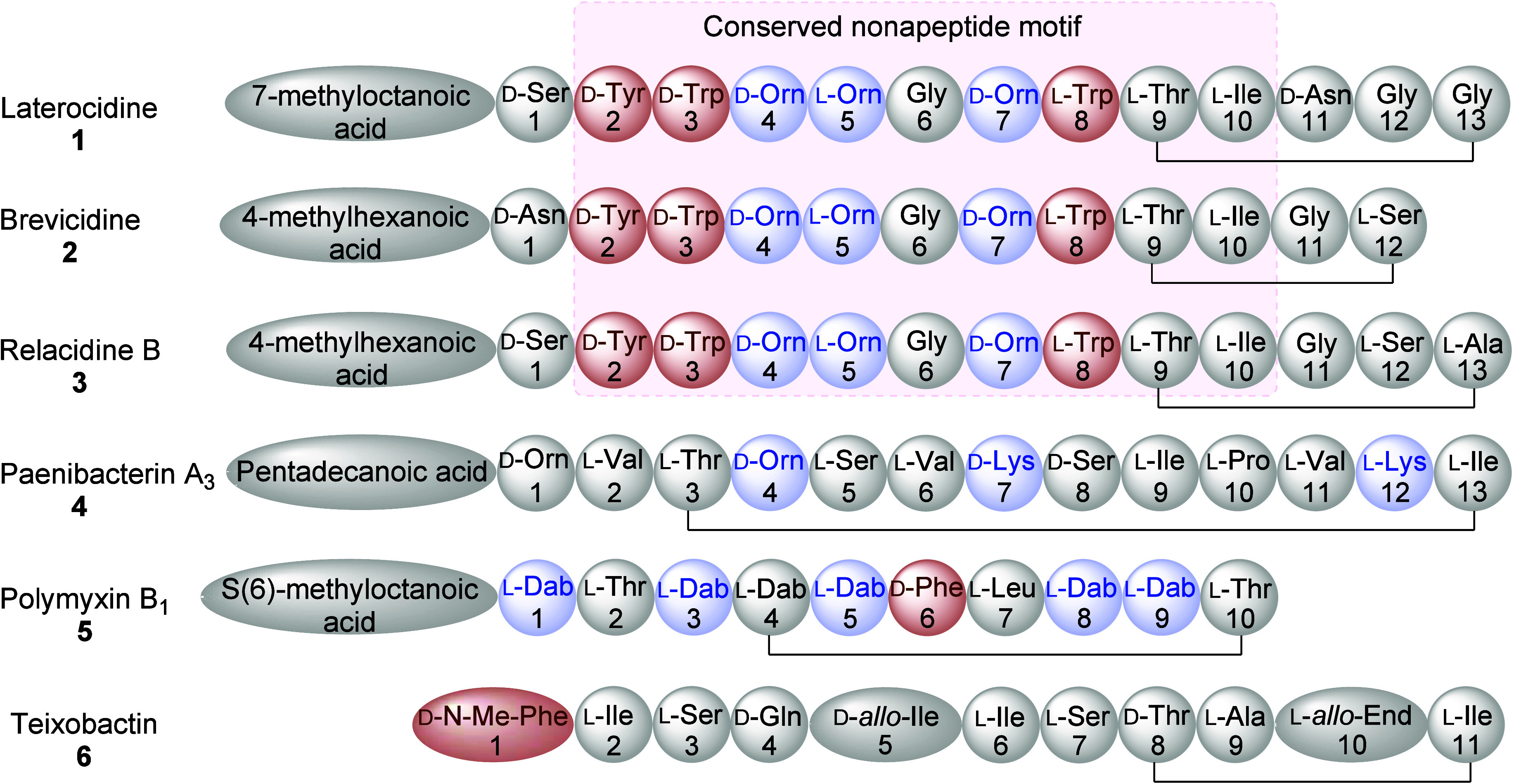
Amino acid sequences of laterocidine (1), brevicidine (2), relacidine (3), paenibacterin A_3_ (4), polymyxin B_1_ (5), and teixobactin (6).

Nonribosomal peptides (NRPs) are structurally diverse families of complex secondary metabolites often displaying antibacterial activities against pathogenic bacteria.^12,13^ Given this activity, NRPs have garnered significant attention in recent years, leading to the identification of several promising antimicrobial peptides including teixobactin,^14^ paenibacterin,^15,16^ relacidine,^17^ brevicidine,^18^ and laterocidine^18−22^ (Figure 1). Notably, brevicidine and laterocidine have shown much promise as potential therapeutics due to their strong activity against MDR Gram-negative bacteria, low *in vitro* toxicity, and low risk of resistance.^20,23^ Significantly, both have also shown activity against polymyxin-resistant Gram-negative bacteria, which adds to their therapeutic potential.^18−21^

Laterocidine-family peptides are cationic, cyclic, and lipo-depsipeptides with a highly conserved motif consisting of 12 (brevicidine) or 13 (laterocidine) amino acids and an *N*-terminal fatty acid moiety (Figure 1).^16,24^ The eight *N*-terminal amino acids form a highly conserved linear peptide segment which is attached to a pentapeptide macrocyclic ring. However, the depsipeptide macrocyclic ring differs significantly in size (penta- or tetrapeptide) between peptides, while the nature of the *C*-terminal residue is also variable (hydrophilic/hydrophobic, flexible/constrained). The carboxylic group of the *C*-terminal residue (Ser12 or Gly13) forms an ester linkage with the hydroxy group of the Thr9 side chain. The key characteristic features of laterocidine-family peptides are the presence of the *N*-terminal fatty acid moiety and a central “nonapeptide” motif, e.g.:
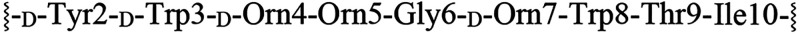
01that always contains three positively charged ornithine residues (_D/L_-Orn), three aromatic residues (_D/L_-Trp and _D_-Tyr), and a central flexible glycine (Gly) residue. Other cyclic depsipeptides (e.g., teixobactin, Figure 1) lack this nonapeptide motif and the *N*-terminal fatty acid moiety. Notably, this signature nonapeptide motif, ester linkage, and relatively smaller ring structure distinguish laterocidine-family peptides from the well-known cationic lipopeptides, which include the polymyxins (Figure 1). These unique structural and biological features make laterocidine-family peptides an attractive scaffold for future antimicrobial drug development. However, the pursuit of systematic structure–function investigations faces a significant hurdle due to the absence of efficient, automated synthetic methodologies. Consequently, research into the structure–activity relationships (SAR) of relacidine, laterocidine, and brevicidine has been sparse.^25−29^ Noteworthy studies have focused on lipid substitution at the *N*-terminal of brevicidine and laterocidine,^29^ thioether bridged cyclic mimetics of relacidine,^23^ and the linearization of brevicidine and laterocidine.^21^

**Scheme 1:**
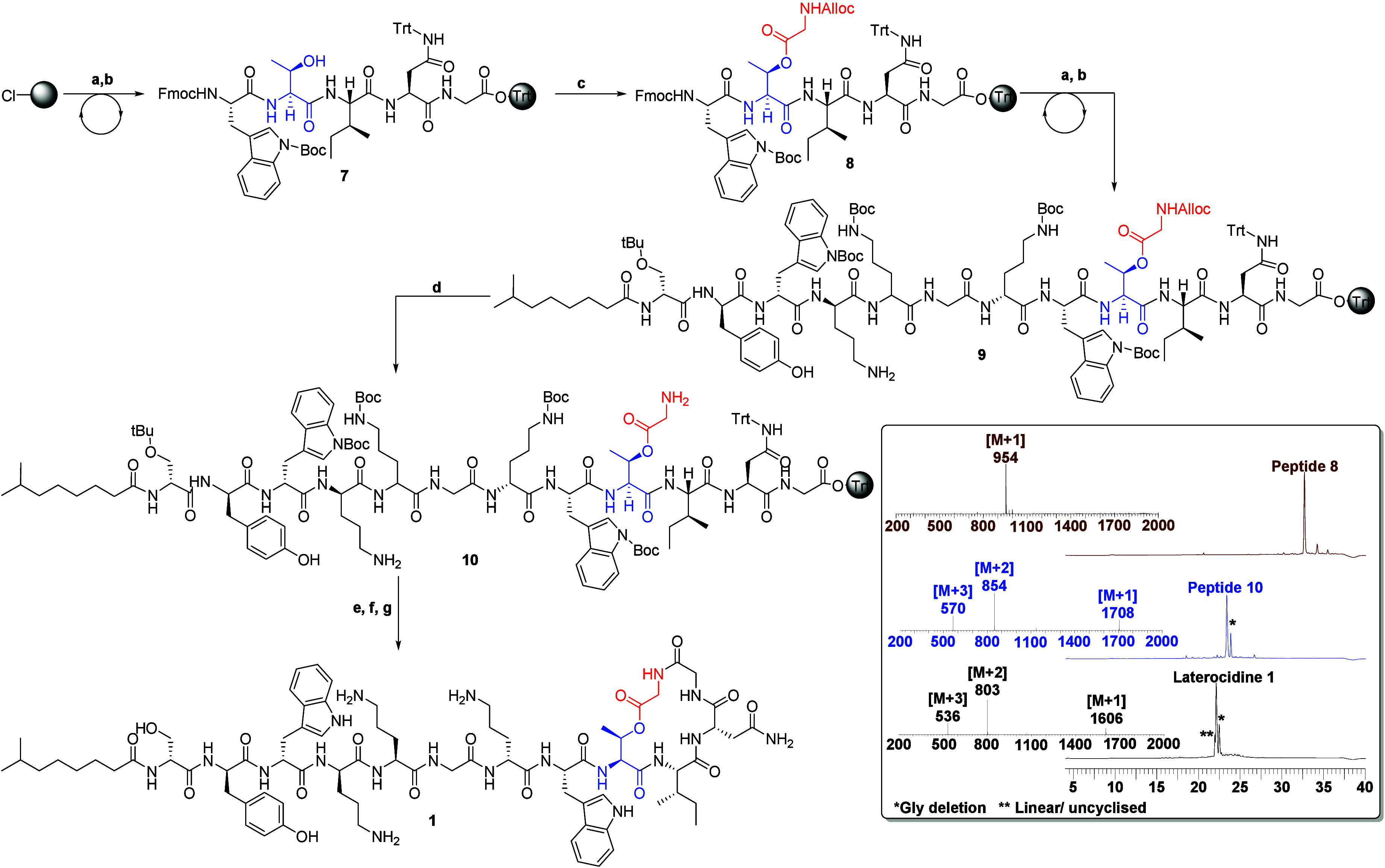
Total Synthesis of Laterocidine (a) Fmoc-AA-OH (3 equiv), HCTU (3 equiv), DIPEA (6 equiv), in DMF 50 min; (b) 20% piperidine/DMF (1 × 5 min, 1 × 10 min); (c) Alloc-Gly-OH (5 equiv), DIC (5 equiv), DMAP (0.3 equiv) in DMF; (d) palladium tetrakis(triphenylphosphine) (0.1 equiv), PhSiH_3_ (10 equiv), in DCM 40 min; (e) 10% HFIP in DCM (1 × 30 min, 1 × 5 min); (f) DPPA, DIEA in DMF 6 h; (g) TFA:TIPS:DODT:H_2_O (92.5:2.5:2.5:2.5) 90 min. HPLC and mass spectrum of peptides **1**, **8**, and **10**. O-(1H-6-chlorobenzotriazole-1-yl)-1,1,3,3-tetramethyluronium hexafluorophosphate (HCTU), 1,1,1,3,3,3-hexafluoro isopropanol (HFIP), dichloromethane (DCM) diphenylphosphoryl azide (DPPA), di-isopropyl carbodiimide (DIC), dimethylaminopyridine (DMAP), diisopropylethylamine (DIEA), dimethylformamide (DMF), triisopropylsilane (TIPS), 3,6-dioxa-1,8-octanedithiol (DODT), and trifluoroacetic acid (TFA).

Motivated by the antimicrobial efficacy and the presence of a 16-membered lactone ring in laterocidine, our focus has been on conducting comprehensive SAR studies through synthetic approaches. To date, the total synthesis of brevicidine and laterocidine was explored by two different solid-phase peptide synthesis (SPPS) strategies.^30,31^ In the previous SPPS protocols, the peptide chain is anchored to the solid support through the Asn side chain. However, this Asn-anchoring hinders the substitution of position 11 and limits the applicability for detailed structure–function studies. Therefore, an efficient and versatile synthetic protocol was developed to facilitate substitution at all positions, including position 11 (Scheme 1).^30,31^ This allows systematic investigation of the mode of action of laterocidine and the role of each amino acid in antimicrobial activity.

Herein, we report detailed structure–activity relationship (SAR) profiling of the cyclic laterocidine scaffold, which is underpinned by our efficient and scalable chemical synthesis strategy. We further investigated its distinct mode of action, revealing the interaction of laterocidine with lipopolysaccharide (LPS), a major component of the outer membrane of Gram-negative bacteria. We further employ solution state NMR to elucidate the structure of laterocidine when bound to lipopolysaccharides (LPS), providing valuable insights into the intermolecular interactions within the complex. Finally, animal bloodstream infection and acute toxicity studies showed excellent *in vivo* activity and safety, which together highlight the therapeutic potential of this novel antibacterial scaffold.

## Results and Discussion

### Synthesis of Laterocidine

Structurally, laterocidine is classified as a cyclic depsipeptide, with the lactone ring formed by the β-hydroxy group of Thr9 and the *C*-terminus of Gly13. The synthesis of laterocidine poses a significant challenge due to the formation of an ester linkage. Currently, three primary strategies are employed for synthesizing laterocidine and brevicidine analogues.^30−32^ In our study, we developed an optimized three-step solid-phase peptide synthesis (SPPS) protocol to synthesize laterocidine and its analogues. Our protocol involved on-resin esterification as the first step followed by peptide chain elongation and macrocyclization through the amide bond between Gly12 and Gly13 (Scheme 1). Initially, the pentapeptide sequence Fmoc-Trp(Boc)-Thr-Ile-Asn(Trt)-Gly-O-chlorotrityl resin (peptide **7**, Scheme 1) was assembled using an automated synthesis approach. The Alloc-Gly-OH was then coupled to peptide **7** to generate the esterified peptide **8** through overnight on-resin esterification using DIC and DMAP. LC–MS analysis confirmed 95–98% conversion for esterified peptide **8** (Scheme 1). Subsequently, the remaining peptide sequence was completed using the standard SPPS protocol, resulting in peptide **9**. Deprotection of the Alloc group on Gly13 was accomplished by treating peptide **9** with Pd(PPh_3_)_4_ and PhSiH_3_, generating resin-bound depsipeptide **10**. This was subjected to a mild acidic condition, specifically a 10% solution of hexafluoroisopropanol in dichloromethane (10% HFIP/DCM), which upon solvent evaporation resulted in crude protected depsipeptide **10**. Subsequently, depsipeptide **10** was subjected to cyclization using diphenylphosphoryl azide (DPPA) and DIEA base in dilute conditions for 6 h. Finally, trifluoroacetic acid (TFA)-mediated global deprotection and reverse-phase high-performance liquid chromatography (RP-HPLC) purification resulted in 27.4 mg (17.1% overall yield) of peptide **1** from a 0.1 mmol scale synthesis.

**Figure 2:**
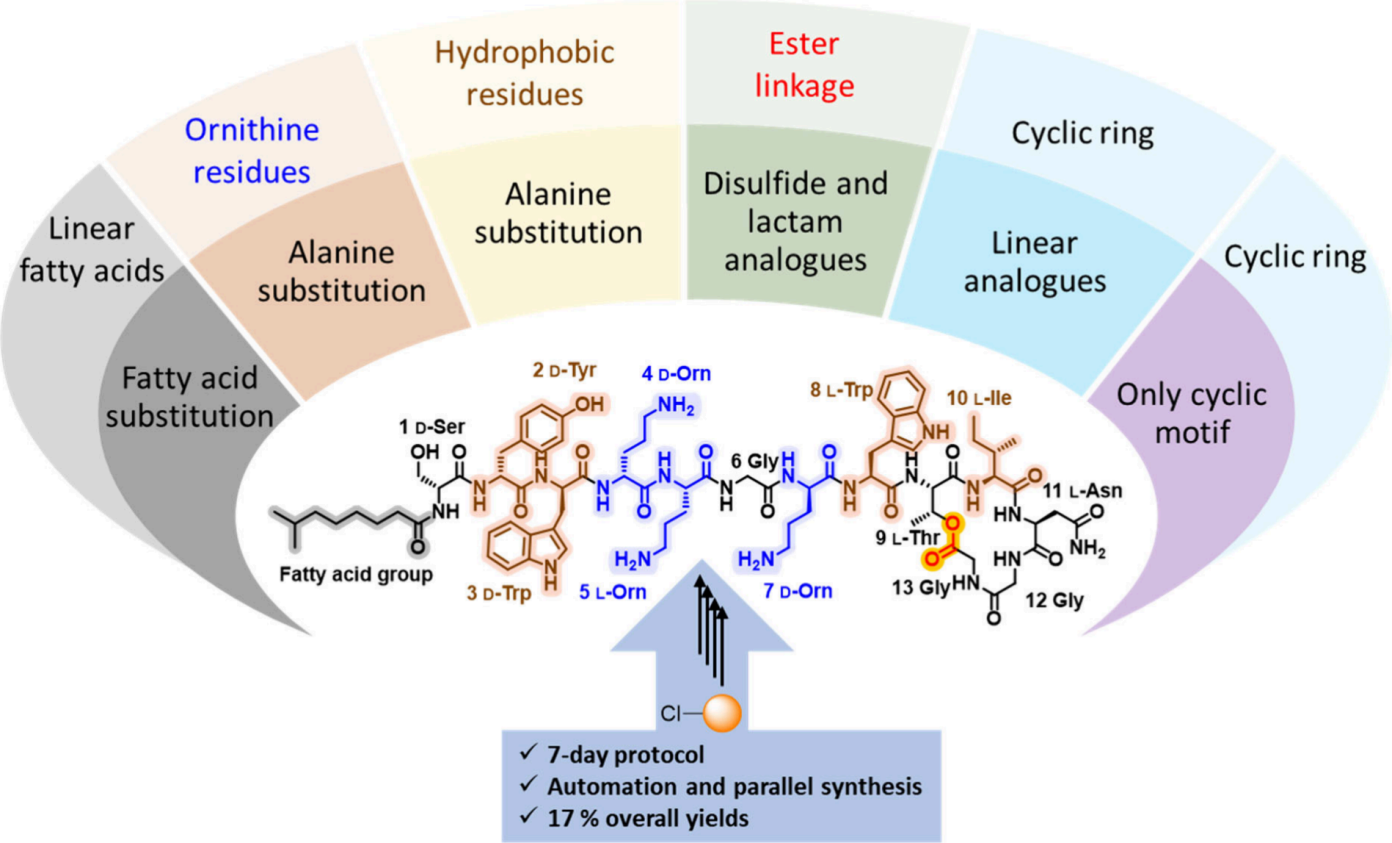
Structure–activity relationship exploration underpinned by solid-phase peptide synthesis protocol. Colored residues are important determinants of antimicrobial activity. Aromatic and hydrophobic residues are highlighted in brown, cationic residues are shown in blue, and ester bond in red.

The efficiency of this protocol is substantiated by its capability to facilitate the seamless execution of all subsequent post-SPPS steps (steps d, e, f, and g in Scheme 1) in a sequential manner, obviating the necessity for intermediate purification procedures. Consequently, this methodology enables the attainment of the final purified analogue within a span of 7 days. Of notable significance is the complete automatability of this method, wherein multiple analogues can be synthesized using a parallel synthesis strategy. This protocol not only enables the synthesis of a substantial quantity of analogues but also we have generated peptide **1** at a gram-scale (Figure S37). This protocol holds significant importance in the realm of systematic SAR investigations, as it allows for the elucidation of the individual residue contributions within the laterocidine scaffold pertaining to its antimicrobial activity (Figure 2).

### *In Vitro* Antimicrobial Studies

The antibacterial activity of the synthesized native laterocidine (peptide **1**) and analogues was assessed against a panel of 18 Gram-negative strains of *P. aeruginosa*, *A. baumannii*, *K. pneumoniae*, and *E. cloacae* (Tables 1 and 2). Six strains (2 each of *P. aeruginosa*, *A. baumannii*, and *K. pneumoniae*) were MDR. The minimum inhibitory concentrations (MIC, μg/mL) of laterocidine **1** ranged from 4–8 μg/mL against *P. aeruginosa*, 2–4 μg/mL against *A. baumannii*, 1–4 μg/mL against *K. pneumoniae*, and 1–2 μg/mL against *E. cloacae*. Interestingly, laterocidine displayed similar antibacterial activity (MICs 1–16 μg/mL) against the MDR strains (Table 1).

**Table 1:**
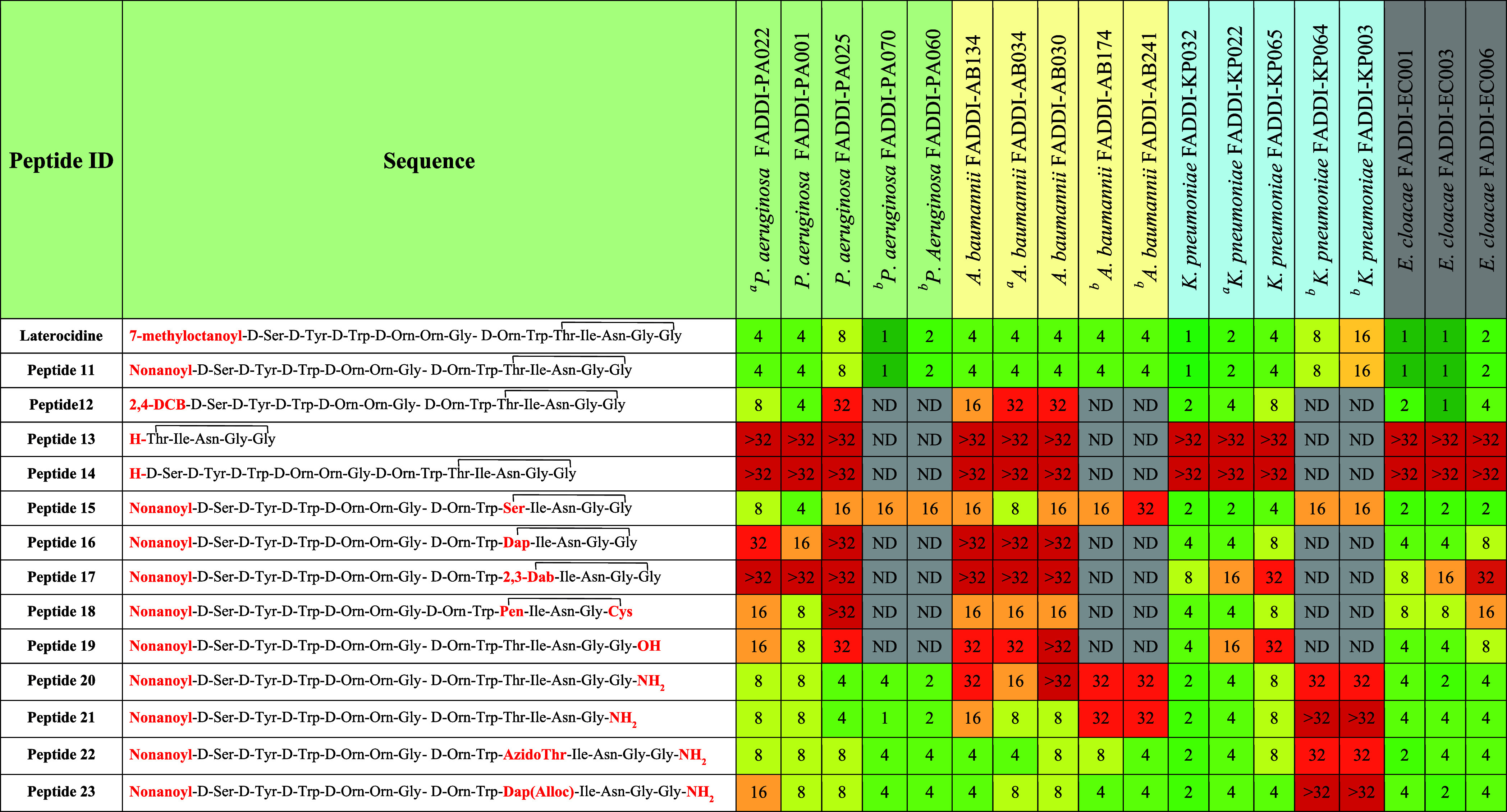
Minimum Inhibitory Concentrations (μg/mL) of Native Laterocidine and Analogues with *N*-Terminal, Linear, and Ring Modifications

a Notes: Carbapenem-resistant isolates.

b Polymyxin-resistant isolates.

**Table 2:**
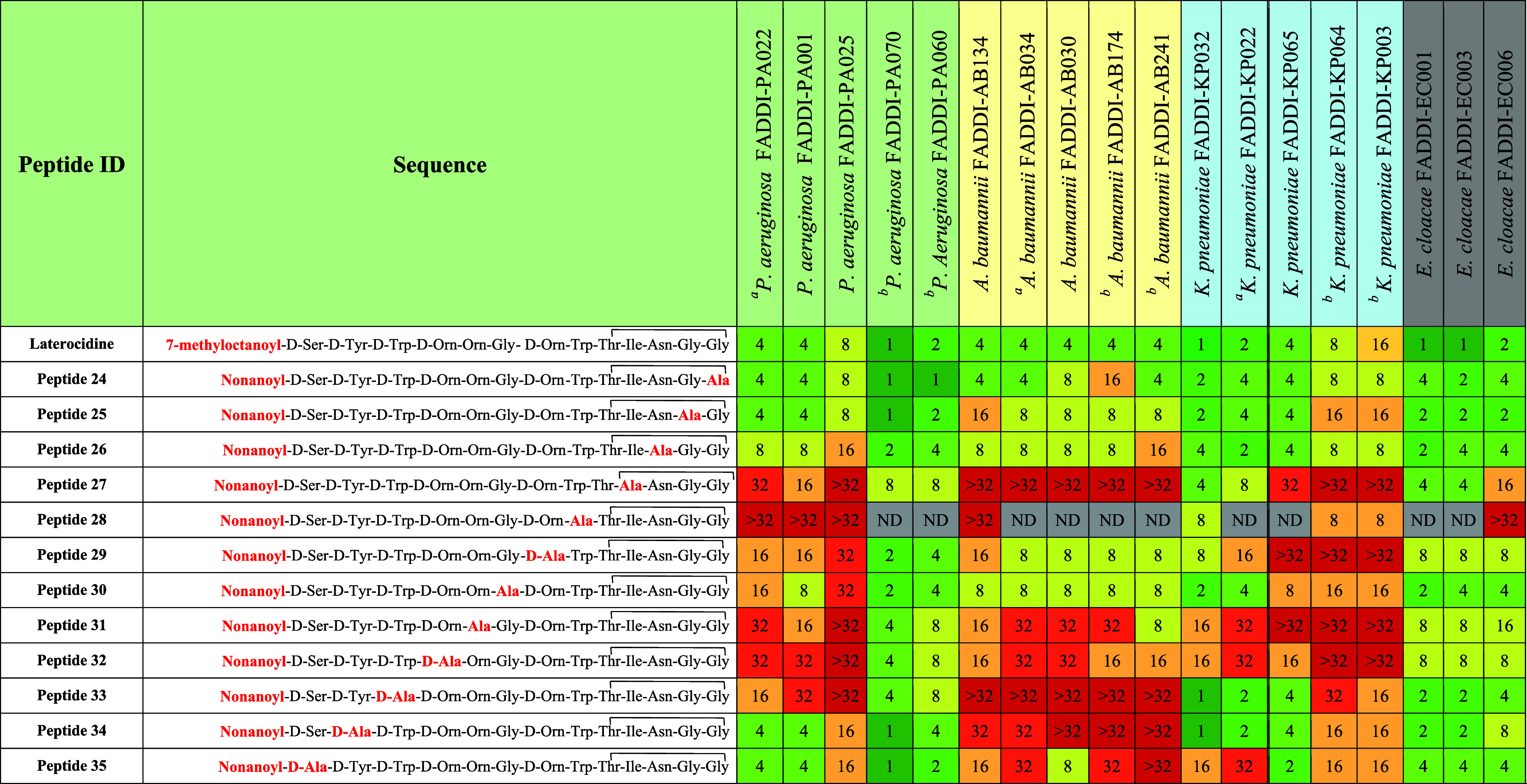
Minimum Inhibitory Concentrations (μg/mL) of Native Laterocidine, Alanine-Substitution Analogues

a Notes: Carbapenem-resistant isolates.

b Polymyxin-resistant isolates.

Our first step in interrogating the SAR of the laterocidine scaffold was to substitute the *N*-terminal 7-methyloctanoyl group with a nonanoyl moiety (peptide **11**). Laterocidine *N*-terminal lipid chain is crucial for the insertion into bacterial membranes. To understand, modifications to the lipid chain of laterocidine affect the antibiotic’s potency. We have selected the same carbon number lipid chain which maintains the balance of hydrophobicity and mass of the native *N*-terminal lipid chain. Peptide **11** maintained antimicrobial activity against all strains, with identical MICs to laterocidine (Table 1). While replacing the 7-methyloctanoyl group with a 2,4-dichlorobenzoyl moiety (peptide **12**) had little impact on the MIC of most isolates, it did result in 4–8-fold increases in MICs against *A. baumannii* (MICs, 8–32 μg/mL). Both cyclic pentapeptide **13** (Figure S2) and the peptide lacking the *N*-terminal fatty acid **14** exhibited significant reductions in antibacterial activity against all tested strains (MICs, >32 μg/mL). These results suggest that the linear octapeptide sequence (amino acids 1 to 8) and *N*-terminal fatty acid play a crucial role in the antibacterial activity of the laterocidine peptide. However, the nonanoyl *N*-terminus peptide **11** showed similar activity to native laterocidine. Therefore, further SAR studies were conducted in a cost-effective way using all laterocidine analogues possessing an *N*-terminal nonanoyl moiety.

As a second step in our systematic investigation, we elucidated the impact of the ester linkage and macrocyclic ring motif on antimicrobial activity. Substituting a secondary ester (i.e., Thr9) with a relatively more flexible primary ester linkage (Ser9)^33^ produced peptide **15** which retained antimicrobial activity against *P. aeruginosa*, *K. pneumoniae*, and *E. cloacae*. However, a 2–8-fold loss in the activity was observed against all strains of *A. baumannii* (MICs, 8–32 μg/mL), while 8–16-fold less activity was observed against the two MDR strains of *P. aeruginosa*. Next, we explored the effects of replacing the ester linkage with either an amide (peptides **16** and **17**, Figure S3) or disulfide (peptide **18**) linkage. For both amide analogues, antimicrobial activity against Gram negative strains was substantially reduced across all species (2–>8-fold less activity) but especially for *P. aeruginosa* and *A. baumannii* where virtually all strains had an MIC of >32 μg/mL. In peptide **18**, the ester linkage was replaced with a disulfide linkage by substituting threonine with penicillamine (Pen) and glycine with cysteine (Cys). This disulfide substitution resulted in 2–8-fold increases in MIC against all tested Gram-negative strains (MICs, 2–32 μg/mL). We then investigated linear analogues with a *C*-terminal acid or amide (peptides **19**–**23**) to explore the role of the ester linkage in antibiotic activity.^20^ With peptide **19**, MICs for the Gram negative strains increased 2–8-fold for *P. aeruginosa* and *A. baumannii* (MICs, 8–32 μg/mL) and 2–4-fold for *K. pneumoniae* and *E. cloacae* (MICs, 4–32 μg/mL). Other linear analogues with a *C*-terminal amide peptide **20** displayed 2–4-fold increases in MIC against *P. aeruginosa*, *K. pneumoniae*, and *E. cloacae* (MICs, 4–32 μg/mL). However, peptide **21** with one less Gly amino acid displayed the 1–2-fold increases in MIC against *P. aeruginosa*, *K. pneumoniae*, and *E. cloacae* (MICs, 2–32 μg/mL). Peptide **22** and **23** substitution Thr9 with azidoThr and Dap(Alloc), respectively, showed equivalent increases in MIC against *P. aeruginosa*, *K. pneumoniae*, and *E. cloacae*. However, peptides **22** and **23** showed 2–>8-fold increases in MIC against *A. baumannii* (MICs, 16–>32 μg/mL). This latter result aligns with recent studies in which linearization retains antibacterial activity against some strains.^21^ Collectively, our results emphasize the important role of the macrocyclic ring and ester linkage for antimicrobial activity against *P. aeruginosa* and *A. baumannii*. While linear analogues **20**–**23** provide a valuable template for future drug development investigations, they nevertheless exhibited a notable loss in antimicrobial activity against all four bacterial species compared to the native laterocidine (2–4-fold increases in MIC). Consequently, we employed the native scaffold for SAR and mechanistic studies.

To gain greater insight into the functional contributions of individual amino acids toward the antimicrobial activity of laterocidine, we synthesized a library of Ala-substituted analogues (Table 2, peptides **24**–**35**) at each position of the laterocidine and compared their MICs with native laterocidine. When Gly13 was replaced with Ala (peptide **24**), MICs for all strains were unaffected or only minimally affected (no more than 1-fold increase, except for two *E. cloacae* strains with 2-fold increase). This indicates that modification at position 13 can be tolerated. Similarly, when alanine replaced Gly12 (Ala12; peptide **25**) and Asn11 (Ala11; peptide **26**), any increase in MIC was typically no more than 1-fold and restricted primarily to *P. aeruginosa* and *A. baumannii* (MICs, 2–16 μg/mL). These marginal increases in MIC for all four bacterial species suggest analogues peptide **25** and peptide **26** retained antibacterial activity and that laterocidine sequence Gly11, Gly12, and Asn13 can tolerate alanine mutations.

For all strains, substituting Ile10 with Ala (peptide **27**) resulted in a substantial loss of activity (4 to >8-fold increases in MIC; range, 8 to >32 μg/mL), with an even greater loss observed when Trp8 was replaced with Ala8 (peptide **28**). Activity against *P. aeruginosa* (MICs, 4 to >32 μg/mL) and *A. baumannii* (MICs, >32 μg/mL) was also substantially reduced with laterocidine _D_-Ala3 (peptide **33**). _D_-Ala substitution of _D_-Tyr2 (peptide **34**) and _D_-Ser1 (peptide **35**) reduced activity against *A. baumannii* (MICs typically ≥32 μg/mL) and, for peptide **35**, also against *K. pneumoniae* (MICs, 2–32 μg/mL). These results indicate that an aromatic side chain is essential for antimicrobial activity against all tested species. Additionally, the Trp3, _D_-Trp8, and _D_-Tyr2 residues are crucial for antibacterial activity of the laterocidine peptide against *A. baumannii*.

Ornithine is the only positively charged residue in laterocidine under physiological conditions. Substitution of _D_-Orn7 with _D_-Ala (peptide **29**) produced a 2–4-fold loss of activity against all strains (MICs, 2 to >32 μg/mL), while substitution at Orn5 (peptide **31**) and _D_-Orn4 (peptide **32**) produced 2–8-fold losses (MICs, 4 to ≥32 μg/mL). Clearly, the positive charge on ornithine residues is important for activity.

Our Ala-scan approach revealed that substitutions at positions 3–5, 7–8, and 10 are not well tolerated across all strains, leading to a 4–8-fold reduction in activity. However, alanine substitution within the macrocycle at positions 11–13 is well tolerated. Generally, the presence of positively charged amino acids (i.e., _D_-Orn4, Orn5, and _D_-Orn7) and aromatic residues (Tyr2, _D_-Trp3, and Trp9) are crucial for laterocidine antimicrobial activity. The observed decline in activity can be attributed to significant differences in structural and physicochemical properties between ornithine, tyrosine, and tryptophan, with _D_-Ala substitution at positions 2–5, 7, and 9 significantly altering the overall hydrophobicity and charge of the resulting peptide analogues.

Alanine substitution at Gly6, Gly12, Gly13, and Asn11 produced no or minimal (≤1-fold) reductions in activity against all four bacterial species, indicating these positions could potentially be explored to optimize the overall physicochemical and secondary structural properties. Additionally, Ala substitution at _D_-Ser1 and _D_-Tyr2 produced ≤2-fold reductions in activity against *P. aeruginosa*, *K. pneumoniae*, and *E. cloacae*, indicating these positions could also be explored in position-specific SAR studies.

### Elucidating the Mode of Action of Laterocidine

To gain insight into the mode of action of laterocidine, we conducted time-kill kinetic experiments and assessed its impact on bacterial membrane integrity using FACS.

**Figure 3:**
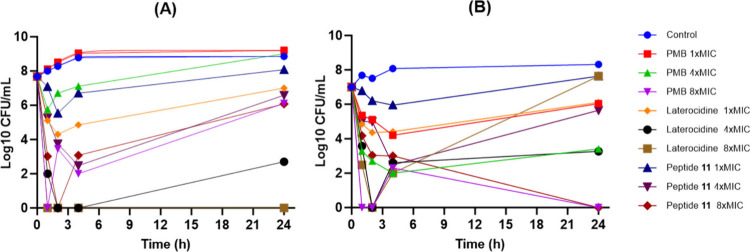
Time-kill studies with polymyxin B (PMB), laterocidine (peptide **1)**, and peptide **11** against (A) polymyxin-susceptible *P. aeruginosa* PAO1 and (B) polymyxin-resistant *P. aeruginosa* PAO1R. All peptides were used at 1, 4, and 8× MIC.

Antimicrobial kinetics were evaluated using polymyxin B, laterocidine (peptide **1**), and peptide **11** against a polymyxin-susceptible reference strain of *P. aeruginosa* PAO1 (polymyxin B MIC, 0.5 μg/mL) and its paired polymyxin-resistant strain *P. aeruginosa* PAO1R (polymyxin B MIC, 16 μg/mL) (Figure 3). The laterocidine and peptide **11** MICs for both strains were 8 and 2 μg/mL, respectively (Table S1). Against the polymyxin-susceptible strain, no (1 × MIC) or minimal (4 × MIC) bacterial killing was observed with the two lowest concentrations of polymyxin B. While substantial initial bacterial killing was observed at 8 × MIC such that no viable bacteria were detected at 1 h, rapid and significant regrowth subsequently occurred. Substantially greater bacterial killing was observed with equivalent polymyxin B concentrations against the polymyxin-resistant strain, with no viable bacteria detected at 8 × MIC on multiple occasions, including at 24 h. While greater initial bacterial killing was achieved against *P. aeruginosa* PAO1 with peptide **11** at 1 × and 4 × MIC, substantial bacterial regrowth nevertheless occurred at all concentrations. Against *P. aeruginosa* PAO1R, killing was slightly less at the lower concentrations than with polymyxin B; however, no viable bacteria were detected at 24 h at 8 × MIC. For laterocidine, bacterial killing of *P. aeruginosa* PAO1 was significantly enhanced, particularly at the higher concentrations. Notably, bacterial eradication was achieved at 24 h with 8 × MIC, while regrowth with 4 × MIC at this time was only ∼2 log 10 CFU/mL. Overall, the time-kill kinetics suggests that native laterocidine was less effective against *P. aeruginosa* PAO1R, with substantial regrowth occurring at all concentrations despite initial killing.

**Figure 4:**
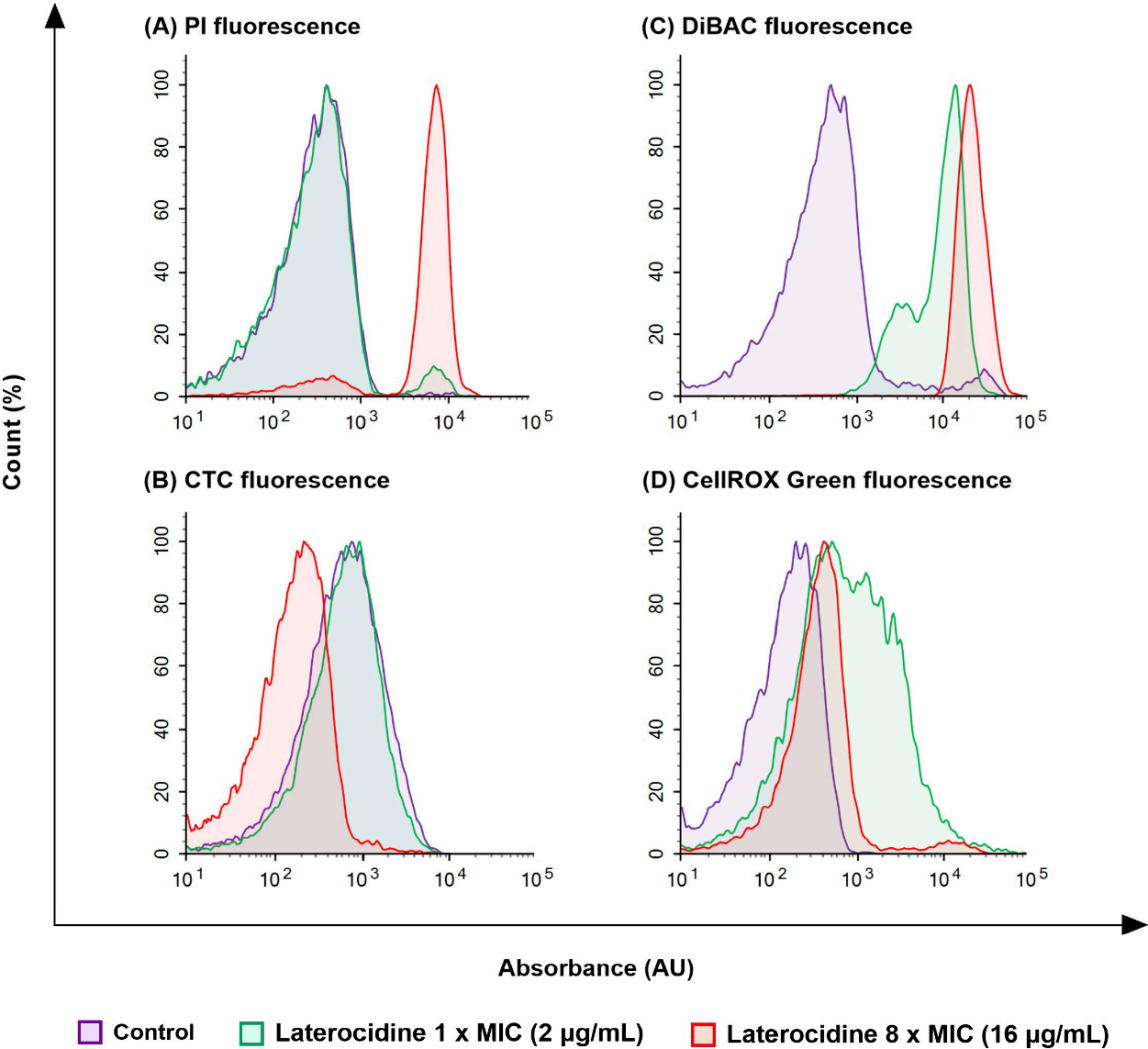
Flow cytometry analysis of *P. aeruginosa* PAO1 with no treatment (control) or following 1 h of treatment with laterocidine (peptide **1**) at 1 × or 8 × MIC (equivalent to 2 and 16 μg/mL, respectively). (A) Membrane integrity (PI), (B) respiration (CTC), (C) membrane polarity (DiBAC), and (D) oxidative stress (CellROX Green). PI, propidium iodide; CTC, 5-cyano-2,3-ditolyl tetrazolium chloride; and DiBAC, bis(1,3-dibutylbarbituric acid) trimethine oxonol.

Disruption by laterocidine to bacterial cells in the time-kill studies was visualized using flow cytometry by staining *P. aeruginosa* PAO1 cells exposed for 1 h to 1 × MIC (2 μg/mL) or 8 × MIC (16 μg/mL) laterocidine with four fluorescent nucleic acid dyes (propidium iodide (PI), 5-cyano-2,3-ditetrazolium chloride (CTC), DiBAC, and CellROX Green). A concentration-dependent disruption of membrane integrity was observed with both PI and DiBAC fluorescence. Consistent with the laterocidine MICs, no loss of membrane permeability as assessed by propidium iodide fluorescence was observed at 1 × MIC (Figure 4A, green curve). However, membrane integrity was severely affected at 8 × MIC, with a substantial increase in permeability observed (Figure 4A, red curve). Disruption of membrane potential as determined by an increase in DiBAC fluorescence showed significant staining at both 1 × and 8 × MIC suggesting extensive loss of membrane potential at each concentration, with slightly greater disruption at 8 × MIC (Figure 4C). Metabolic activity as determined by CTC fluorescence was unaffected at 1 × MIC but markedly reduced at 8 × MIC (Figure 4B), while intracellular oxidative stress visualized using CellROX Green was shown to increase at both 1 × and 8 × MIC (Figure 4D). Furthermore, CR-G fluorescence was observed to increase with laterocidine treatment, with the most effect seen with 8 × MIC (Figure 4D).

These results indicate that while laterocidine interacts with the bacterial membrane, it may have additional modes of action which is independent of direct lysis of the outer membrane. For example, the cell permeable fluorogenic probe CellROX Green acts independently of membrane disruption. The positive staining with CellROX green (Figure 4D) indicates the generation of free radicals due to the bacterial oxidative stress response. To further investigate whether membrane disruption is required for activity, we determined the laterocidine MICs of *A. baumannii* 5075 (wild type), *A. baumannii* 5075D, and *A. baumannii* 5075R. *A. baumannii* 5075D lacks lipid A in the outer membrane while 5075R contains lipid A modified with phosphoethanolamine.^34^ The significant loss in activity of laterocidine against both 5075D and 5075R (MICs, 32 μg/mL; Table S2) indicates interaction with the lipid A within the bacterial outer membrane is required for overall activity. Collectively, our MIC and FACS results align with previous studies, indicating that a critical site of interaction for laterocidine is the lipid A within the bacterial outer membrane.^35^

These preliminary mechanistic studies indicate that the mode of action of laterocidine is complex and may depend on the dynamics of peptide accumulation and localization during bacterial growth.

## Interactions of Laterocidine with Lipopolysaccharide

To examine structural interactions between native laterocidine and bacterial lipid membranes, we performed NMR spectroscopy studies using NMR buffers containing and lacking *E. coli* LPS. The ^1^H and ^1^H-attached ^13^C resonances of laterocidine (peptide **1**) were assigned using 2D homonuclear and heteronuclear NMR data (see the Materials and Methods section) in an acetate buffer pH 4.5 at 23 °C. The side chain NH_2_ peaks of Asn11 were not observed in the free peptide spectra and presumed broadened due to exchange effects. At room temperature (23 °C), the free peptide appeared to be predominantly unstructured as judged by the observation of mainly intraresidue and sequential ^1^H–^1^H NOEs (nuclear Overhouser effects), a paucity of medium range NOEs in the 2D NOESY experiment (Figure S34), and quite unremarkable values of the ^3^J_HNHA_ coupling constant (6–7 Hz). Some residual structure was apparent by the observation of very weak, medium range NOEs between the Hα proton of Ile10 and the amide HN proton of Gly12 and between Ile10 HN and Gly13 HN as well as NOEs between the side chain aromatics of Trp8 and the methyl groups of Ile10. A reduced amide temperature coefficient (Table S5) for Gly13 (−0.9 ppb/K) may be suggestive of a solvent protected amide and likely hydrogen bond.

We next investigated the structure of laterocidine in the presence of LPS. LPS forms very large (*R*g ∼ 100 nM),^36^ heterogeneous micellular structures (with a critical micelle concentration of 1.3–1.6 μM) and has been used to probe the LPS-bound conformation of various antimicrobial peptides.^37^ Typically, cationic peptides bind with weak-to-moderate affinity (*K*_D_ in the micro- to millimolar range) to LPS micelles with fast kinetics that is amenable for transferred NOE-based methods to interrogate the LPS-bound form.

Titration of Gram-negative bacteria-derived LPS into solution of laterocidine at 23 °C induced line broadening in the ^1^H NMR spectra (Figures 4 and S33) with some very minor chemical shift changes, indicating weak to moderate binding affinity and fast-intermediate exchange on the chemical shift time scale. Compared to the same sample without added LPS, a notable increase in the number and intensity of NOEs was observed in the 2D NOESY experiment (Figure S34). These transferred NOEs (trNOEs) originate from short ^1^H–^1^H distances within the transiently LPS-bound structure, which are “transferred” to and recorded over the free-state signals of the peptide. Notably, the same trNOEs were insensitive to the addition of NaCl (100 mM), and samples also appeared stable and clear with unchanged spectra after more than 2 weeks at room temperature.

Overlap of various aliphatic side chain proton chemical shifts (e.g., Ile10 side chain Hγ_1_γ_2_ protons with the lipid Hβ, Hγ/Hδ protons and also the lipid Hε with the Thr10 methyl protons) hindered the assignment of potential long-range aliphatic side chain to aromatic side chain NOEs and hampered structure calculations. Cooling the sample (e.g., to 12 °C) was tested but proved ineffective as it resulted in a reduced transferred NOE sensitivity which we attribute to slower binding kinetics (or peptide/micelle aggregation); this was reversible after warming the sample back to 23 °C. Nevertheless, unambiguous, medium-range trNOEs were observed (Figure S35) between the lipid methyl group and _D_-Tyr2 and _D_-Trp3 aromatic rings, revealing a hydrophobic “triad” motif. Several trNOEs were also observed showing that the side chains of Ile10 and Trp8 were proximal. Finally, long-range trNOEs were observed between the side chains of _D_-Tyr2 and the methyl groups of Ile10 and between the two tryptophan aromatic side chains and lipid methyl groups. This indicates that these two hydrophobic motifs are spatially close across the peptide. No unambiguous long-range backbone NOEs characteristic of a regular beta hairpin-like structure or a contiguous, medium-range set of NOEs characteristic of a regular helix could be resolved.

The trNOEs (Figure S34) were assigned and structures calculated simultaneously within CYANA3^38^ using standard automated approaches with some manual NOE assignments and subsequently refined in XplorNIH.^39,40^ The final structure ensemble was based entirely on 376 assigned trNOEs (Table S3) as no relevant angle restraints can be derived from coupling constants (or chemical shifts) necessarily measured in the free state spectra. The final structure ensemble converged with a backbone and heavy atom RMSD of 0.14 and 0.47 Å, respectively, and low maximum residual restraint violation (see the Supporting Information).

**Figure 5:**
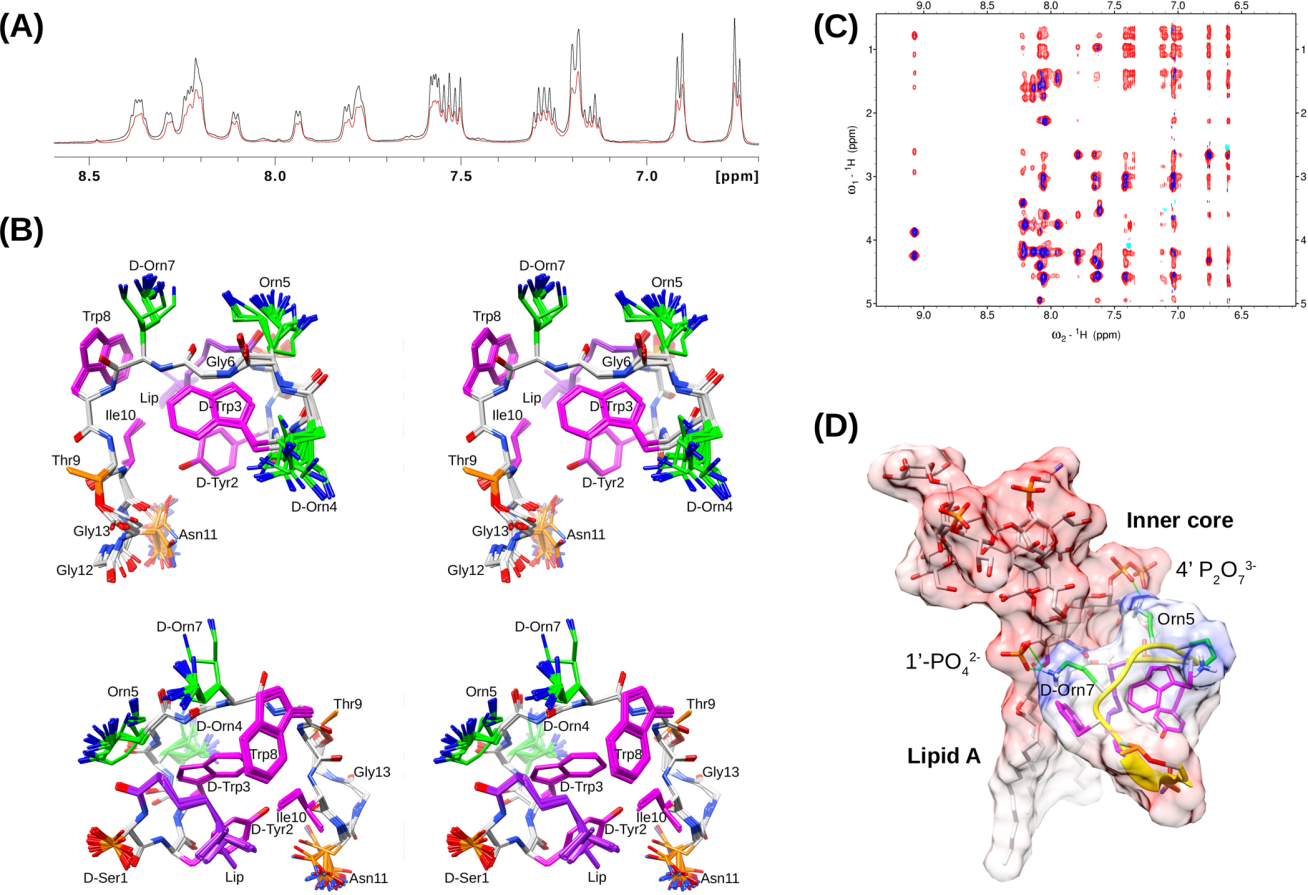
(A) Part of the amide and aromatic (6.7–8.6 ppm) region of the 600 MHz ^1^H NMR spectrum during titration of 1.5 mM laterocidine (black) with 16 μL of 10 mg/mL *E. coli* O111:B4 LPS (red) showing line broadening due to binding to the LPS micelle. (B) Superposed, stereo view of the 41 XplorNIH trNOE NMR structures calculated from a total of 100 as viewed from the top and side. Positively charged ornithine side chains are shown in green, hydrophobic side chains in magenta, and the backbone in gray. Thr, Asn, and _D_-Ser side chains are shown in orange. (C) Superposition of a portion of the 2D NOESY spectra of 1.5 mM laterocidine (blue) and after the addition of *E. coli* LPS0111:B4 (red) both recorded at 23 °C in 50 mM acetate buffer (d_3_), 10% D_2_O, pH 4.5. The 2D NOESY mixing times were 140 and 350 ms for with and without LPS added. (D) Schematic showing a proposed interaction with LPS highlighting the amphipathicity of the laterocidine and complementary separation of the positively charged Orn5 and Orn7 side chains with the sugar phosphates of lipid A in LPS. The backbone of laterocidine is represented as a yellow ribbon. The LPS coordinates was extracted from PDB 6S8H.

The NMR ensemble of laterocidine (Figure 5) depicts a compact structure in which the lipid, _D_-Tyr2, and _D_-Trp3 hydrophobic triad wraps back under a rather extended backbone comprising residues 6–9 which pack against the Ile10/Trp side chain pair forming a hydrophobic core. The hydrophilic Orn residues at positions 4, 5, and 7 protrude outward, generating a positively charged region that is seemingly situated at the top of peptide. The distance between the side chains of Orn4 and Orn7 measures 11.5 Å, which exhibits a modest complementarity to the spacing between the phosphate oxygens within the lipid A component of LPS, specifically measuring 12.5 Å. A hydrogen bond was observed between the amide of Gly13 and carbonyl of Ile10, consistent with the low amide temperature coefficient observed for Gly13 (*vide supra*) in the free peptide. The side chain NH_2_ peaks of Asn12 were not observed in the free peptide spectra and presumed broadened due to exchange effects.

It is noted that the NMR structure was calculated as a single model solution to the trNOE data (which is averaged over all possible peptide binding conformations). Given the uncertainty in the heterogeneity of a large LPS micelle and the likelihood of rapid spin diffusion effects, combined with the relatively unstructured nature of the free peptide as well as plasticity of LPS, we do not rule out the possibility that related structures could be part of the LPS-bound conformational ensemble. Preliminary investigations using the smaller and deuterated DPC micelles to simplify the system gave rise to broad lines and poor quality NOESY spectra consistent with an aggregated peptide which also led to sample precipitation over time (data not shown).

On the basis of the trNOE structure, one plausible simple 1:1 LPS-peptide binding scenario can be envisaged, wherein the side chains of Orn5 and _D_-Orn7 interact with the phosphates of lipid A (or other polar groups) with the *N*-terminal lipid and the aromatic core situated beneath nestled within the lipid-rich environment of the lipopolysaccharide (Figure 5). Curiously, the macrocycle is ostensibly placed on the opposite face, distal to the inferred “hot spot” face of LPS interaction. Within the macrocycle’s architecture, Ile10 stands out as a structurally significant amino acid among the constituents. Its significance lies in orienting the Trp8 side chain and contributing to the molecular core’s foundational structure.

Saturation transfer difference (STD) experiments^41^ have also been used to detect and characterize the orientation of small molecule binding to proteins. This has inspired various STD investigations of cationic peptides in LPS micelles to infer important residues.^37^ Differential STD effects were observed from a sample used to record NOESY data in “wet” D_2_O at a relatively low peptide:LPS ratio of 60:1 and ranged in magnitude from 40 to 100% (Figure S35). The most substantial STD effects were evident in the case of the aromatic side chains and lipid methyl protons (ranging from 85% to 100%), as well as the adjacent high-field (HF) proton of the lipid (80%), along with the lipid alpha protons (67%). These outcomes collectively underscore the significance of both lipids and aromatic moieties in the context of binding to LPS. The STD effect to the side chain methyl groups of Ile10 was 50–60% and Thr9–54%. Interestingly the side chain beta and gamma protons for the Orn residues displayed lower STD effects (∼40%) which may suggest they are not as close to LPS or are more transiently engaged. Interestingly, low STD effects were also observed for the positively charged Dab residues in PMB in the presence of LPS micelles.^42^

On the basis of the NMR data observed, laterocidine is therefore predominantly extended and unstructured in solution at ambient temperature. However, in the presence of LPS micelles it becomes compact yielding a hydrophobic core supporting a relatively exposed band of Orn residues positioned, presumably, at the top of the peptide within the hydrophilic regions of LPS. Understanding SAR with reference to the trNOE LPS-bound structure alone is complicated by any potential multimodal activity of laterocidine as well as its plasticity during folding within the LPS environment. Nevertheless, from the trNOE NMR structure (Figure 5), loss of the lipid or the _D_-Tyr2Ala and _D_-Trp3Ala “core residue” changes would reduce hydrophobic-LPS interactions and perturb the packing in the core and spatial arrangement of the ornithine (Orn) side chains. The positively charged region formed by the Orn side chains is probably crucial for hydrophilic interactions with neighboring phosphates, keto acids, or other polar entities. Additionally, the positively charged region might also play a role in less specific, long-range electrostatic interactions during the initial folding stages before reaching binding-competent configurations characterized by hydrophobic orientations and insertions. Trp8Ala substitution was detrimental to activity, possibly due to this residue interacting more directly with LPS, a reasonable inference from the structure (Figure 5). Similarly, the Ile10Ala change would likely impact the long-range packing into the _D_-Tyr2/lipid/_D_-Trp3 triad. The exposed nature (and likely LPS-distal position) of residues Gly11, Gly12, and Asn13 in the macrocycle is consistent with the observation that alanine substitutions at these positions returned most of the activity of the native peptide. Furthermore, their exposed nature in the structure suggests that a range of stereochemical substitutions can be accommodated for physiochemical optimization of second-generation scaffolds.

### Assessment of the *In Vitro* Toxicity

Nephrotoxicity represents a significant limitation in the clinical development of cationic antimicrobial peptides (CAPs).^43^ Therefore, we investigated the *in vitro* cytotoxicity of laterocidine (peptide **1**) and its nonanoyl moiety-containing analogue (peptide **11**) on human kidney proximal tubular (HK-2) cells. Polymyxin B, one of two polymyxins currently used clinically and known to be associated with significant nephrotoxicity, was used as a comparator.^44−46^ HK-2 cells were incubated with concentrations of 0.01 or 0.1 mM of each individual peptide, selected based on the accumulation of polymyxin B in HK-2 cells and associated apoptosis.^46,47^ Interestingly, significant differences in cell viability between polymyxin B and laterocidine and its analogues were observed, especially at the higher concentration (Figure S30). Cells treated with peptide **14** showed markedly higher cell viability (89.3 ± 2.4% [mean ± SD]) at 0.1 mM compared to cells treated with laterocidine (10.3 ± 0.9%) or polymyxin B (6.4 ± 3.6%). These results are consistent with findings for other cationic antimicrobial peptides (CAPs) like polymyxins, where loss of the *N*-terminus fatty acyl chain leads to a substantial reduction in the observed *in vitro* cytotoxicity in HK-2 cells.^46^ Viability of the HK-2 cells was also significantly higher following treatment with 0.1 mM of peptide **25** (46.2 ± 4.4%) or peptide **34** (30.7 ± 3.4%) than with peptide **35** (8.7 ± 2.2%), suggesting a critical role for the aromatic side chain in inducing toxicity. Importantly, the viability of HK-2 cells treated with 0.01 mM of laterocidine (88.5 ± 2.3%) was significantly higher than with polymyxin B (74.8 ± 2.6%) at the same concentration. The five positively charged residues and hydrophobic motif of polymyxin B likely results in stronger electrostatic and hydrophobic interactions with HK-2 cells compared to laterocidine, thereby inducing higher cytotoxicity.^46,48^ Interestingly, the native laterocidine showed significantly higher cell viability compared to peptide **11** (69 ± 5.9%) at the lower concentration.

**Figure 6:**
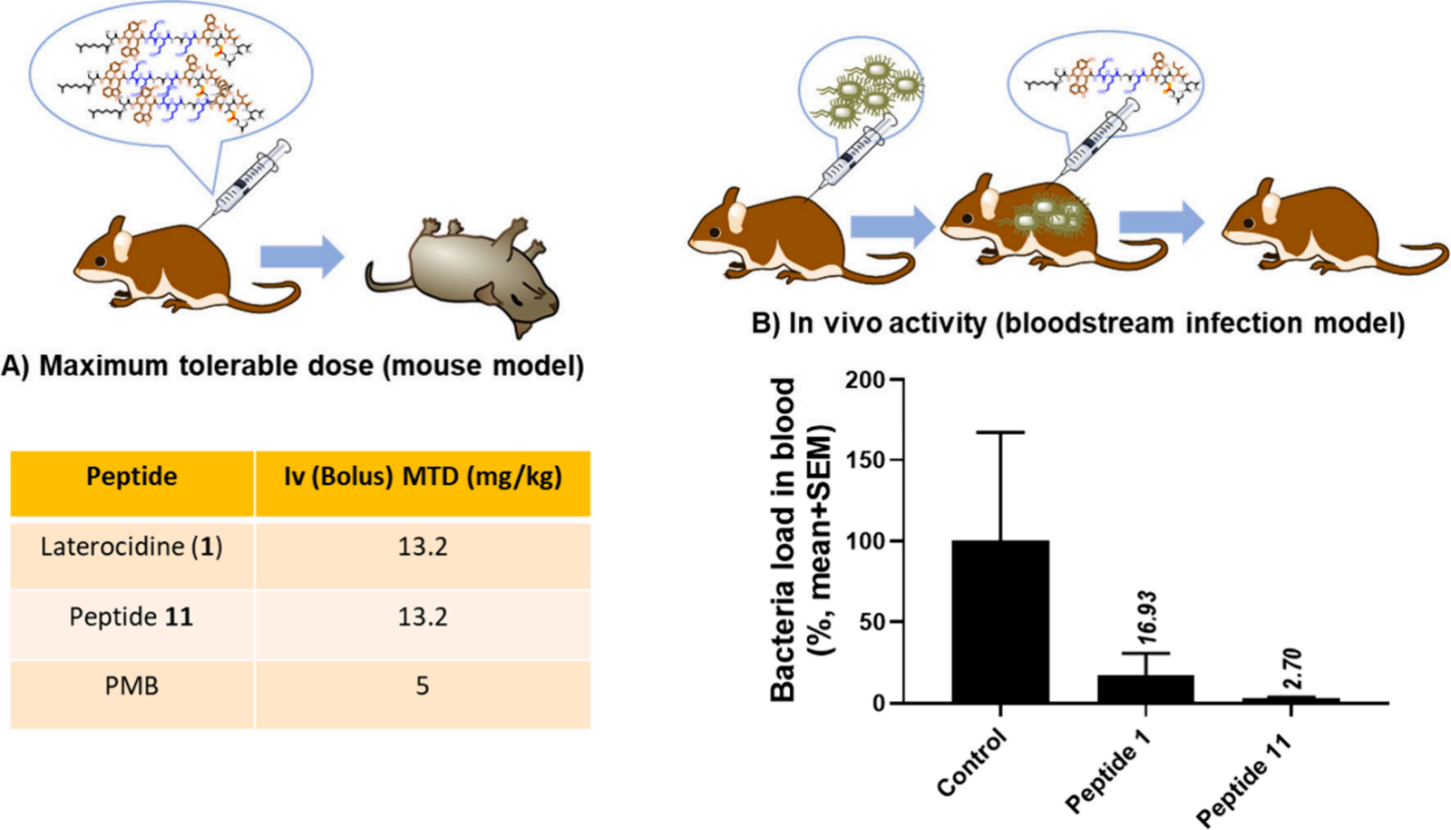
(A) Maximum tolerable dose for peptide 1 (**Native**) and peptide **11**, PMB was calculated using Swiss female mice (weighing 22–28 g and aged 7 weeks). All mice were administered an intravenous bolus of the peptides (mg/kg free base) via a lateral tail vein (≤0.1 mL). (B) Efficacy of laterocidine (peptide **1**) and peptide **11** in a bloodstream infection murine model by *P. aeruginosa* FADDI-PA070. Laterocidine analogues (8 mg/kg, *N* = 3, 4 h time point).

Intrigued by these observations, we subsequently determined the maximum tolerable dose (MTD) and investigated *in vivo* efficacy and of native laterocidine (peptide **1**) and peptide **11** using polymyxin B as a comparator. Importantly, the MTD for both laterocidine and peptide **11** was 13.2 mg/kg, substantially higher than the 5 mg/kg obtained with polymyxin B (Figure 6A). This finding indicates much higher *in vivo* tolerability of laterocidines compared to polymyxin B. Furthermore, employing a murine bloodstream infection model with the multidrug-resistant (MDR) *P. aeruginosa* strain FADDI-PA070, which exhibits resistance to polymyxin, both peptide **1** (at 16.9%, as shown in Figure 6B) and peptide **11** (at 2.7%, as shown in Figure 6B) demonstrated notable efficacy in reducing bacterial populations. Remarkably, peptide **11** demonstrated an 8-fold increase in antimicrobial activity compared to native laterocidine (peptide **1**), suggesting that peptide **11** has the potential to serve as a valuable template for future drug development endeavors.

## Conclusions

Our initial investigations revealed that the ester linkage and positive charges at positions 4, 5, and 7 are crucial for antimicrobial activity. Positions 2 and 3 are crucial in forming its hydrophobic core. Substituting small and hydrophobic amino acids at positions 6, 8, and 10 reduces its activity, while neutral residues at positions 11, 12, and 13 can be replaced without significant loss of activity. It is worth noting that the presence of a fatty acid tail is essential for maintaining its antimicrobial efficacy. Peptide **11**, engineered with a nonanoyl fatty acid, outperformed the native peptide, completely inhibiting the growth of a polymyxin-resistant *P. aeruginosa* strain in static time kill studies.

The mechanistic studies highlight that laterocidine’s mode of action hinges on a dynamic interplay between the peptide and the bacterial outer membrane during bacterial growth. The presence of LPS is found to be essential for facilitating these interactions. NMR analysis revealed laterocidine as unstructured in its free form but adopting a folded conformation when bound to LPS. The high-resolution LPS-bound structure aligns with structure–activity relationship data, offering valuable insights into its mode of action a resource for future chemical exploration and innovation. *In vivo* studies demonstrated that laterocidine exhibits significantly reduced toxicity and efficacy in an infected murine model. This compelling outcome not only underscores the distinctive mechanistic profile inherent to laterocidine but also serves to demarcate it from the last-resort antibiotic polymyxin. This contrast underscores the remarkable potential of laterocidine as a potent agent in the arena of combating microbial infections.

Overall, this study positions laterocidine as a highly promising template for the advancement of future antimicrobial drugs. Its unique mechanism of action and excellent potency establish it as an exceptional contender in the battle against microbial infections. These attributes highlight its tremendous potential as a prime candidate deserving of extensive research and development efforts in the pursuit of novel antimicrobial agents.

## Experimental Section

### Materials and Methods

Piperidine, diisopropylethylamine (DIPEA), trifluoroacetic acid (TFA), and 1H-benzotriazolium-1-[bis(dimethylamino)methylene]-5-chloro-hexafluorophosphate-(1-),3-oxide (HCTU) were obtained from Auspep (Melbourne, Australia). *N*,*N*′-Diisopropylcarbodiimide (DIC), Fmoc-Orn (Boc)-OH, Fmoc-_D_-Orn(Boc)-OH, and (2S,3R)-(Fmoc-amino)-3-azidobutyric acid were obtained from Chem-Impex International (USA). Fmoc-Thr(tBu)-OH was obtained from Mimotopes (Melbourne, Australia) and dimethylformamide (DMF), methanol (MeOH), diethyl ether, dichloromethane (DCM), hydrochloric acid (HCl), and acetonitrile from Merck (Melbourne, Australia). LC–MS grade water and acetonitrile were obtained from ThermoFisher Chemical (Melbourne, Australia). 2-Chlorotrityl chloride-PEG resin was obtained from Chempep (Australia). Triisopropylsilane (TIPS), 2,2′-(ethylenedioxy)diethanethiol (DODT), diphenylphosphorylazide (DPPA), palladium tetrakis(triphenylphosphine), phenylsilane, and diisopropylethylamine (DIPEA) were obtained from Sigma-Aldrich (Castle Hill, Australia).

### General Peptide Synthesis Protocol

Peptide synthesis was undertaken using 2-chlorotrityl chloride resin (0. 1 mmol,100–200 mesh, 0.4–1.0 mmol/g). The first amino acid (Gly13) was loaded using a 6-mol equiv solution of Fmoc-amino acid in DMF (concentration 150 mM), the final loading was determined by a weight gain method, 0.6 mmol/g. The rest of the amino acids from 8 to 12 were built on a Protein Technologies Prelude automated peptide synthesizer using standard Fmoc solid-phase peptide chemistry. Coupling of the Fmoc-amino acids was performed using the default instrument protocol: 3-mol equiv (relative to resin loading) of the Fmoc amino acid and HCTU in DMF and *in situ* activation using 6-mol equiv of DIPEA for 50 min at room temperature. Fmoc deprotection was conducted at room temperature using the default instrument protocol: 20% piperidine in DMF (1 × 5 min, 1 × 10 min).

Esterification and cyclization of peptide: Peptide **7** was synthesized on a Prelude automated synthesizer and dried. With the use of the manual coupling protocol, the Alloc-Gly-OH (5 equiv) was dissolved in dry DMF (concentration 150 mM), then 5 equiv DIC and 0.3 equiv DMAP were added. The activated solution was added to the peptide **7** reaction vessels and bubbled with nitrogen for 6 h. Small-scale cleavage and LC–MS analysis confirmed the successful coupling of Alloc-Gly-OH with the threonine side chain (the reaction was repeated if needed). The remaining sequence (1 to 7) was completed on a Prelude automated peptide synthesizer using standard Fmoc solid-phase peptide chemistry. The linear peptide resin **9** was treated with phenylsilane (10 equiv, 0.123 mL) and a catalytic amount of palladium tetrakis(triphenylphosphine) (0.1 equiv, 12 mg) in DCM for 40 min (the reaction was repeated if needed) to deprotect the alloc group from the Gly *N*-terminal. The protected linear peptide **10** was then cleaved from the resin with 10% hexafluoroisopropanol (HFIP) in DCM (1 × 30 min, 1 × 5 min). This solution was concentrated in a vacuum to give the crude-protected linear peptide. The protected linear peptide was dissolved in DMF (5 mL), then DIPEA 0.6 mmol, 104 μL (6 mol equiv relative to the loading of the resin) and DPPA, 0.3 mmol, 0.65 μL (3 mol equiv relative to the loading of the resin) were added and stirred for 6 h at room temperature. To obtain the crude protected cyclic peptide, the DMF was concentrated under a vacuum and the crude peptide taken up in 10 mL of TFA cleavage cocktail solution (2.5% DODT, 5% TIPS, 92.5% TFA), which was stirred at room temperature for 90 min. TFA was evaporated under a nitrogen stream, and 40 mL of diethyl ether was added to residual TFA to precipitate the peptide. The precipitate was collected by centrifugation, washed twice with diethyl ether (35 mL), and air-dried to give the crude cyclic peptide as a pale-yellow solid. The crude solid was dissolved in Milli-Q water (5 mL) and desalted using a ion-exchange Vari-Pure IPE SAX column. The crude cyclic lipopeptide was then subjected to RP-HPLC purification on a Shimadzu LC system with a “Prominence” diode array detector (214 nm). A Phenomenex Axia column (Luna C8(2), 250 × 21.2 mm i.d., 100 Å, 10 μm) was used with a gradient of 0–60% buffer B over 60 min at a flow rate of 15 mL/min (buffer A: 0.1% TFA/water and buffer B: 0.1% TFA/acetonitrile). The collected fractions were analyzed by LC–MS. A Phenomenex column (Luna C8(2), 100 × 2.0 mm ID) was used, eluting with a gradient of 0–60% solvent B over 10 min at a flow rate of 0.2 mL/min (solvent A: 0.05% TFA/water and solvent B: 0.05% TFA/acetonitrile). Mass spectra were acquired with a scan range of 200–2000 *m*/*z* in the positive ion mode. Pure desired product fractions (95% purity) were combined and lyophilized for 2 days to give the purified cyclic peptide as its corresponding TFA salt.

### Measurement of Minimum Inhibitory Concentrations (MICs) and Time-Kill Kinetics Studies

MICs for laterocidine and its various analogues were determined by broth microdilution^49^ for the following polymyxin-susceptible ATCC and clinical isolates: *P. aeruginosa* FADDI-PA022, FADDI-PA001, FADDI-PA025, FADDI-PA070, FADDI-PA060; *A. baumannii* FADDI-AB134, FADDI-AB034, FADDI-AB030, FADDI-AB174, FADDI-AB241; *K. pneumoniae* FADDI-KP032, FADDI-KP022, BM1, FADDI-KP065, FADDI-KP064, FADDI-KP003; and *E. cloacae* FADDI-EC001, FADDI-EC003, and FADDI-EC006. Experiments were performed with cation-adjusted Mueller–Hinton broth (CAMHB) in 96-well polystyrene microtiter plates. Wells were inoculated with 100 μL of bacterial suspension prepared in CAMHB (containing ∼10^6^ CFU/mL) and 100 μL of CAMHB containing increasing concentrations of polymyxins (0–32 μg/mL). MIC measurements were performed in duplicate, with the MIC being the lowest concentration at which visible growth was inhibited following 18–20 h of incubation at 37 °C. Bacterial killing kinetics was investigated over 24 h as described previously.^50^

### NMR Structural Studies

NMR samples contained 0.75–2.0 mM laterocidine in a 50 mM acetate (*d*_6_) NMR buffer (90% H_2_O:10% D_2_O, pH 4.5 or ∼100% D_2_O, pH 4.5). Experiments were recorded on a Bruker 600 MHz NMR spectrometer equipped with a cryoprobe and Z axis gradient. 2D ^13^C HSQC and 2D ^13^C–^1^H H2BC were recorded using gradients for coherence selection and sensitivity enhancement and additional presaturation of the solvent signal when required. 1D ^1^H, 2D ^1^H–^1^H NOESY (with mixing times 35–200 ms), and 2D ^1^H–^1^H TOCSY experiments (mixing time 70 ms) used an excitation sculpting, DPFGSE sequence for water suppression.^51^ Typical acquisition times for the 2D ^1^H–^1^H NOESY experiments were 524 ms (t2) and 40 ms (t1). For transferred LPS experiments, NMR data was recorded at 23–25 °C which was optimized for the least amide peak overlap. 2D ^1^H–^1^H NOESY spectra for the free peptide were also recorded at 23 °C. Spectra were processed with nmrPipe^52^ or Topspin and analyzed with Xeasy^53^ or NMRFAM Sparky.^54^ Backbone assignments were made using the standard sequential NOE based assignment strategy using 2D ^1^H–^1^H TOCSY and 2D ^1^H–^1^H NOESY data. Side chain assignments were further aided using the 2D ^13^C–^1^H H2BC experiment. 1D ^1^H STD experiments were recorded using excitation sculpting for water suppression. A cascade of 40 selective Gaussian-shaped pulses (50 ms each, total saturation of 0.5–2.5 s) were applied at an “on” saturation frequency of −4 ppm (for selective LPS saturation) and “off” frequency of 100 ppm with an additional 0.5 s relaxation delay.

Structures were calculated in a semiautomatic manner assigning the NOESY peaks using CYANA version 3.97 and the standard NOE assign macro.^55^ Three hundred structures were calculated at each of the seven cycles and the top 20 carried forward to the next cycle. The ^1^H chemical shift tolerance was set to 0.01 ppm in both dimensions because of significant signal overlap. The “dref” was set to 5.0 Å and the upper distance loosened from the standard 5.5 Å to 6 Å during automatic calibration of upper bound distance restraints to compensate for the effects of spin diffusion on the LPS micelle. New CYANA library entries were added as described^56^ by first building the coordinates in Chimera.^57^ The library file for the D amino acids was made by inverting the sign of the *y* coordinate of the standard residue within CYANA. The cyclized ester bond was constructed using upper limit NOEs between the side chain oxygen of the threonine and the glycine carbonyl carbon with a scaling factor of 100 using the “link” command. The Ile10CO-HNGly13 hydrogen bond inferred from early calculations was included in the final structure. The CYANA distance restraints were then used during subsequent refinement in XplorNIH using internal variable torsion angle dynamics utilizing a PC6 integration scheme and simulated annealing. In this, an initial run was performed starting at 30,000 K which served to generate a folded peptide template. This structure was then subjected to simulated annealing comprising a heating stage at 3000 K then cooling to 15 K followed by a final Powell minimization. 100 structures were calculated, and the top 41 were selected (see Table S4). The final force constant for distance restraints was 30 kcal mol^–1^. _D_-Amino acids were incorporated by swapping the two central atoms in the HA-N-C-CB improper torsion. The XplorNIH “protein.par” parameter set was used in the refinement with modifications and further parametrization of the lipid tail.

### Effect of Laterocidine on Bacterial Membrane Integrity by Flow Cytometry

Bacterial pellets of *P. aeruginosa* PAO1 taken from the time-kill studies at 1 h post exposure to 2 μg/mL (1× MIC) and 16 μg/mL (8× MIC) laterocidine (i.e., peptide **1**) were resuspended in sterile saline (0.9 w/v NaCl). Samples were assessed using BL-1 and BL-4 blue laser detection channels of the ACEA NovoCyte high-performance benchtop flow cytometer (ACEA Biosciences, Santa Clara, CA, USA) with preset thresholds; forward-scatter (FSC-H) and side-scatter (SSC-H) of >1000 units, events/sec < 1000, and maximum acquisition of 20,000 events. The live cell impermeant fluorophore propidium iodide (PI) (Sigma-Aldrich, Castle Hill, NSW, Australia) was used to assess loss of membrane integrity (Ex/Em 488/660–690 nm) and the redox probe 5-cyano-2,3-ditolyl tetrazolium chloride (CTC) (Sigma-Aldrich, Castle Hill, NSW, Australia) used to assess loss of cellular respiration (Ex/Em 450/630 nm). The voltage-sensitive fluorophore bis(1,3-dibutylbarbituric acid) trimethine oxonol (DiBAC) (Sigma-Aldrich, Castle Hill, NSW, Australia) assessed membrane depolarization (Ex/Em 488/660–690 nm). Intracellular oxidative stress was measured using CR-G (CellROX Green) (Sigma-Aldrich, Castle Hill, NSW, Australia, Ex/Em 488/660–690 nm).^58^ Appropriately diluted samples (600 μL) were stained separately with 12.5 μM PI, 2.5 mM CTC, 6 μM DiBAC, and 2.5 μM CR-G. Samples stained with both PI and DiBAC were incubated for 2 min and immediately analyzed. Samples stained with both CTC and CR-G were incubated for 45 min at RTP, and excess dye was pelleted out prior to analysis. Fluorescence intensity data were analyzed and log transformed using the NovoExpress software (V2.1, ACEA Biosciences, USA).^34^

### Assessment of the Viability of Human Kidney Proximal Tubular Cells (HK-2)

The fluorescence microscopy method used to culture HK-2 cells and the use of fluorescence microscopy to determine the percent cell viability (%) following peptide treatment are the same as published previously.^59,60^

### Animal Experiments

All animal studies were approved by the Monash University Animal Ethics Committee and involved female Swiss mice (22–28 g, 7-week-old) housed in microisolators within a PC2 animal laboratory. Mice were kept under a 12/12 h dark/light cycle at 20–24 °C and 50–70% ambient humidity.

#### Acute Toxicity in Mice

For the acute toxicity studies, peptide **11** and laterocidine solutions were prepared in 0.9% saline and stored at 4 °C before use. All mice were administered an intravenous bolus of one peptide (mg/kg free base) via a lateral tail vein (≤0.1 mL). Following the injection, mice were released back into their respective home cages and individually monitored for 24 h for signs of clinical toxicity. Mice displaying any signs of toxicity were humanely euthanized according to the recommended Euthanasia/Humane Experimental End point Criteria. The maximum dose (mg/kg free base) that did not result in any side effects was determined as the maximal tolerable dose (MTD, *N* = 3, 4 h time point).^61^

#### Neutropenic Murine Bloodstream Infection Model

The neutropenic bloodstream infection model was undertaken as previously described.^39^ Mice were rendered neutropenic through intraperitoneal injections of cyclophosphamide administered 4 days (150 mg/kg) and 1 day (100 mg/kg) prior to inoculation. Bloodstream infection was established by administering a 50 μL bolus of early log-phase bacterial suspension (4 × 10^8^ CFU/mL). Laterocidine, peptide **11**, and polymyxin B solutions were prepared in sterile saline at a concentration of 1 mg/mL (free base). Two hours postinoculation, mice (*n* = 3) were injected intravenously with either polymyxin B at 4 mg/kg (free base), peptide **11** at 8 mg/kg (free base), laterocidine at 8 mg/kg (free base), or saline (control mice). Blood was collected immediately after (0 h) and 4 h after drug administration, serially diluted with sterile saline, and appropriately diluted samples manually plated on nutrient agar prior to overnight incubation at 37 °C. Colonies were counted manually and the bacterial load (log_10_ CFU/mL) in the blood of each mouse calculated. *In vivo* efficacy was calculated as the difference between the log_10_ CFU/mL blood values of treated and control mice 4 h postdosing (Δlog_10_ CFU/mL blood = log_10_ [treated] CFU/mL blood–log_10_[control] CFU/mL blood).^54^

## Supporting Information Available

Methods, synthesis of peptides and their characterization data, LC-MS and NMR spectra (PDF)
