# Supplementary material for: Exploring Structure–Activity Relationships and Modes of Action of Laterocidine
Source: ACS Cent Sci. 2024 Aug 6;10(9):1703–17. doi: 10.1021/acscentsci.4c00776 (PMC11428279; doi:10.1021/acscentsci.4c00776)
Supplement: Supplementary file 1 — oc4c00776_si_001.pdf [file oc4c00776_si_001.pdf]

Supporting Information

Exploring Structure-Activity Relationships and Modes of Action of Laterocidine

Varsha J. Thombare,[†a] James D. Swarbrick,[†a] Mohammad A. K. Azad,[b] Yan Zhu,[b] Jing Lu,[b] Heidi Y. Yu,[b] Hasini Wickremasinghe,[b] Xiaoji He,[b] Mahimna Bandiatmakur,[b] Rong Li,[b] Phillip J. Bergen,[b] Tony Velkov,[a] Jiping Wang,[b] Kade D. Roberts,[b] Jian Li,[b]* Nitin A. Patil[b]*

a. Biomedicine Discovery Institute, Infection Program and Department of Pharmacology, Monash University, Melbourne, VIC 3800, Australia.

b. Biomedicine Discovery Institute, Infection Program and Department of Microbiology, Monash University, Melbourne, VIC 3800, Australia.

† Equal first authors.

* Corresponding authors:

Dr Nitin Patil at 15 Innovation Walk, Monash University, Clayton campus, Melbourne 3800, Australia. Tel: +61 3 9905 1015; Email: nitin.patil@monash.edu

Prof Jian Li at 19 Innovation Walk, Monash University, Clayton campus, Melbourne 3800, Australia. Email: Jian.Li@monash.edu

This file includes the following:

1. **Methods** **S3**
2. **Synthesis of Alloc-Gly-OH** **S3**
   Figure S1
3. **Peptide structures, and characterization** **S4**
4. **HPLC and mass spectrometry data** **S18**
   Figures S3 to S29
5. **Antimicrobial activity of selected analogues** **S44**
   Table S1 and S2
6. **In vitro toxicity of selected analogues** **S44**
   Figure S30
7. **NMR data** **S45**
   Figures S31 to S37
   Table S3 to S6
8. **References** **S71**

Methods

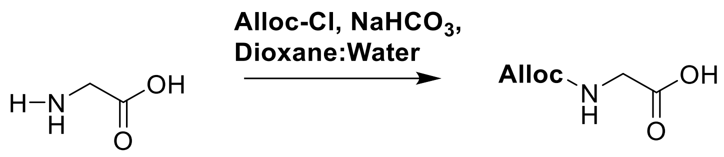

Figure S1: Synthesis of Alloc-Gly-OH.

Synthesis of Alloc-Gly-OH: 2-aminoacetic acid (5g, 66.6 mmol) and $NaHCO_3$ (11.19 g 133 mmol) were dissolved in water 100 mL and dioxane (20ml) and reaction mixture stirred at 0 °C. Then allyl chloroformate (7.10 mL, 66.6 mmol) was added dropwise and mixture was stirred for overnight at room temperature. The reaction mixture was added to Et_2O and 1 M HCl and extracted with AcOEt. The organic layer was washed with brine, dried over $MgSO_4$, and evaporated under reduced pressure. Crude was purified by flash chromatography with 10 % Methanol/DCM (8 g, 75 % yield). ESI-MS analysis of peak at 6.93 min: m/z (monoisotopic) [M+1H]1+ 160.25, [M+K]39+ 199.30. Calculated mass (monoisotopic) for Alloc-Gly-OH ($C_6H_9NO_4$) 160.06. ^{1}H NMR (400 MHz, $CDCl_3$) δ 6.87 (s, 1H), 5.98 – 5.88 (m, 1H), 5.29 (dd, J = 33.8, 13.7 Hz, 2H), 4.62 (d, J = 4.3 Hz, 2H), 4.05 (d, J = 5.4 Hz, 2H). ^{13}C NMR (101 MHz, $CDCl_3$) δ 174.49, 156.41, 132.38, 118.18, 77.35, 77.03, 76.72, 66.20, 42.47.

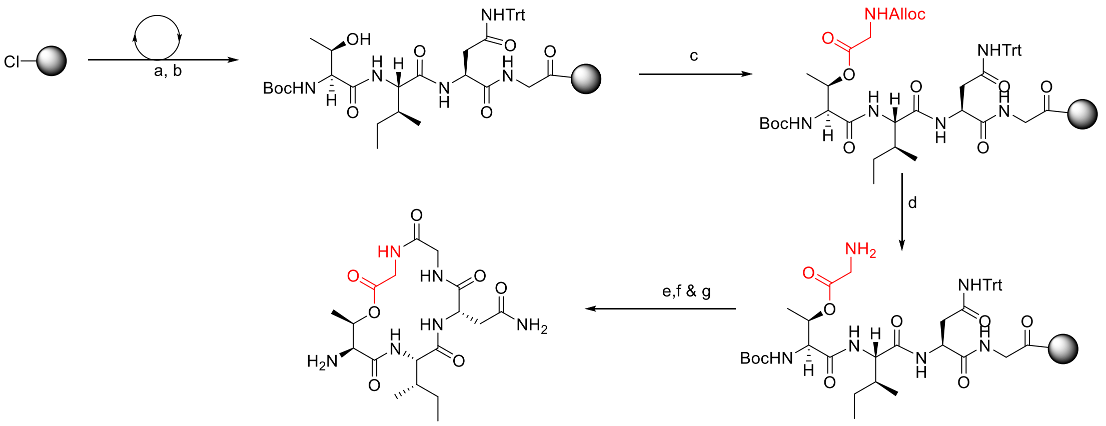

Figure S2: Synthesis of Peptide **13.** Fmoc-AA-OH (3 eq), HCTU (3 eq), DIPEA (6 eq), in DMF 50 min; **b.** 20 % piperidine/DMF (1 × 5 min, 1 × 10 min); **c.** Alloc-Gly-OH (5 eq), DIC (5 eq), DMAP (0.3 eq) in DMF; **d.** Palladium tetrakis(triphenylphosphine) (0.1 eq), $PhSiH_3$ (10

eq), in DCM 40 min; **e.** 10 % HFIP in DCM (1 × 30 min, 1 × 5 min); **f.** DPPA, DIEA in DMF 6 h**; g.** TFA:TIPS:DODT:H_2O (92.5:2.5:2.5:2.5) 90 min.

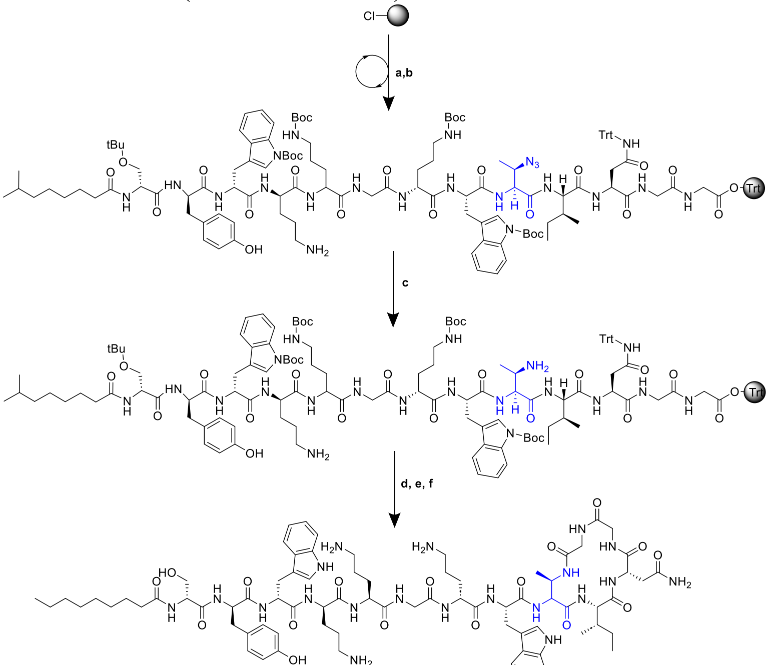

17

Figure S3: Synthesis of Peptide **17.** a. Fmoc-AA-OH (3 eq), HCTU (3 eq), DIPEA (6 eq), in DMF 50 min; b. 20 % piperidine/DMF 15 min; c. 2 M DTT, 1 M DIEA, DMF, RT; d. 10 % HFIP in DCM; e. DPPA, DIEA in DMF; f. TFA:TIPS:DODT:H_2O (92.5:2.5:2.5:2.5).

Laterocidine Peptide 1

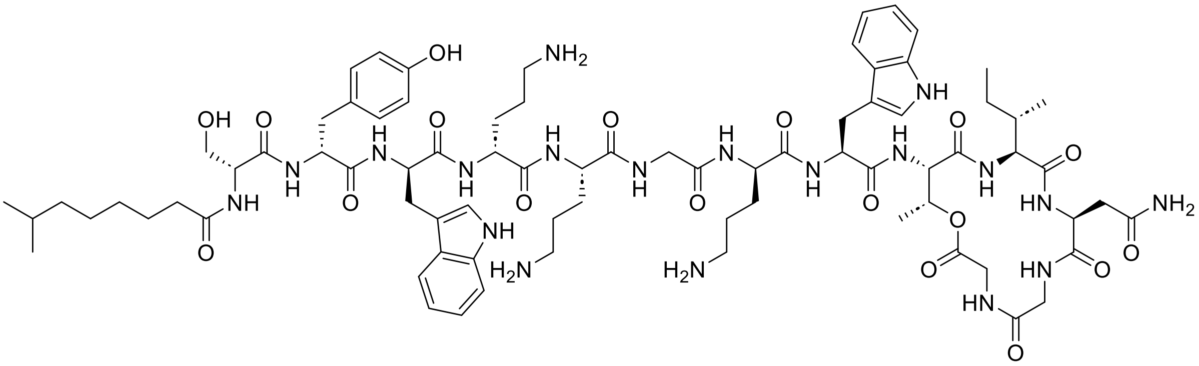

TFA salt was obtained in a yield of 27.4 mg (17.07 %), retention time (tR) at 214 nm = 13.48 min (purity: 95.12 %). ESI-MS analysis of peak at 13.48 min: m/z (monoisotopic) [M+2H]2+ 803.50, [M+3H]3+ 536.10. Calculated mass (monoisotopic) for Laterocidine ($C_{78}H_{113}N_{19}O_{18}$) 1605.88. ^1H NMR (600 MHz, DMSO) δ 10.69 (d, J = 19.4 Hz, 2H), 9.31 (d, J = 6.2 Hz, 1H), 9.09 (s, 1H), 8.26 – 8.20 (m, 2H), 8.15 (s, 1H), 7.99 (t, J = 7.7 Hz, 3H), 7.92 (d, J = 18.0 Hz,

4H), 7.82 (d, J = 8.5 Hz, 1H), 7.77 (d, J = 7.3 Hz, 1H), 7.62 (s, 6H), 7.56 (d, J = 29.1 Hz, 4H), 7.49 (dd, J = 13.3, 8.0 Hz, 2H), 7.29 (s, 1H), 7.26 – 7.22 (m, 3H), 7.15 (t, J = 7.9 Hz, 1H), 7.05 (d, J = 9.5 Hz, 3H), 6.99 – 6.95 (m, 2H), 6.90 – 6.87 (m, 2H), 6.80 (d, J = 8.1 Hz, 3H), 6.48 (d, J = 8.2 Hz, 2H), 5.09 (s, 1H), 5.05 (s, 1H), 4.66 (d, J = 5.7 Hz, 1H), 4.60 (d, J = 8.9 Hz, 1H), 4.41 (d, J = 21.7 Hz, 2H), 4.34 – 4.23 (m, 4H), 4.18 (d, J = 6.8 Hz, 1H), 4.04 (s, 1H), 3.94 (dd, J = 16.7, 8.3 Hz, 1H), 3.82 (s, 1H), 3.77 (d, J = 11.2 Hz, 2H), 3.67 (d, J = 12.9 Hz, 1H), 3.49 (d, J = 13.5 Hz, 1H), 3.39 (d, J = 40.1 Hz, 3H), 3.06 (t, J = 12.7 Hz, 3H), 2.86 (s, 2H), 2.68 (s, 4H), 2.61 – 2.57 (m, 1H), 2.53 (t, J = 12.6 Hz, 3H), 2.02 (dd, J = 13.0, 6.9 Hz, 2H), 1.64 (s, 3H), 1.46 (d, J = 18.9 Hz, 8H), 1.40 – 1.34 (m, 4H), 1.21 (s, 2H), 1.03 (d, J = 6.6 Hz, 2H), 1.01 (d, J = 6.1 Hz, 3H), 0.79 – 0.76 (m, 7H), 0.74 (d, J = 6.6 Hz, 7H).

Peptide 11:

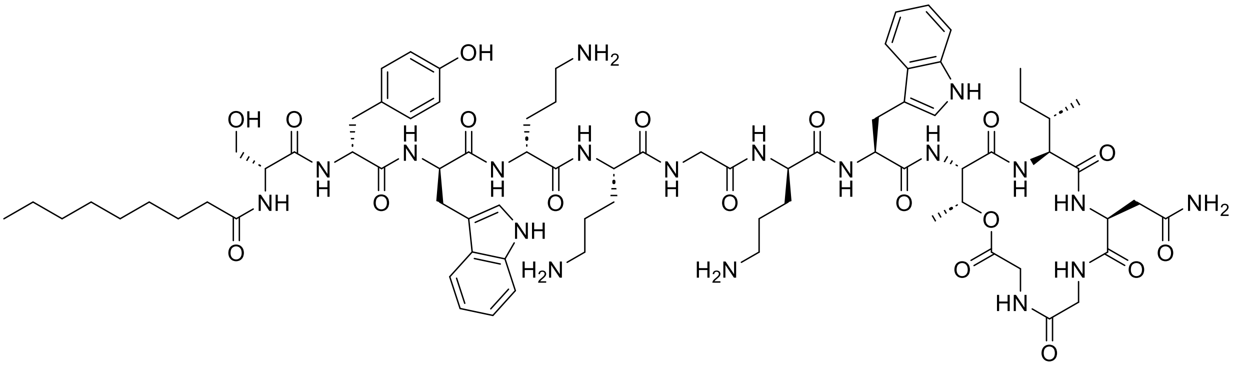

TFA salt was obtained in a yield of 15.2 mg (9.47 %), retention time (t_R) at 214 nm = 13.62 min (purity: 98.79 %). ESI-MS analysis of peak at 13.63 min: *m/z* (monoisotopic) $[M+H]^+$ 1606.25, $[M+2H]^{2+}$ 803.50, $[M+3H]^{3+}$ 536.10. Calculated mass (monoisotopic) for Peptide **11** ($C_{78}H_{113}N_{19}O_{18}$) 1605.88.

Peptide 12:

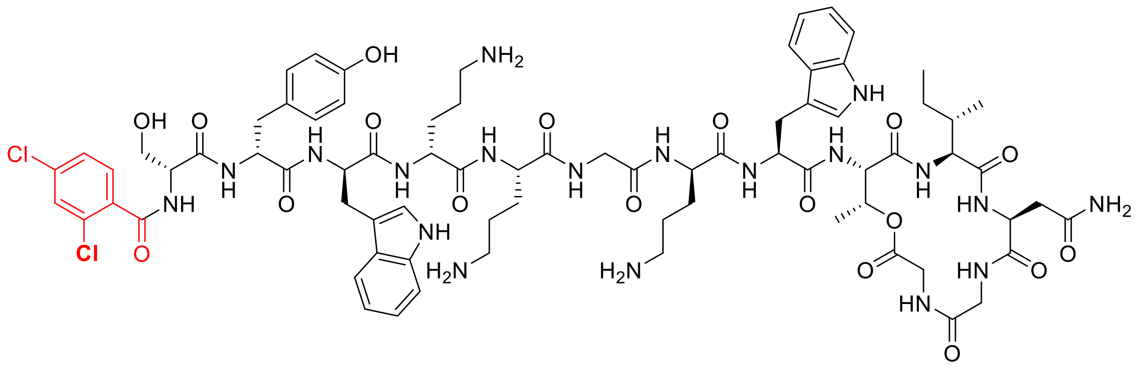

TFA salt was obtained in a yield of 14.5 mg (8.85 %), retention time (t_R) at 214 nm = 12.76 min (purity: 97.74 %). ESI-MS analysis of peak at 12.76 min: *m/z* (monoisotopic) $[M+H]^+$ 1638.00, $[M+2H]^{2+}$ 820.00, $[M+3H]^{3+}$ 547.00. Calculated mass (monoisotopic) for Peptide **12** ($C_{76}H_{99}Cl_2N_{19}O_{18}$) 1638 .64.

Peptide 13:

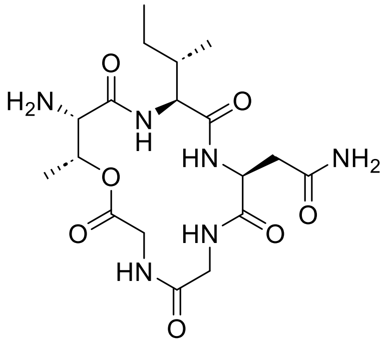

TFA salt was obtained in a yield of 3.9 mg (8.38 %), retention time (t_R) at 214 nm = 9.57 min (purity: 95.54 %). ESI-MS analysis of peak at 9.57 min: *m/z* (monoisotopic) $[M+H]^+$443.35, $[M+Na]^+$ 465.35. Calculated mass (monoisotopic) for Peptide **13** ($C_{69}H_{97}N_{19}O_{17}$) 443.22.

Peptide 14:

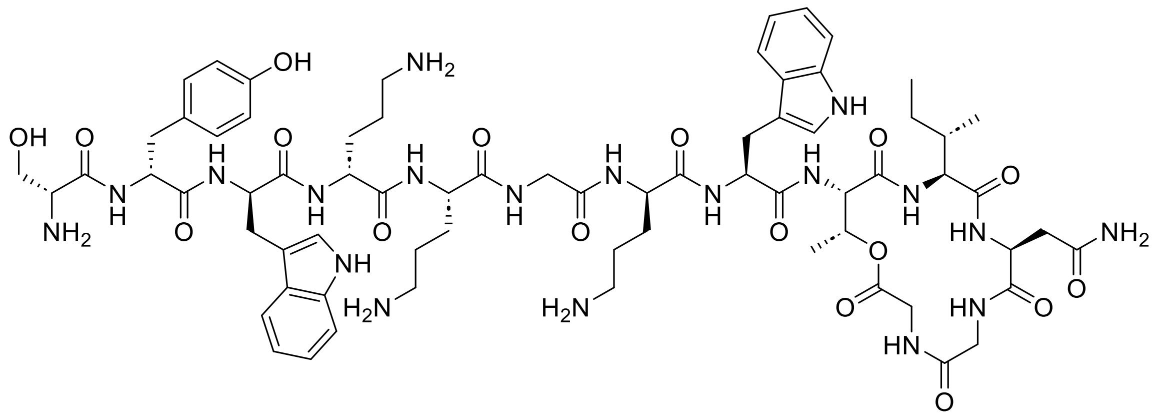

TFA salt was obtained in a yield of 4.2 mg (2.94 %), retention time (t_R) at 214 nm = 11.16 min (purity: 95.45 %). ESI-MS analysis of peak at 11.16 min: *m/z* (monoisotopic) $[M+H]^+$ 1465.20, $[M+2H]^{2+}$ 733.50, $[M+3H]^{3+}$ 489.30. Calculated mass (monoisotopic) for Peptide **14** ($C_{69}H_{97}N_{19}O_{17}$) 1464.65.

Peptide 15:

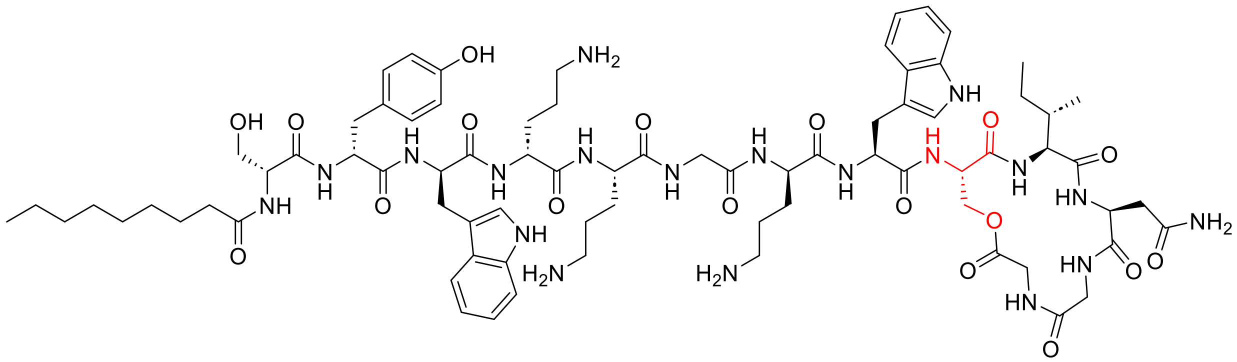

TFA salt was obtained in a yield of 28.2 mg (17.71 %), retention time (t_R) at 214 nm = 13.52 min (purity: 95.04 %). ESI-MS analysis of peak at 13.52 min: *m/z* (monoisotopic) $[M+H]^+$ 1592.30, $[M+2H]^{2+}$ 796.55, $[M+3H]^{3+}$ 531.45. Calculated mass (monoisotopic) for Peptide **15** ($C_{78}H_{114}N_{20}O_{17}$) 1592.86.

Peptide 16:

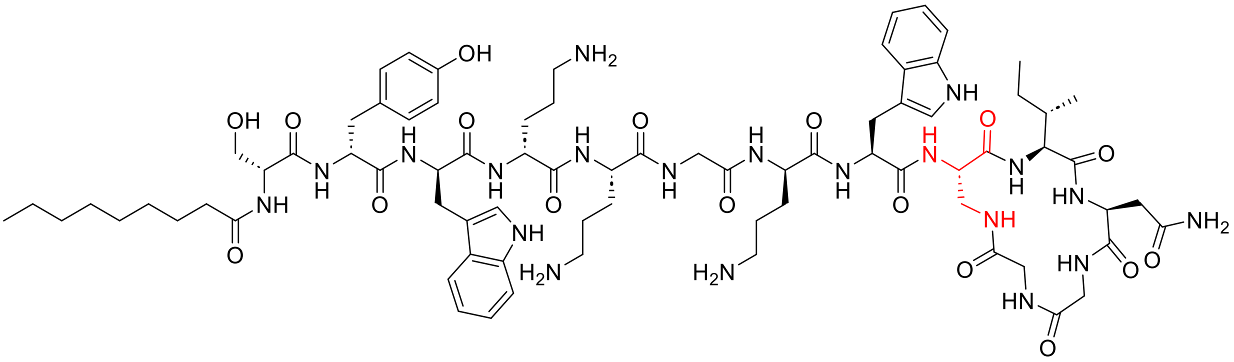

TFA salt was obtained in a yield of 2.7 mg (2.69 %), retention time (t_R) at 214 nm = 13.23 min (purity: 99.5 %). ESI-MS analysis of peak at 13.23 min: *m/z* (monoisotopic) $[M+H]^+$ 1591.45, $[M+2H]^{2+}$ 796.15, $[M+3H]^{3+}$ 531.15. Calculated mass (monoisotopic) for Peptide **16** ($C_{78}H_{114}N_{20}O_{17}$) 1590.86.

Peptide 17:

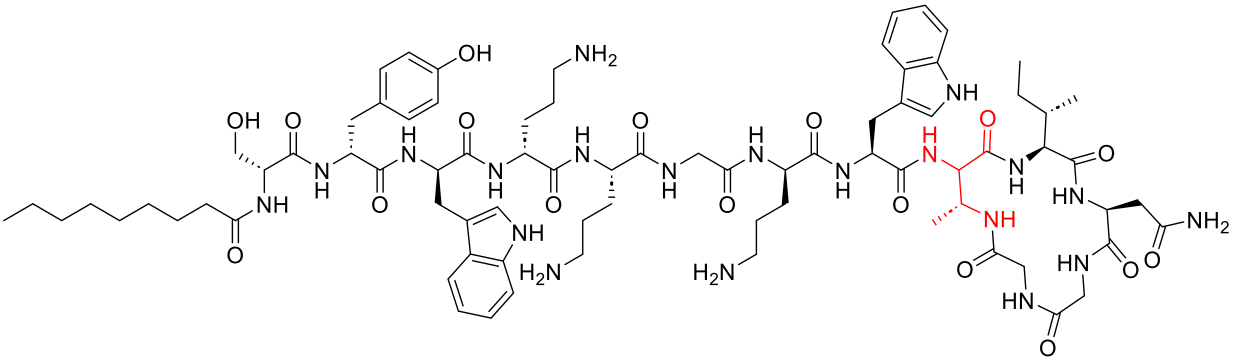

TFA salt was obtained in a yield of 51.9 mg (37.34 %), retention time (t_R) at 214 nm = 13.13 min (purity: 95.74 %). ESI-MS analysis of peak at 13.13 min: *m/z* (monoisotopic) $[M+H]^+$ 1605.50, $[M+2H]^{2+}$ 803.25, $[M+3H]^{3+}$ 535.90. Calculated mass (monoisotopic) for Peptide **17** ($C_{78}H_{113}N_{20}O_{17}$) 1604.87.

Peptide 18:

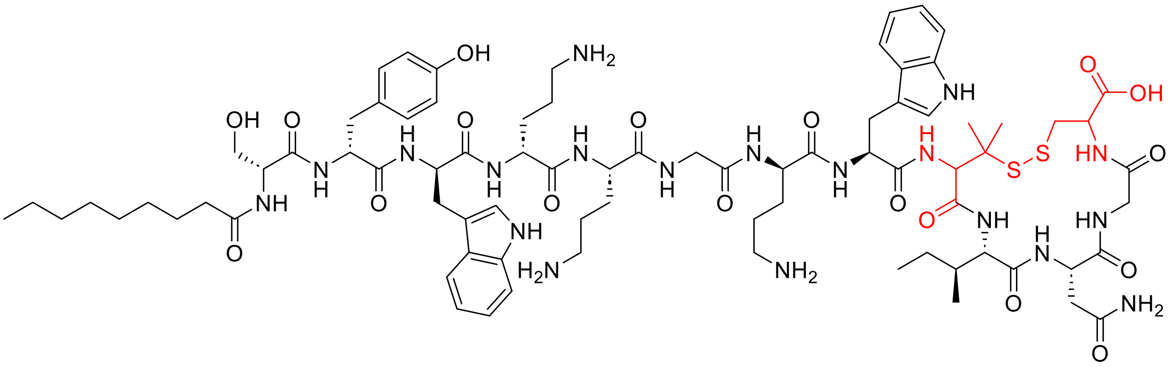

TFA salt was obtained in a yield of 61.2 mg (36.06 %), retention time (t_R) at 214 nm = 13.52 min (purity: 96.48 %). ESI-MS analysis of peak at 13.52 min: *m/z* (monoisotopic) $[M+H]^+$ 1697.35, $[M+2H]^{2+}$ 849.10, $[M+3H]^{3+}$ 566.50. Calculated mass (monoisotopic) for Peptide **18** ($C_{78}H_{113}N_{20}O_{17}$) 1697.83.

Peptide 19:

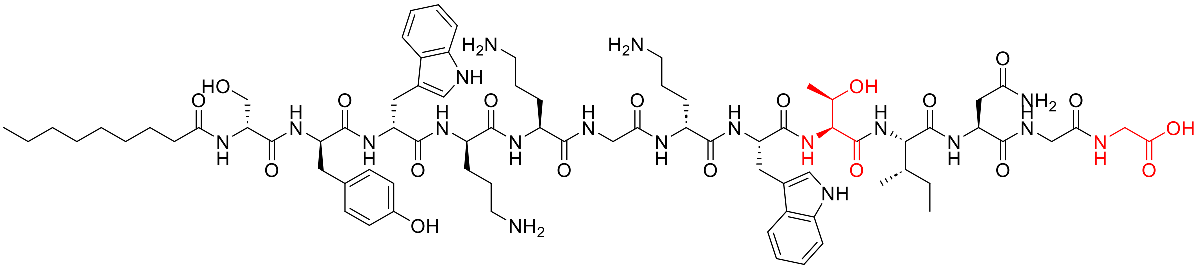

TFA salt was obtained in a yield of 41.3 mg (25.47 %), retention time (t_R) at 214 nm = 13.52 min (purity: 95.18 %). ESI-MS analysis of peak at 13.52 min: *m/z* (monoisotopic) $[M+H]^+$ 1624.50, $[M+2H]^{2+}$ 812.65, $[M+3H]^{3+}$ 542.15. Calculated mass (monoisotopic) for Peptide **19** ($C_{78}H_{113}N_{19}O_{18}$) 1621.89.

Peptide 20:

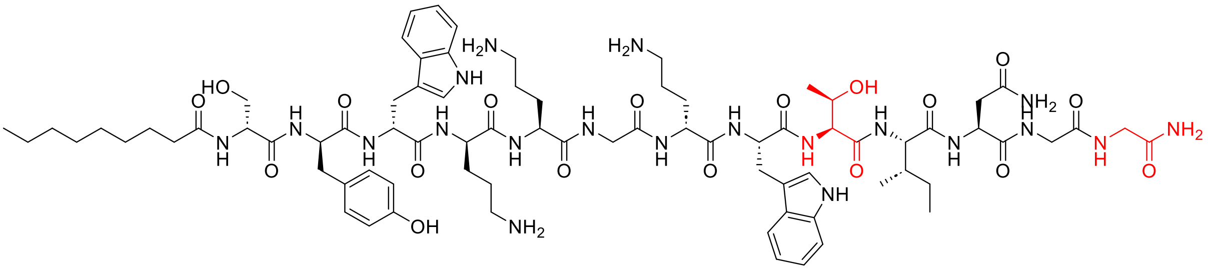

TFA salt was obtained in a yield of 81.9 mg (50.46 %), retention time (t_R) at 214 nm = 13.09 min (purity: 96.97 %). ESI-MS analysis of peak at 13.09 min: *m/z* (monoisotopic) $[M+H]^+$ 1623.45, $[M+2H]^{2+}$ 812.25, $[M+3H]^{3+}$ 541.80. Calculated mass (monoisotopic) for Peptide **20** ($C_{78}H_{114}N_{20}O_{17}$) 1623.87.

Peptide 21:

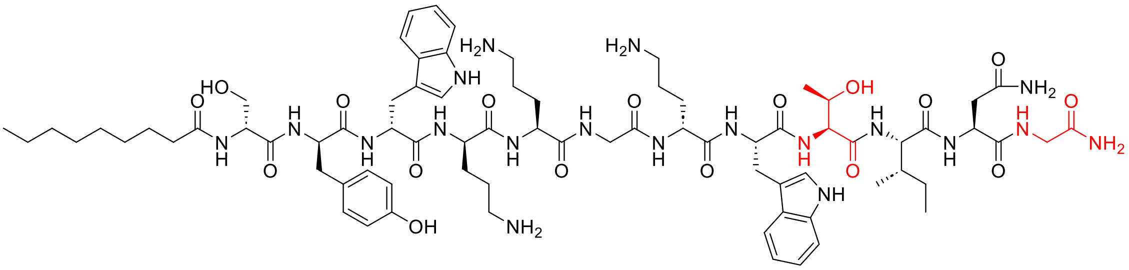

TFA salt was obtained in a yield of 70.7 mg (45.17 %), retention time (t_R) at 214 nm = 13.14 min (purity: 97.38 %). ESI-MS analysis of peak at 13.14 min: *m/z* (monoisotopic) $[M+H]^+$ 1566.45, $[M+2H]^{2+}$ 783.70, $[M+3H]^{3+}$ 522.75. Calculated mass (monoisotopic) for Peptide **21** ($C_{76}H_{113}N_{19}O_{17}$) 1565.86.

Peptide 22:

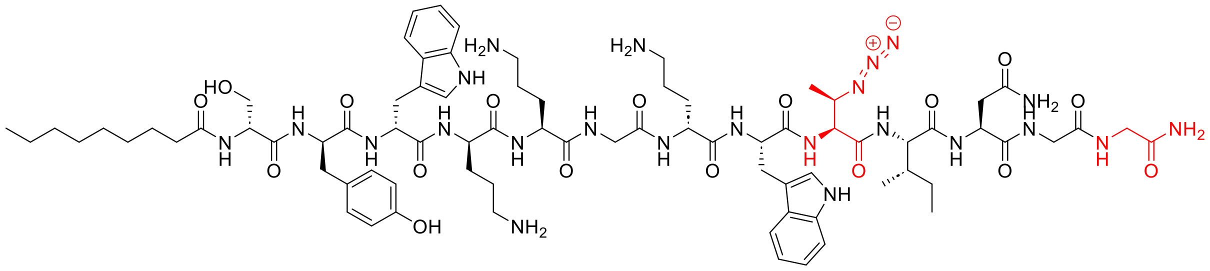

TFA salt was obtained in a yield of 24.8 mg (15.05 %), retention time (t_R) at 214 nm = 13.23 min (purity: 95.88 %). ESI-MS analysis of peak at 13.23 min: *m/z* (monoisotopic) $[M+H]^+$ 1648.40, $[M+2H]^{2+}$ 824.55, $[M+3H]^{3+}$ 550.15. Calculated mass (monoisotopic) for Peptide **22** ($C_{78}H_{115}N_{23}O_{17}$) 1647.89.

Peptide 23:

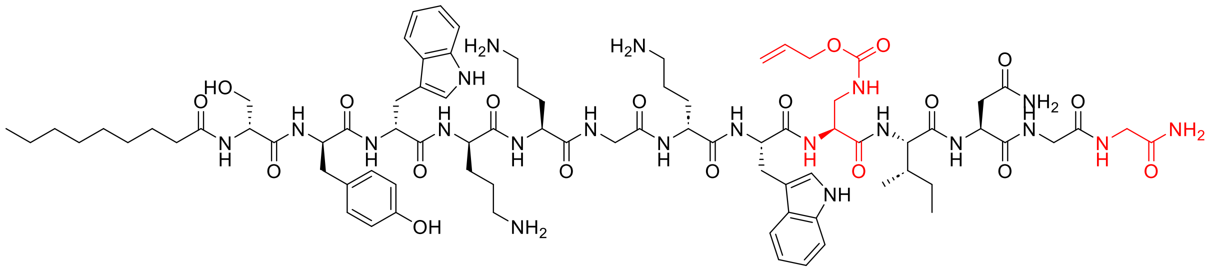

TFA salt was obtained in a yield of 84.4 mg (49.91 %), retention time (t_R) at 214 nm = 13.28 min (purity: 95.81 %). ESI-MS analysis of peak at 13.28 min: *m/z* (monoisotopic) $[M+H]^+$ 1692.55, $[M+2H]^{2+}$ 846.75, $[M+3H]^{3+}$ 564.85. Calculated mass (monoisotopic) for Peptide **23** ($C_{81}H_{119}N_{21}O_{19}$) 1691.91.

Peptide 24:

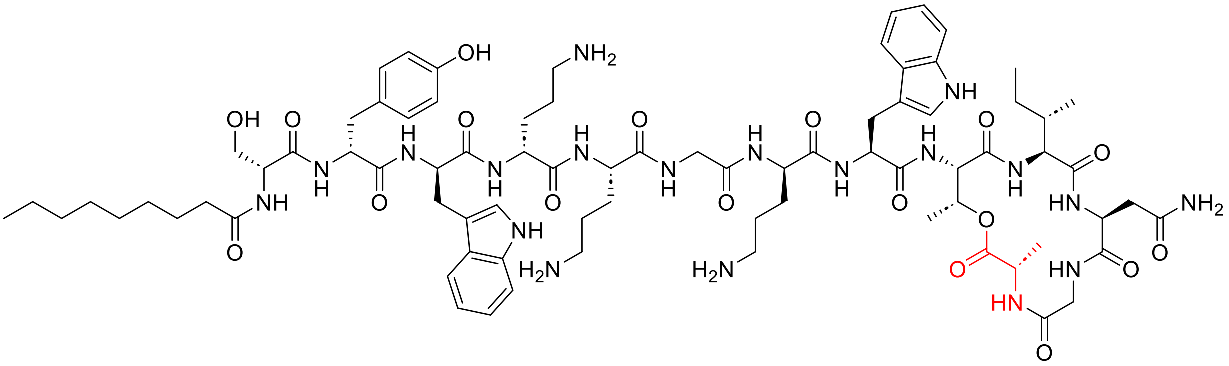

TFA salt was obtained in a yield of 5.5 mg (3.39 %), retention time (t_R) at 214 nm = 10.75 min (purity: 96.08 %). ESI-MS analysis of peak at 10.75 min: *m/z* (monoisotopic) $[M+H]^+$ 1620.35, $[M+2H]^{2+}$ 810.60, $[M+3H]^{3+}$ 540.80. Calculated mass (monoisotopic) for Peptide **24** ($C_{79}H_{115}N_{19}O_{18}$) 1618.90.

Peptide 25:

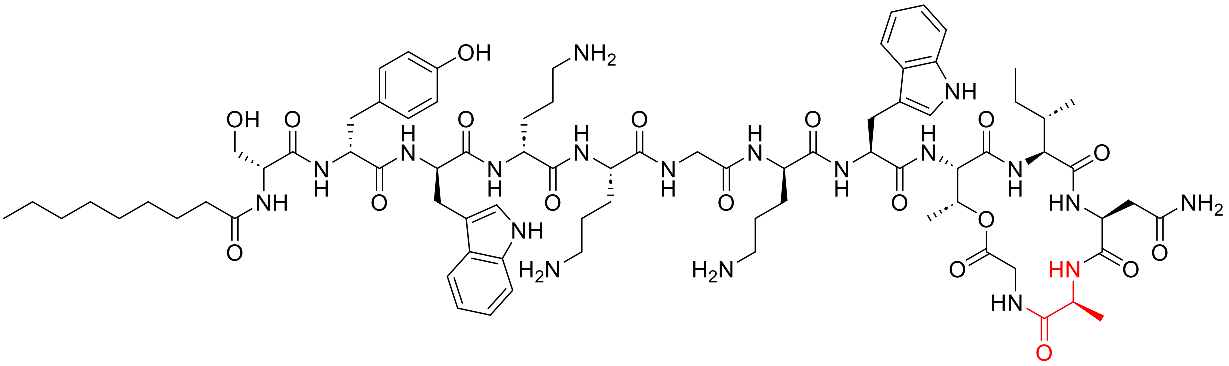

TFA salt was obtained in a yield of 7.4 mg (4.57 %), retention time (t_R) at 214 nm = 10.71 min (purity: 95.20 %). ESI-MS analysis of peak at 10.71 min: *m/z* (monoisotopic) $[M+H]^+$ 1619.35, $[M+2H]^{2+}$ 810.55, $[M+3H]^{3+}$ 540.80. Calculated mass (monoisotopic) for Peptide **25** ($C_{79}H_{115}N_{19}O_{18}$) 1618.90.

Peptide 26:

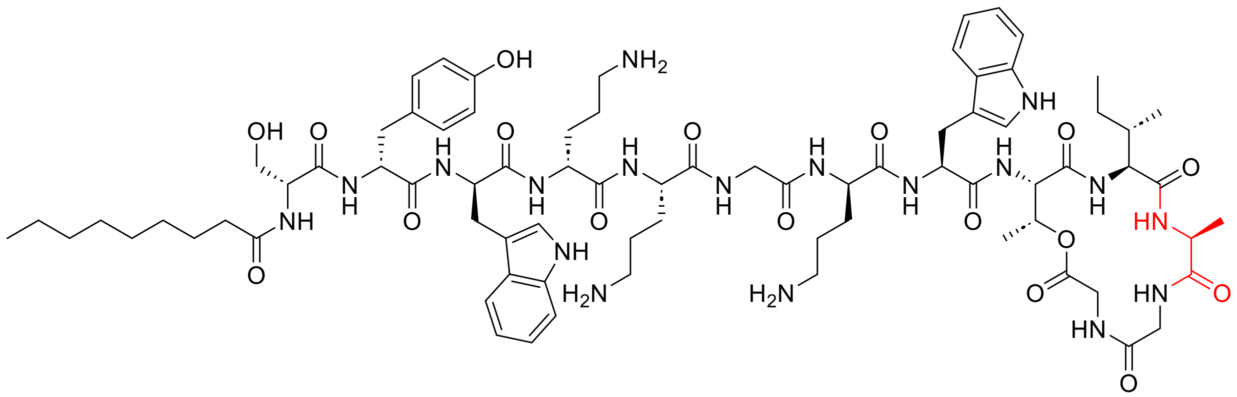

TFA salt was obtained in a yield of 3.8 mg (2.43 %), retention time (t_R) at 214 nm = 13.94 min (purity: 98.81 %). ESI-MS analysis of peak at 13.94 min: *m/z* (monoisotopic) $[M+H]^+$ 1563.25, $[M+2H]^{2+}$ 782.05, $[M+3H]^{3+}$ 521.70. Calculated mass (monoisotopic) for Peptide **26** ($C_{77}H_{112}N_{18}O_{17}$) 1561.85.

Peptide 27:

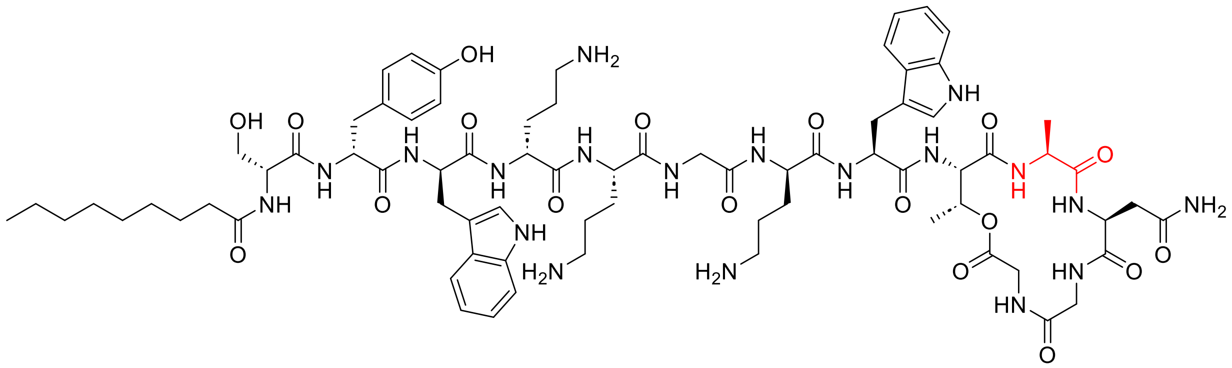

TFA salt was obtained in a yield of 30.1 mg (19.27 %), retention time (t_R) at 214 nm = 13.30 min (purity: 97.67 %). ESI-MS analysis of peak at 13.30 min: *m/z* (monoisotopic) $[M+H]^+$ 1563.12, $[M+2H]^{2+}$ 782.50, $[M+3H]^{3+}$ 522.05. Calculated mass (monoisotopic) for Peptide **27** ($C_{75}H_{107}N_{19}O_{18}$) 1562.80.

Peptide 28:

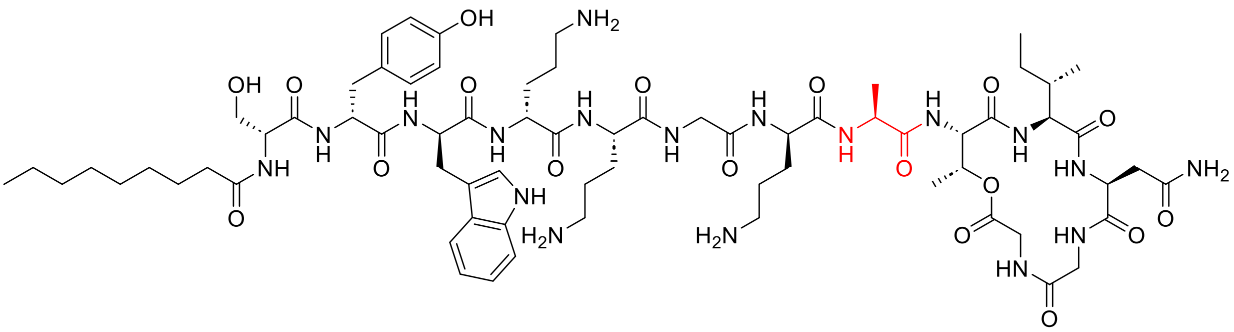

TFA salt was obtained in a yield of 2.7 mg (2.1 %), retention time (t_R) at 214 nm = 13.24 min (purity: 98.01 %). ESI-MS analysis of peak at 13.24 min: *m/z* (monoisotopic) $[M+H]^+$ 1491.00, $[M+2H]^{2+}$ 745.95, $[M+3H]^{3+}$ 497.70. Calculated mass (monoisotopic) for Peptide **28** ($C_{70}H_{108}N_{18}O_{18}$) 1489.74.

Peptide 29:

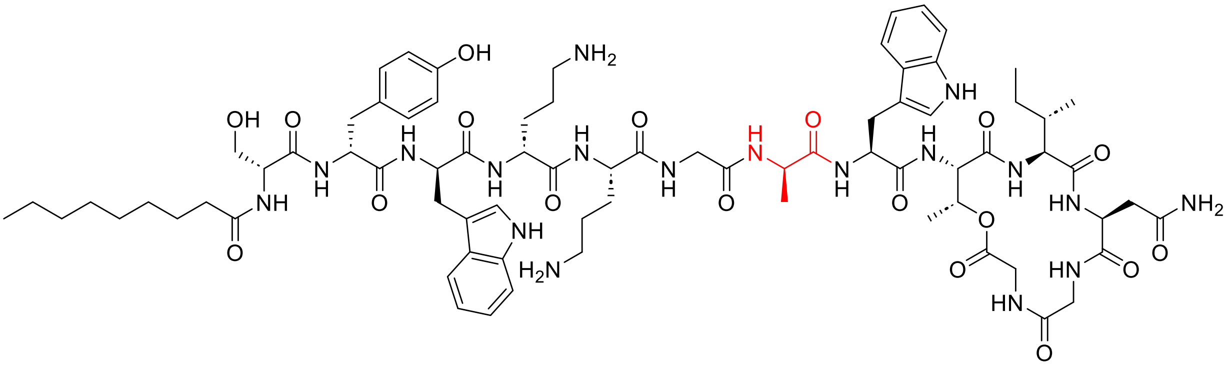

TFA salt was obtained in a yield of 4.8 mg (3.07 %), retention time (t_R) at 214 nm = 14.26 min (purity: 97.59 %). ESI-MS analysis of peak at 14.26 min: *m/z* (monoisotopic) $[M+H]^+$ 1563.20, $[M+2H]^{2+}$ 782.00. Calculated mass (monoisotopic) for Peptide **29** ($C_{76}H_{108}N_{18}O_{18}$) 1561.81.

Peptide 30:

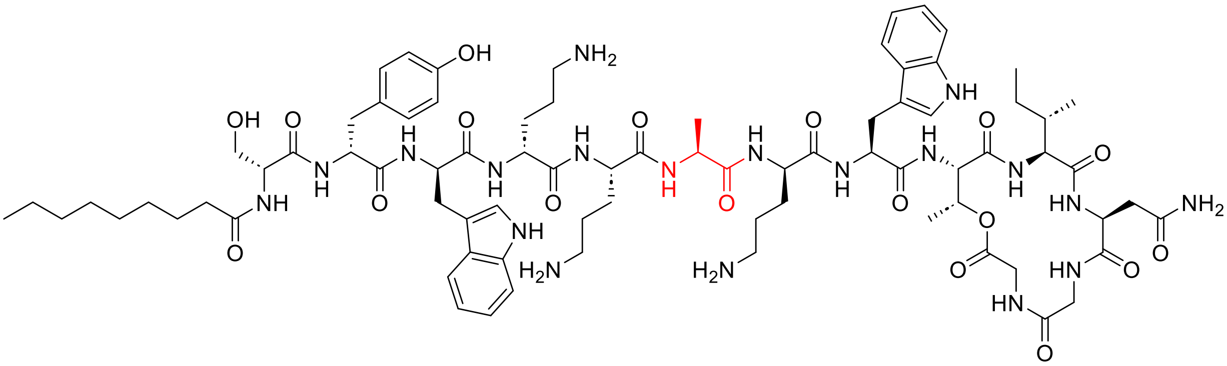

TFA salt was obtained in a yield of 7.0 mg (4.32 %), retention time (t_R) at 214 nm = 13.59 min (purity: 95.15 %). ESI-MS analysis of peak at 13.59 min: *m/z* (monoisotopic) $[M+H]^+$ 1620.30, $[M+2H]^{2+}$ 810.55, $[M+3H]^{3+}$ 540.75. Calculated mass (monoisotopic) for Peptide **30** ($C_{79}H_{115}N_{19}O_{18}$) 1618.90.

Peptide 31:

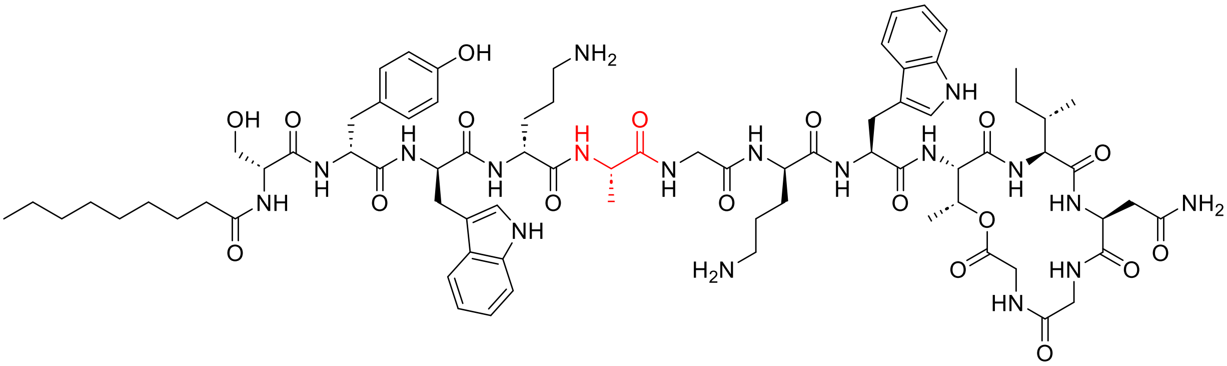

TFA salt was obtained in a yield of 6.4 mg (4.09 %), retention time (t_R) at 214 nm = 9.73 min (purity: 96.20 %). ESI-MS analysis of peak at 9.73 min: *m/z* (monoisotopic) $[M+H]^+$ 1563.25, $[M+2H]^{2+}$ 782.00. Calculated mass (monoisotopic) for Peptide **31** ($C_{76}H_{108}N_{18}O_{18}$) 1561.81.

Peptide 32:

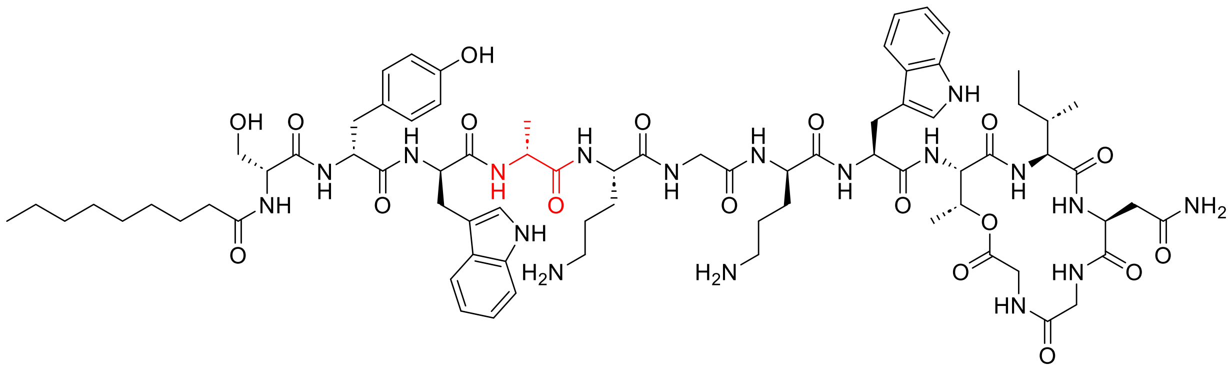

TFA salt was obtained in a yield of 4.9 mg (3.13 %), retention time (t_R) at 214 nm = 9.93 min (purity: 98.54 %). ESI-MS analysis of peak at 9.93 min: *m/z* (monoisotopic) $[M+H]^+$ 1563.25, $[M+2H]^{2+}$ 782.00. Calculated mass (monoisotopic) for Peptide **32** ($C_{76}H_{108}N_{18}O_{18}$) 1561.81.

Peptide 33:

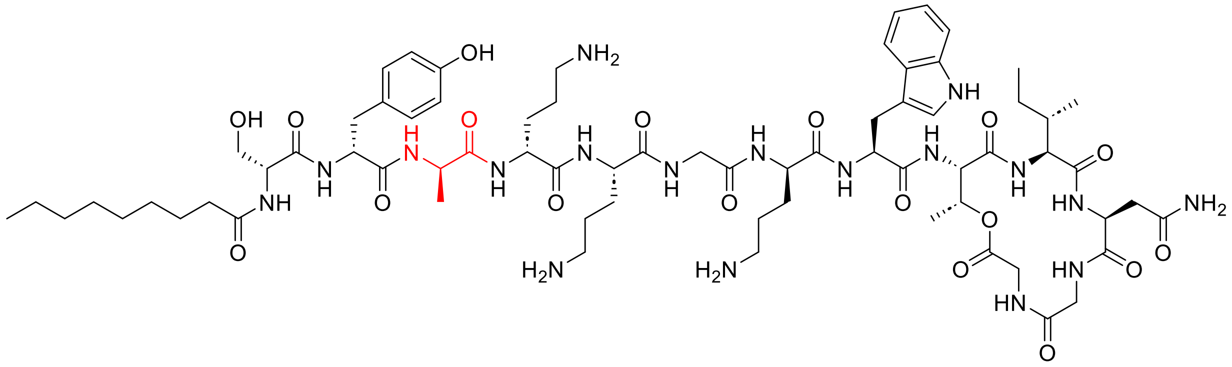

TFA salt was obtained in a yield of 1.2 mg (1.8 %), retention time (t_R) at 214 nm = 9.03 min (purity: 97.90 %). ESI-MS analysis of peak at 9.03 min: *m/z* (monoisotopic), $[M+2H]^{2+}$ 745.95, $[M+3H]^{3+}$ 497.70. Calculated mass (monoisotopic) for Peptide **33** ($C_{70}H_{108}N_{18}O_{18}$) 1489.74.

Peptide 34:

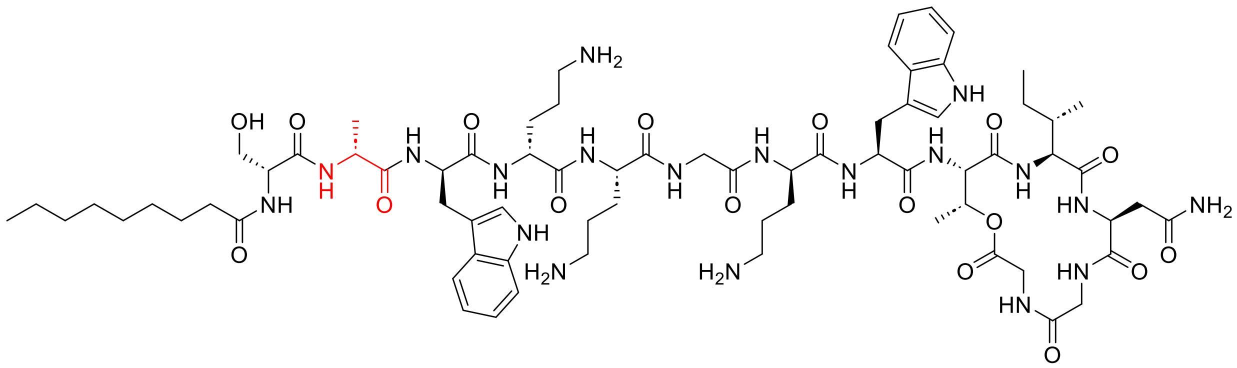

TFA salt was obtained in a yield of 5.4 mg (3.57 %), retention time (t_R) at 214 nm = 9.33 min (purity: 95.53 %). ESI-MS analysis of peak at 9.33 min: *m/z* (monoisotopic) $[M+H]^+$ 1513.25, $[M+2H]^{2+}$ 757.45, $[M+3H]^{3+}$ 505.40. Calculated mass (monoisotopic) for Peptide **34** ($C_{72}H_{109}N_{19}O_{17}$) 1512.78.

Peptide 35:

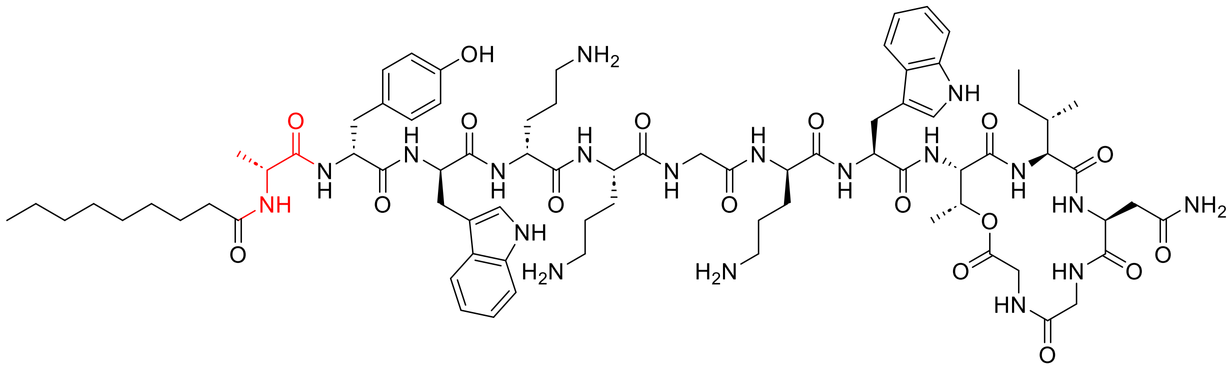

TFA salt was obtained in a yield of 5.4 mg (3.40 %), retention time (t_R) at 214 nm = 9.56 min (purity: 97.24 %). ESI-MS analysis of peak at 9.56 min: *m/z* (monoisotopic) $[M+H]^+$ 1590.30, $[M+2H]^{2+}$ 795.60, $[M+3H]^{3+}$ 530.75. Calculated mass (monoisotopic) for Peptide **35** ($C_{78}H_{113}N_{19}O_{17}$) 1588.88.

HPLC and MS Data

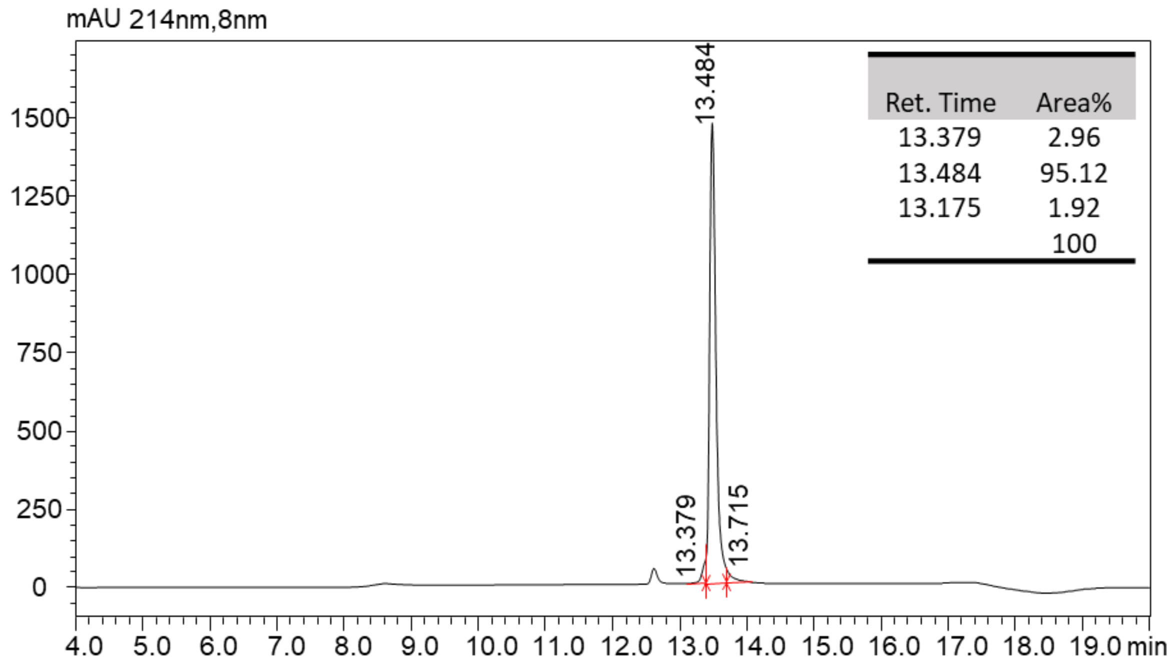

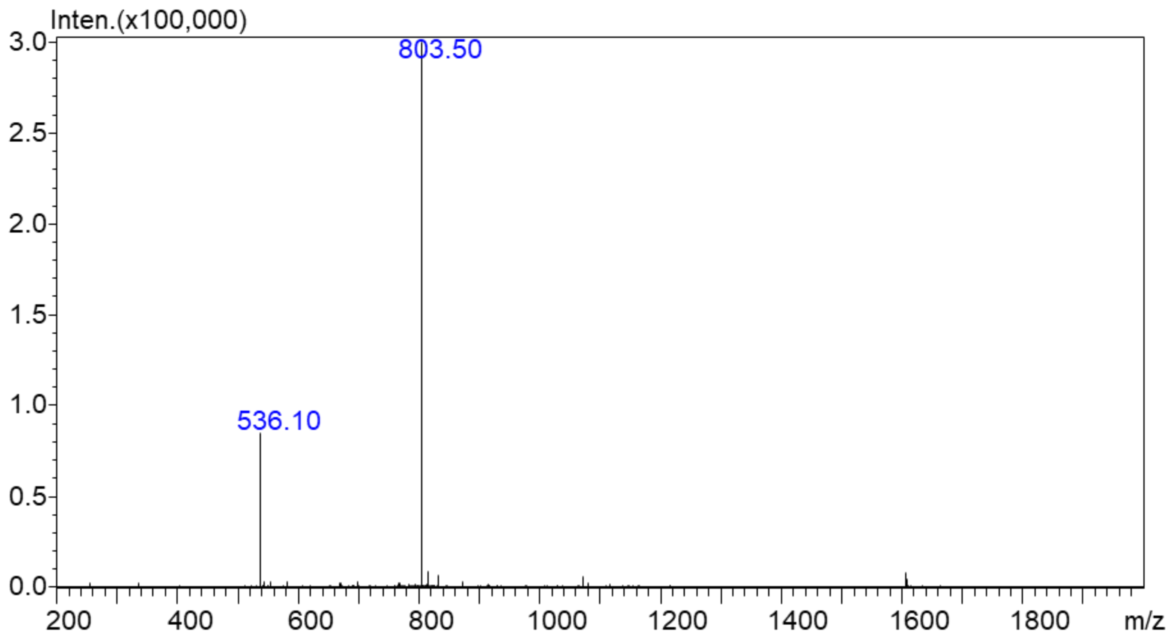

Figure S4. LC-MS Profile for Laterocidine Peptide **1**. (A) LC profile at 214 nm. (B) MS spectrum of the peak at 13.48 min.

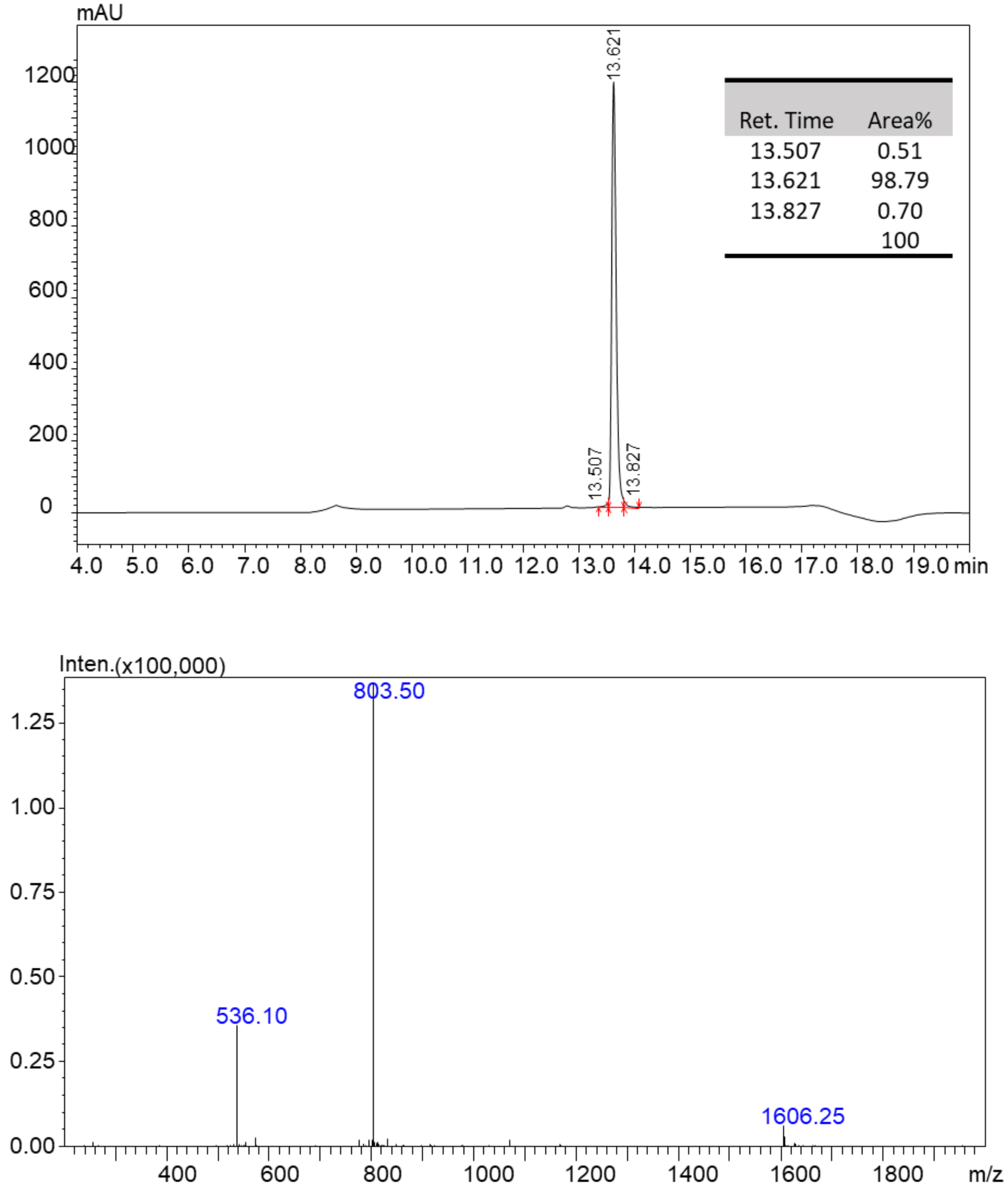

Figure S5. LC-MS Profile for Peptide **11**. (A) LC profile at 214 nm. (B) MS spectrum of the peak at 13.62 min.

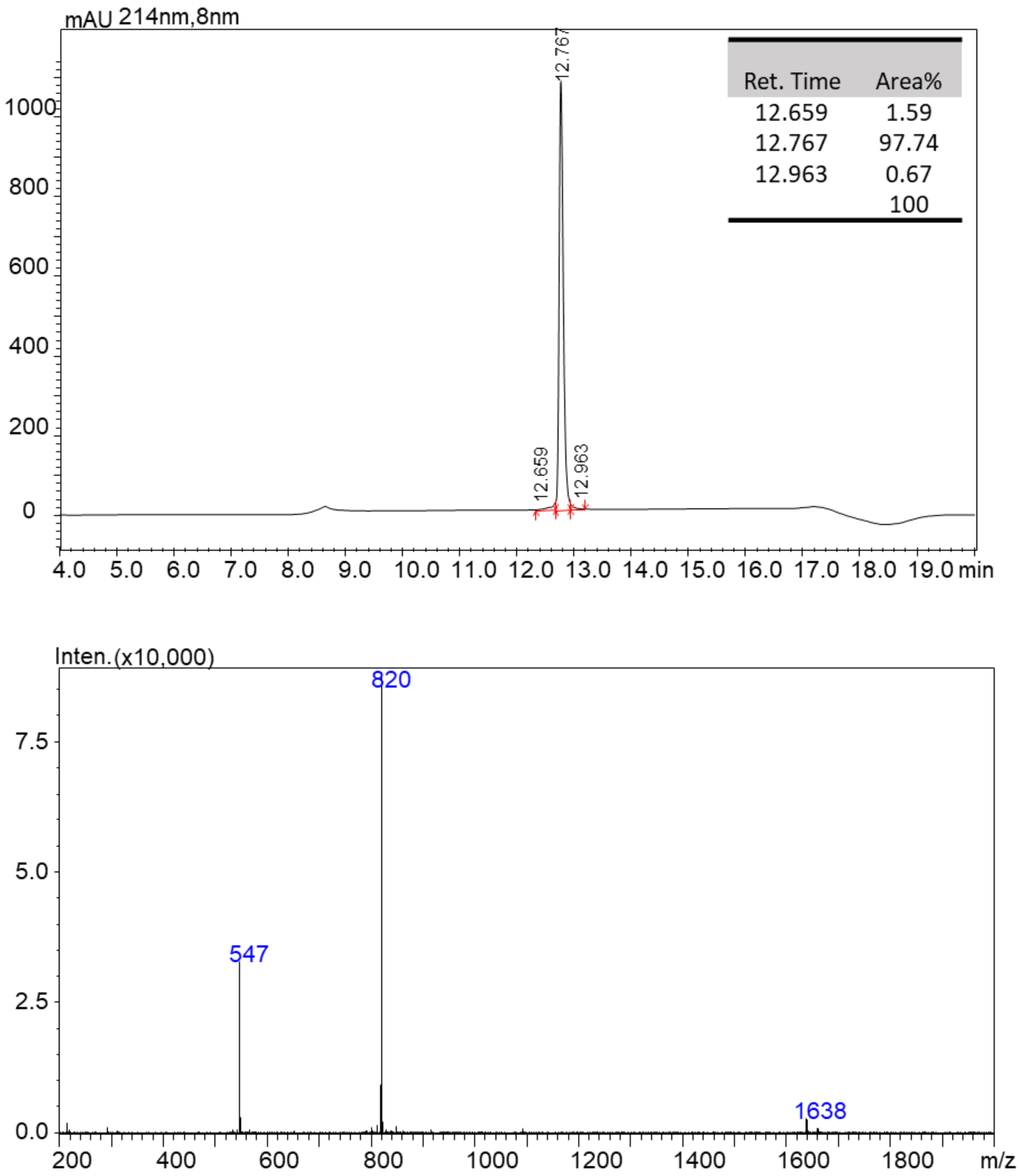

Figure S6. LC-MS Profile for Peptide **12**. (A) LC profile at 214 nm. (B) MS spectrum of the peak at 12.76 min.

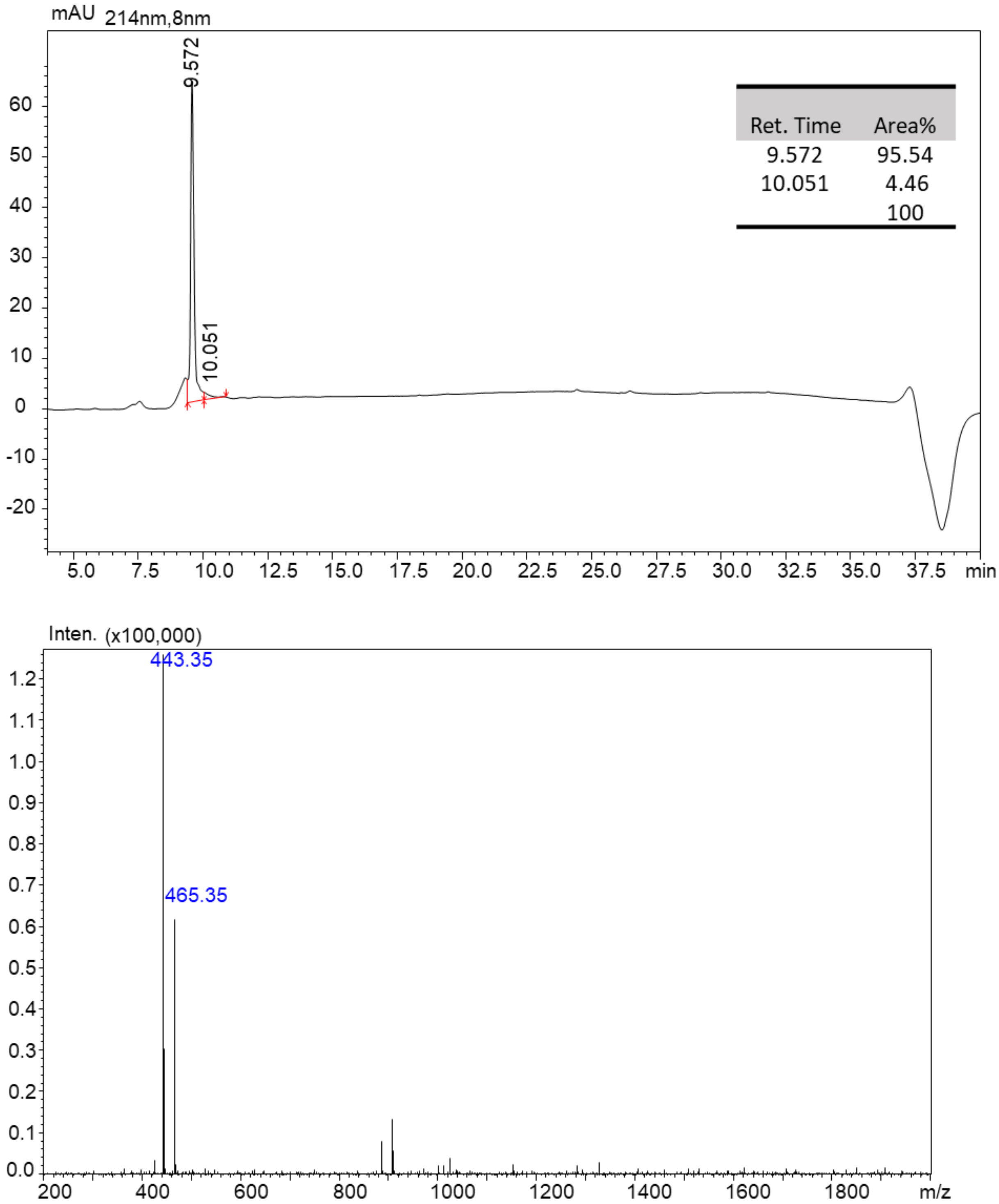

Figure S7. LC-MS Profile for Peptide **13**. (A) LC profile at 214 nm. (B) MS spectrum of the peak at 9.57 min.

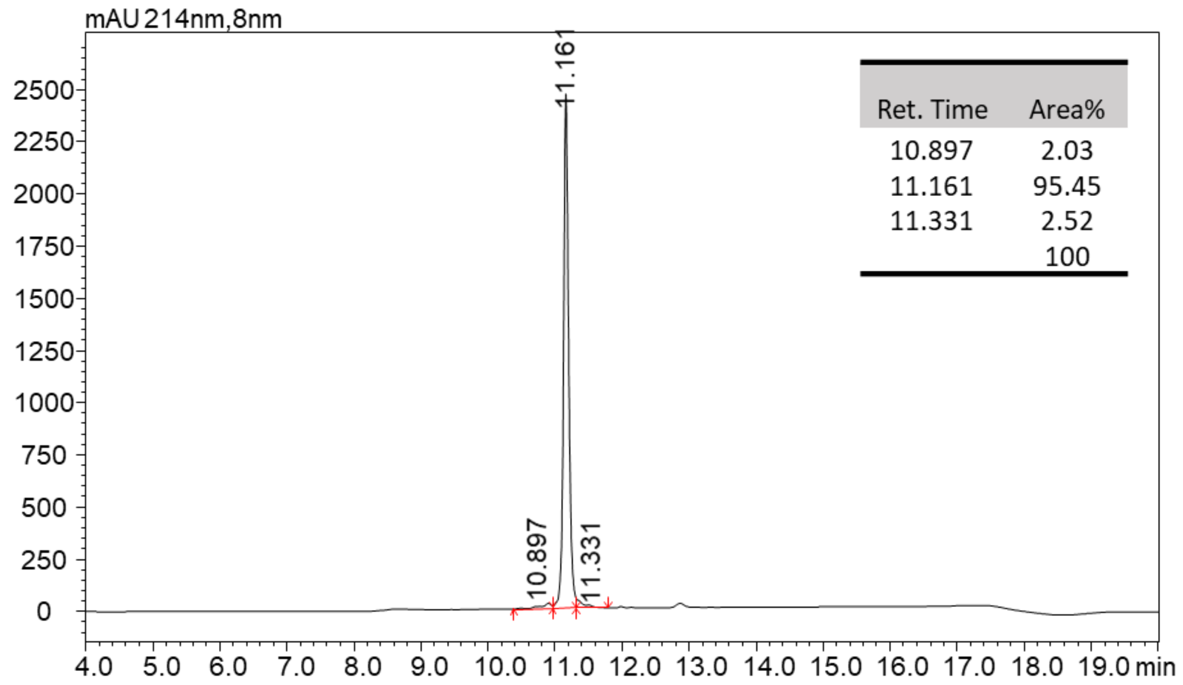

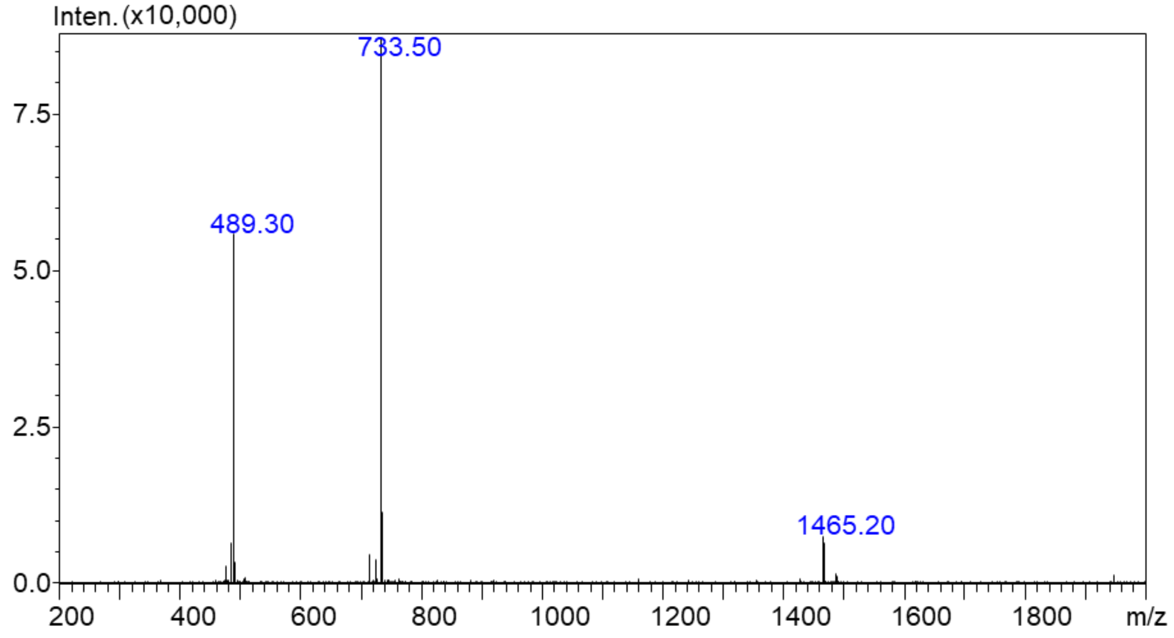

Figure S8. LC-MS Profile for peptide **14**. (A) LC profile at 214 nm. (B) MS spectrum of the peak at 11.16 min.

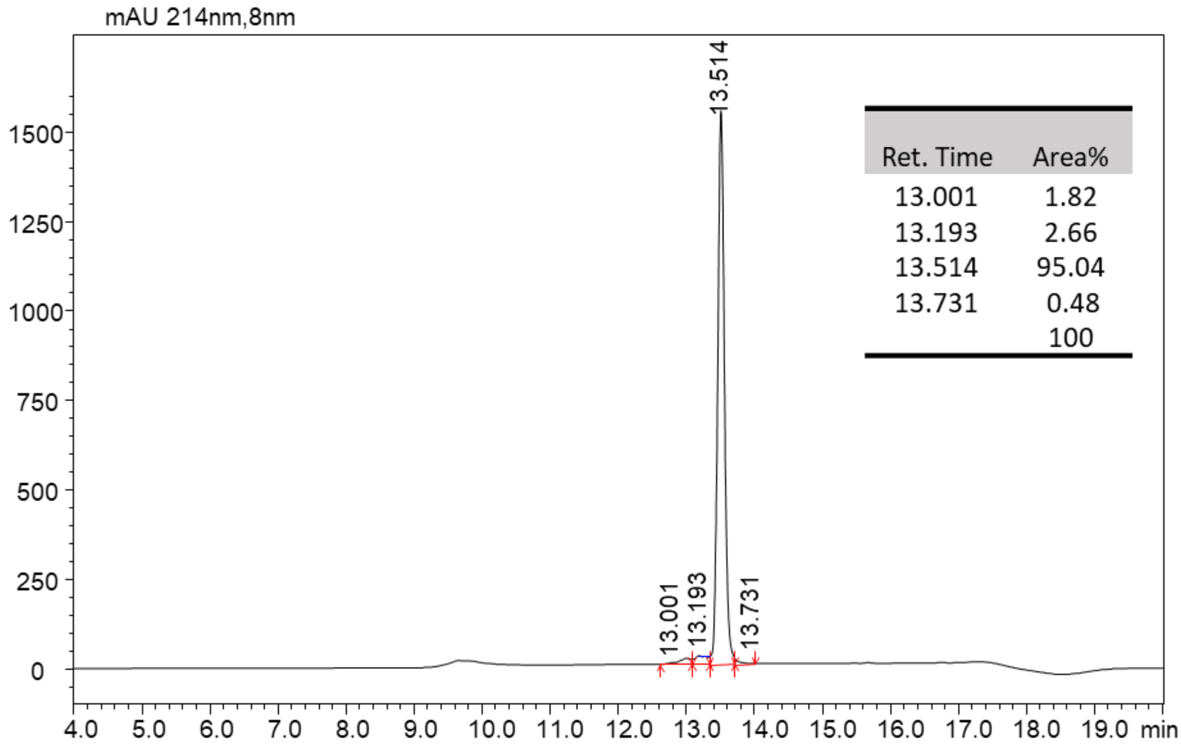

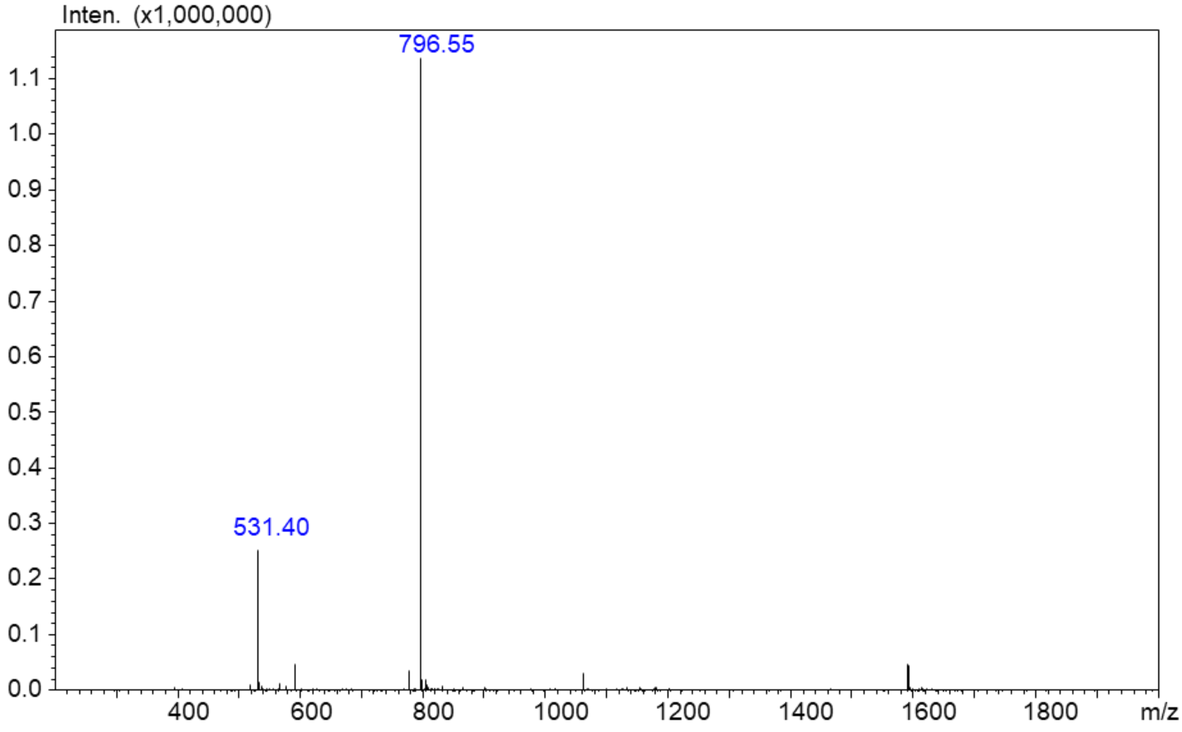

Figure S9. LC-MS Profile for peptide **15**. (A) LC profile at 214 nm. (B) MS spectrum of the peak at 13.52 min.

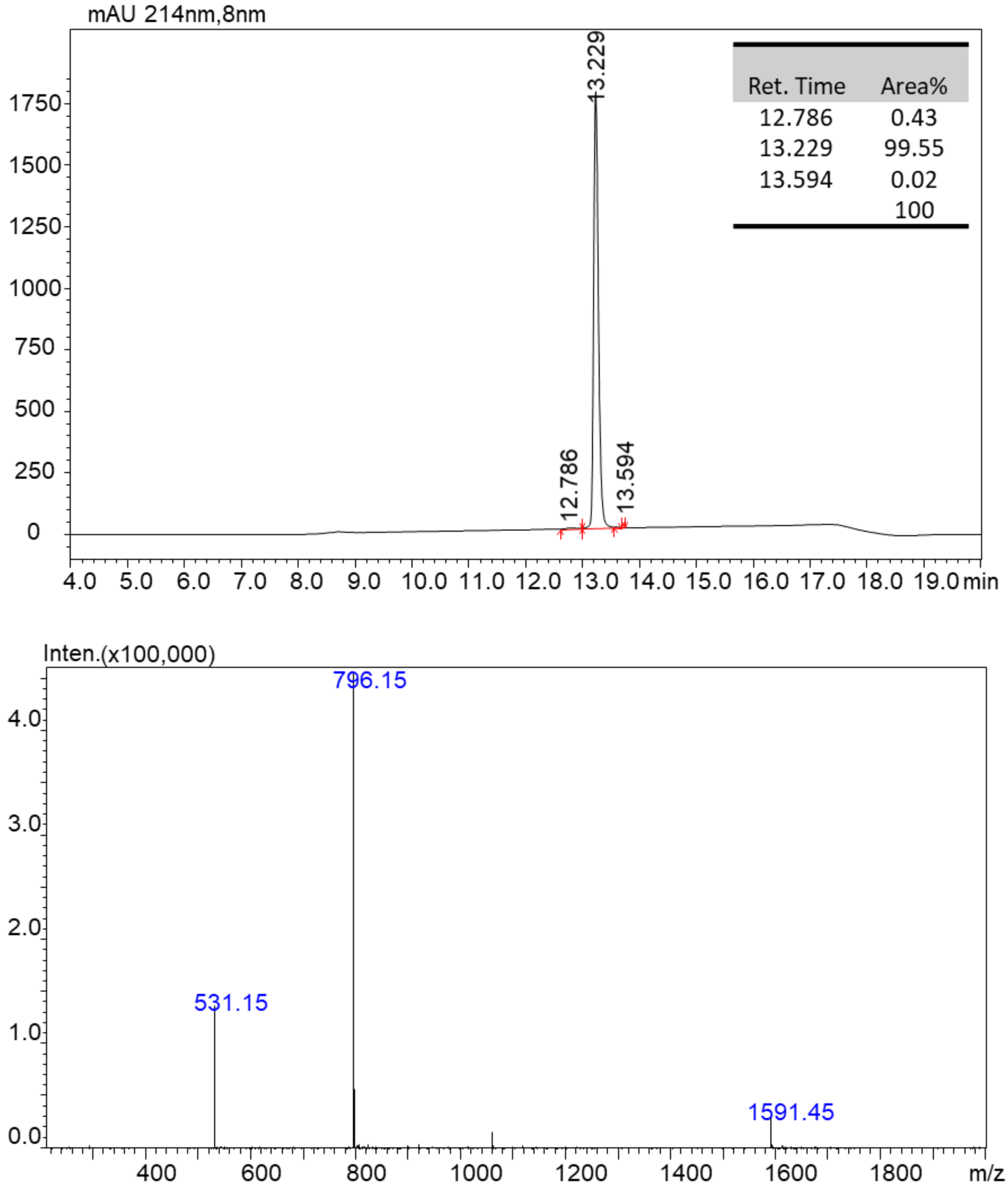

Figure S10. LC-MS Profile for peptide **16**. (A) LC profile at 214 nm. (B) MS spectrum of the peak at 13.23 min.

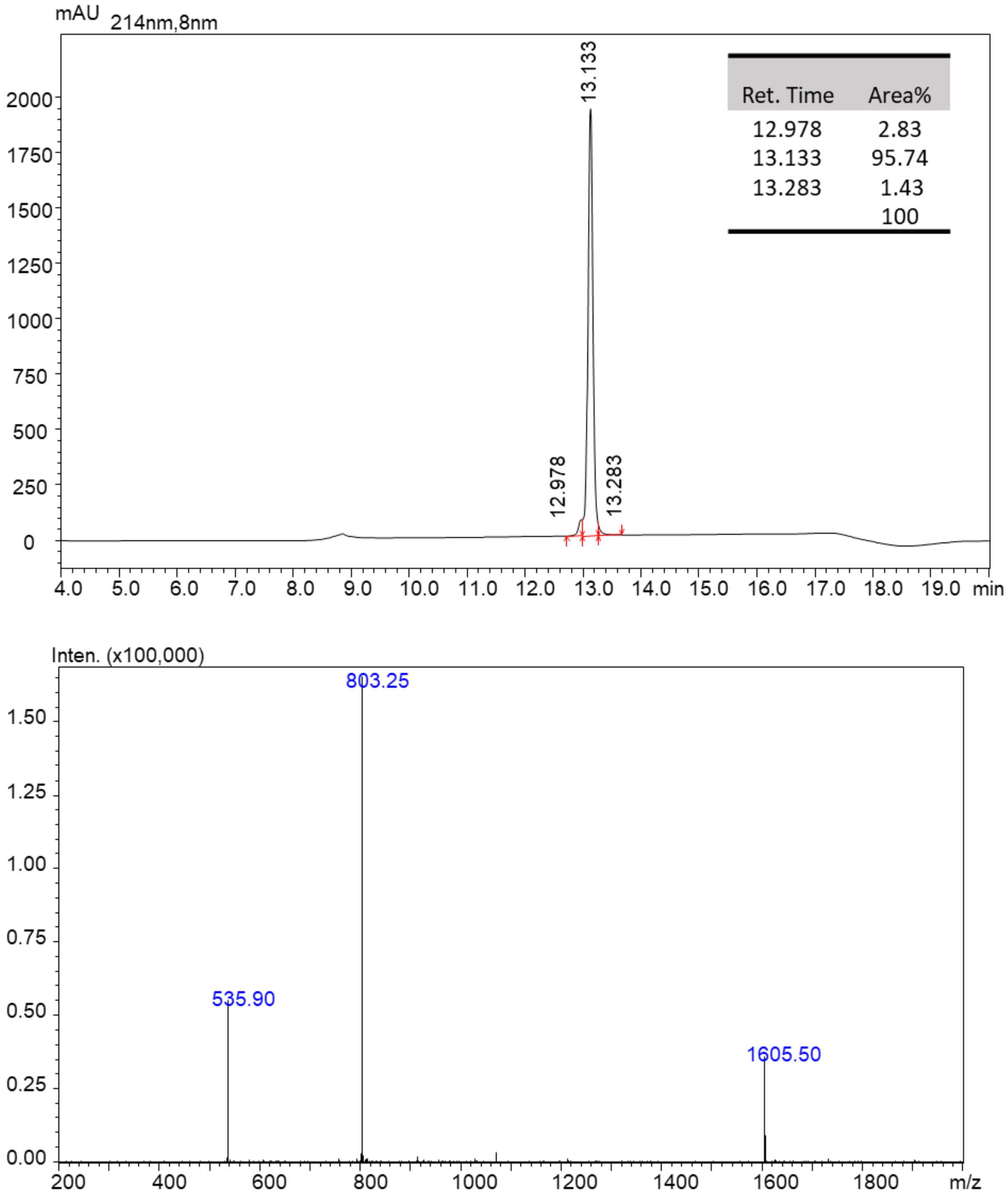

Figure S11. LC-MS Profile for peptide **17**. (A) LC profile at 214 nm. (B) MS spectrum of the peak at 13.13 min.

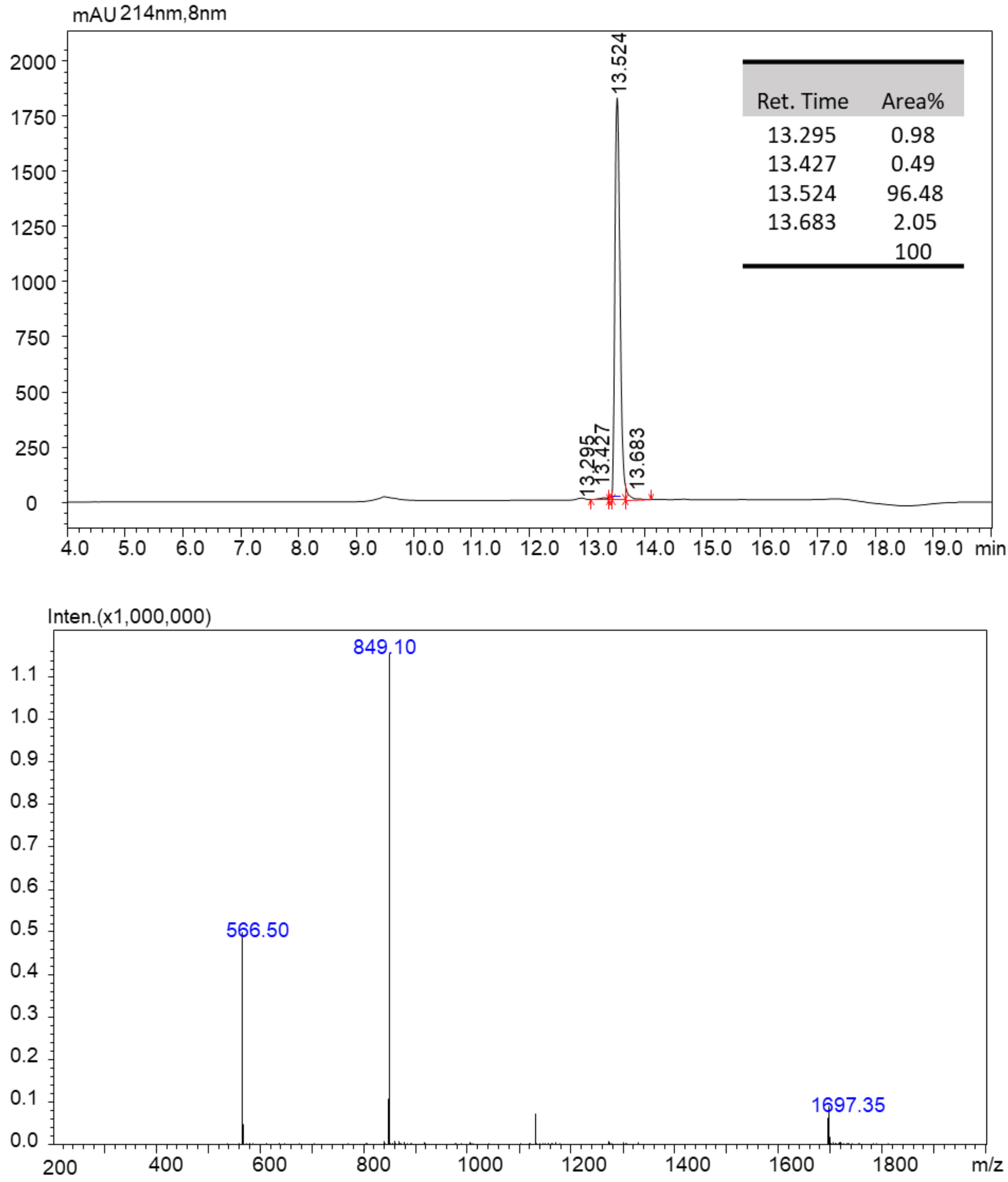

Figure S12. LC-MS Profile for peptide **18**. (A) LC profile at 214 nm. (B) MS spectrum of the peak at 13.52 min.

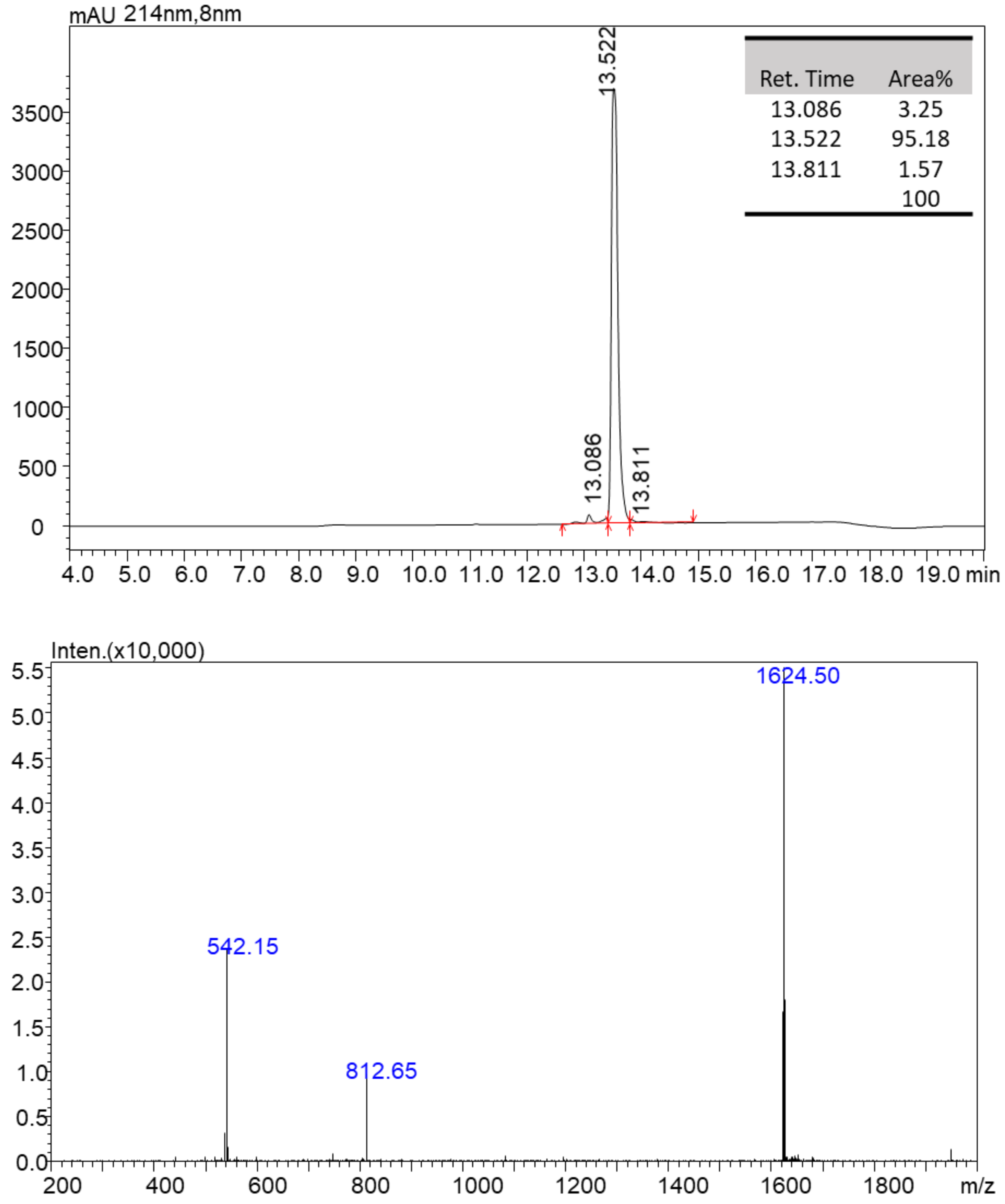

Figure S13. LC-MS Profile for peptide **19**. (A) LC profile at 214 nm. (B) MS spectrum of the peak at 13.52 min.

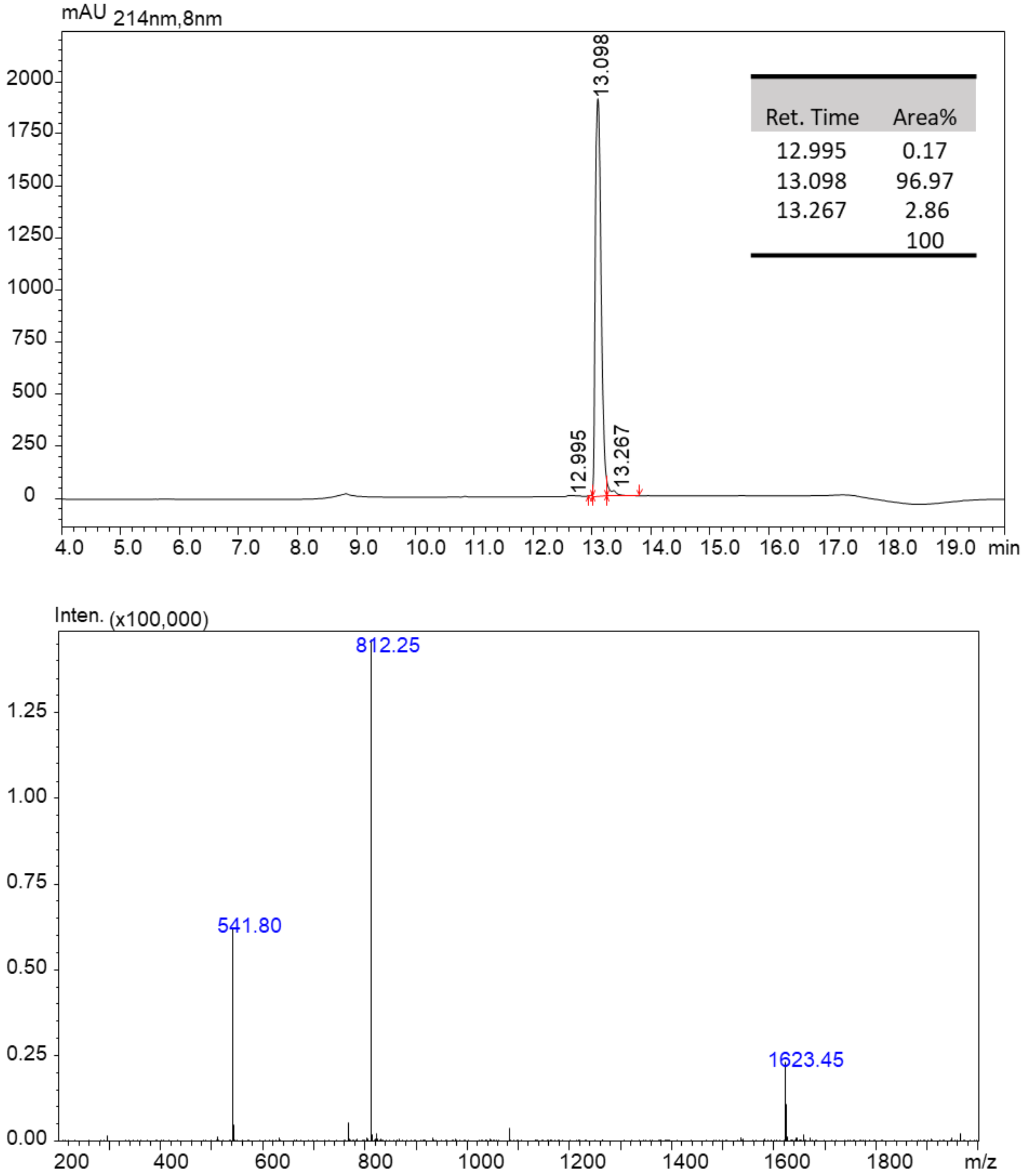

Figure S14. LC-MS Profile for peptide **20**. (A) LC profile at 214 nm. (B) MS spectrum of the peak at 13.09 min.

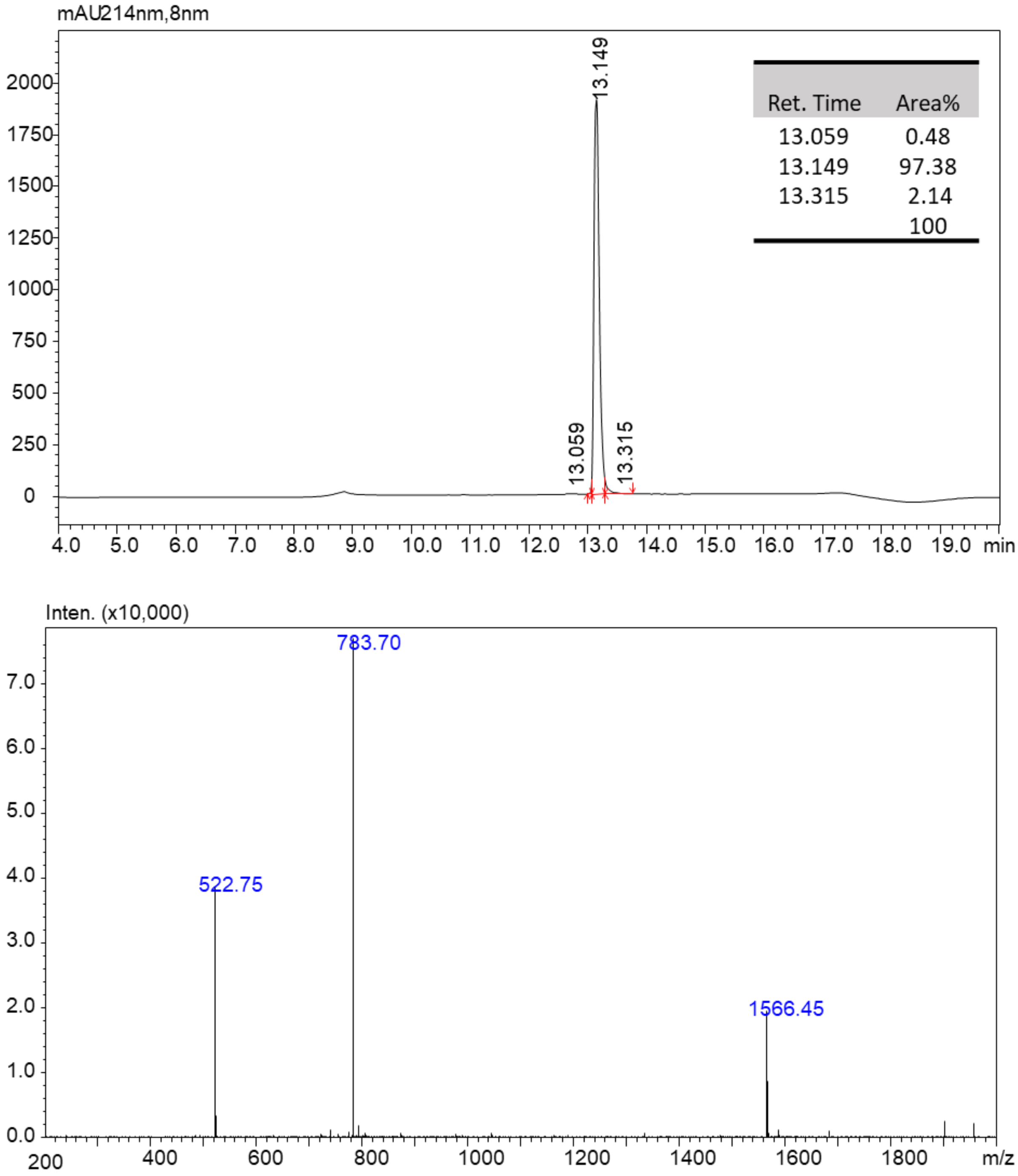

Figure S15. LC-MS Profile for peptide **21**. (A) LC profile at 214 nm. (B) MS spectrum of the peak at 13.14 min.

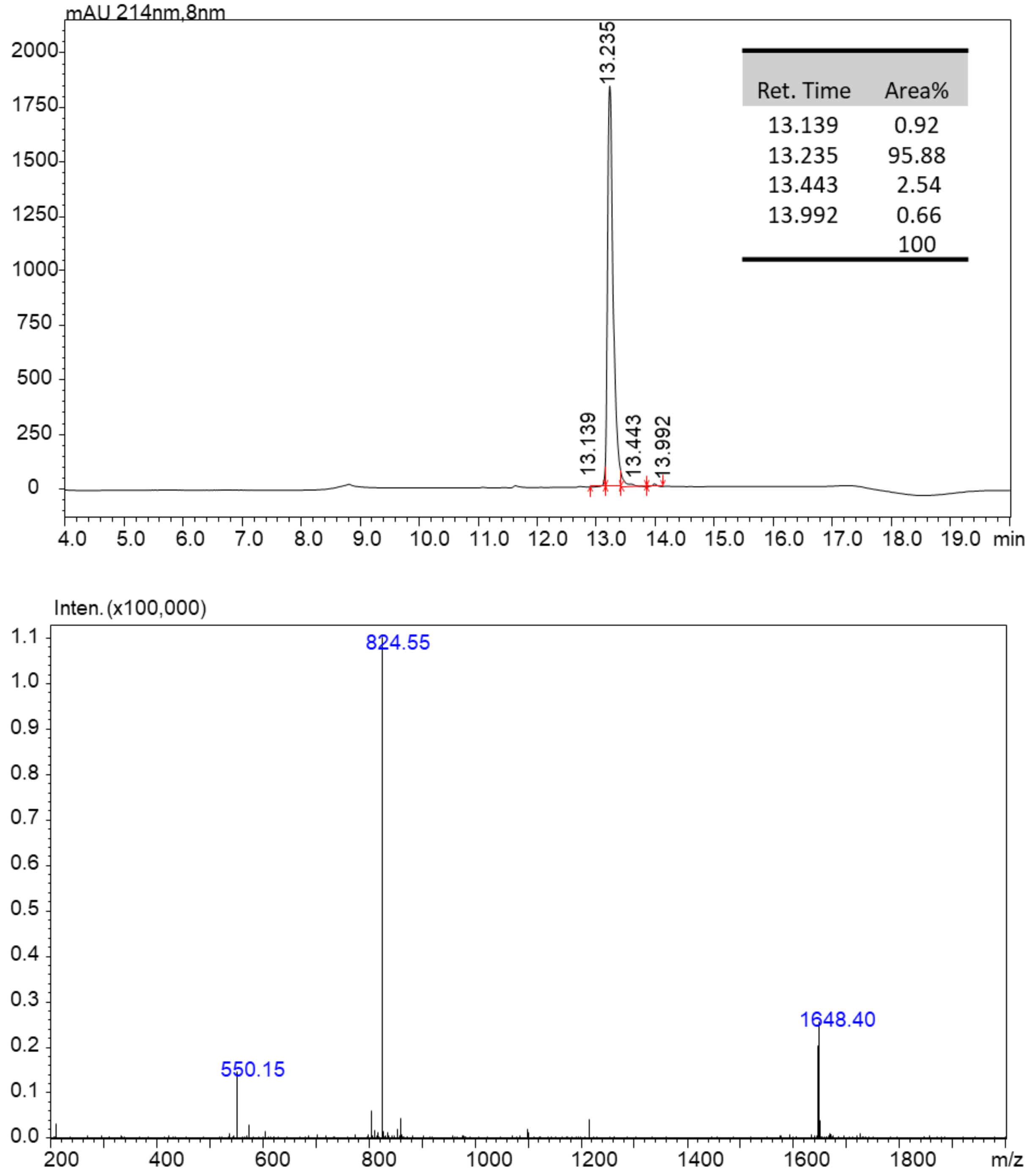

Ret. Time	Area%
13.139	0.92
13.235	95.88
13.443	2.54
13.992	0.66
	100

Figure S16. LC-MS Profile for peptide **22**. (A) LC profile at 214 nm. (B) MS spectrum of the peak at 13.23 min.

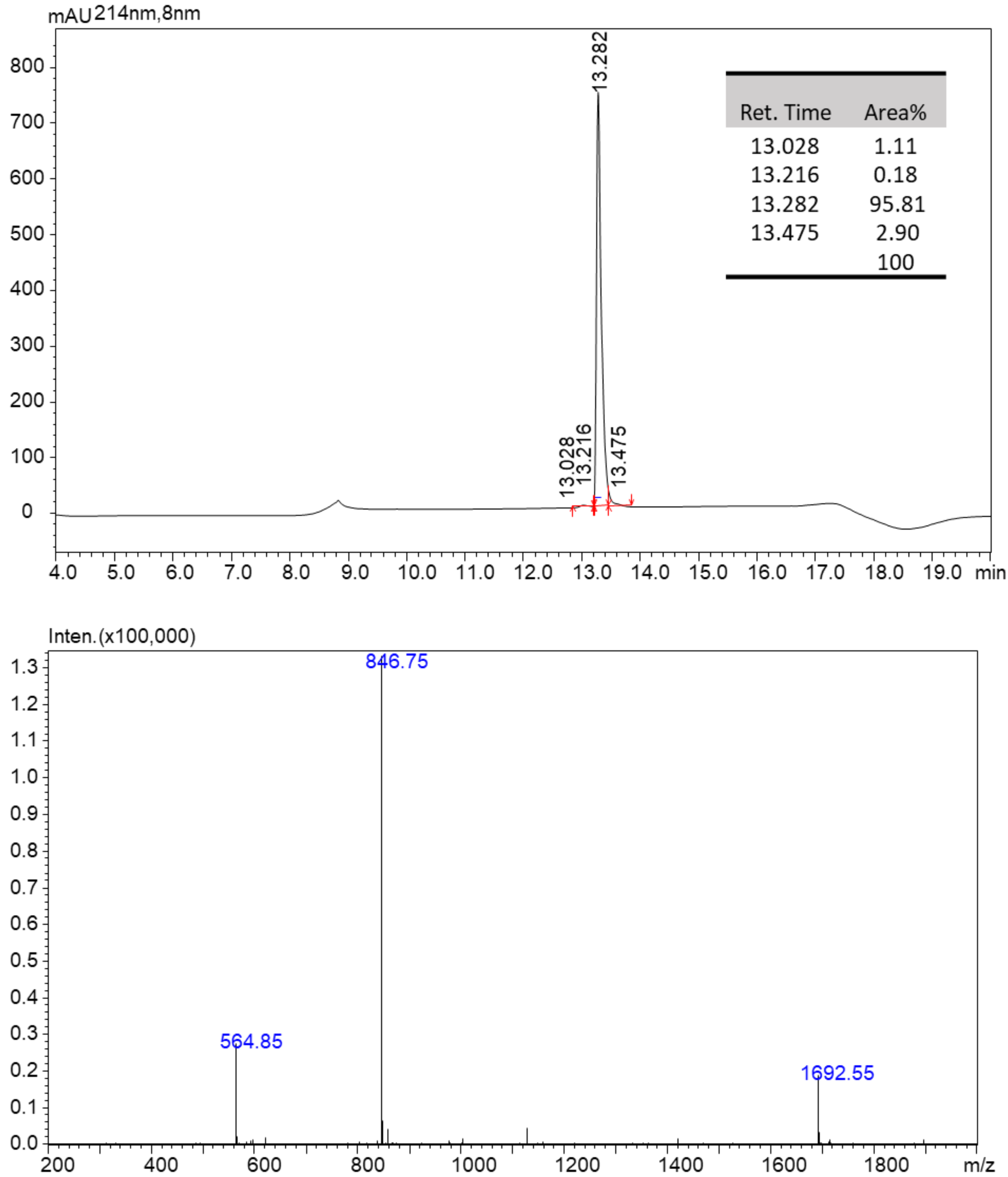

Figure S17. LC-MS Profile for peptide **23**. (A) LC profile at 214 nm. (B) MS spectrum of the peak at 13.28 min.

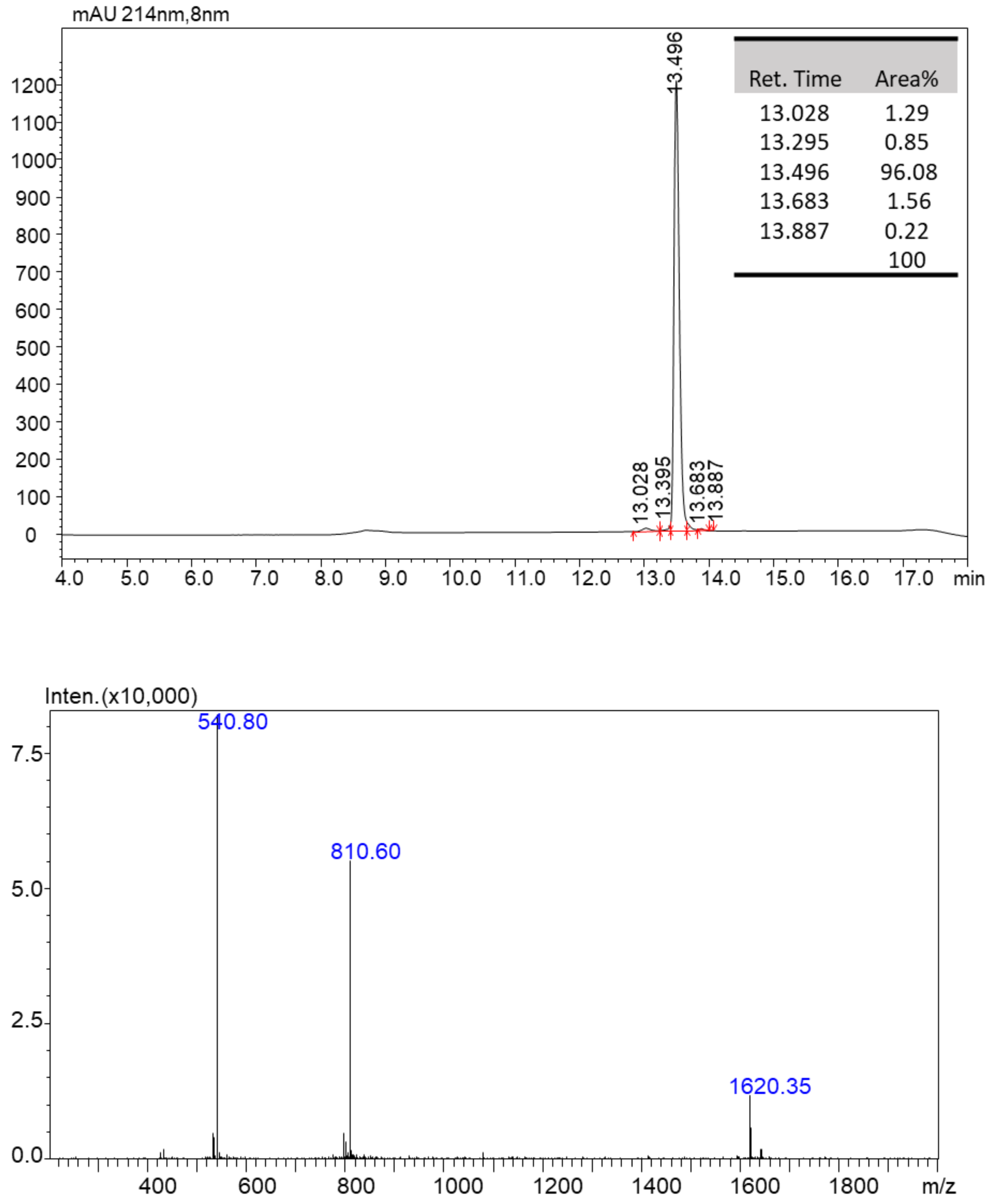

Figure S18. LC-MS Profile for peptide **24**. (A) LC profile at 214 nm. (B) MS spectrum of the peak at 10.75 min.

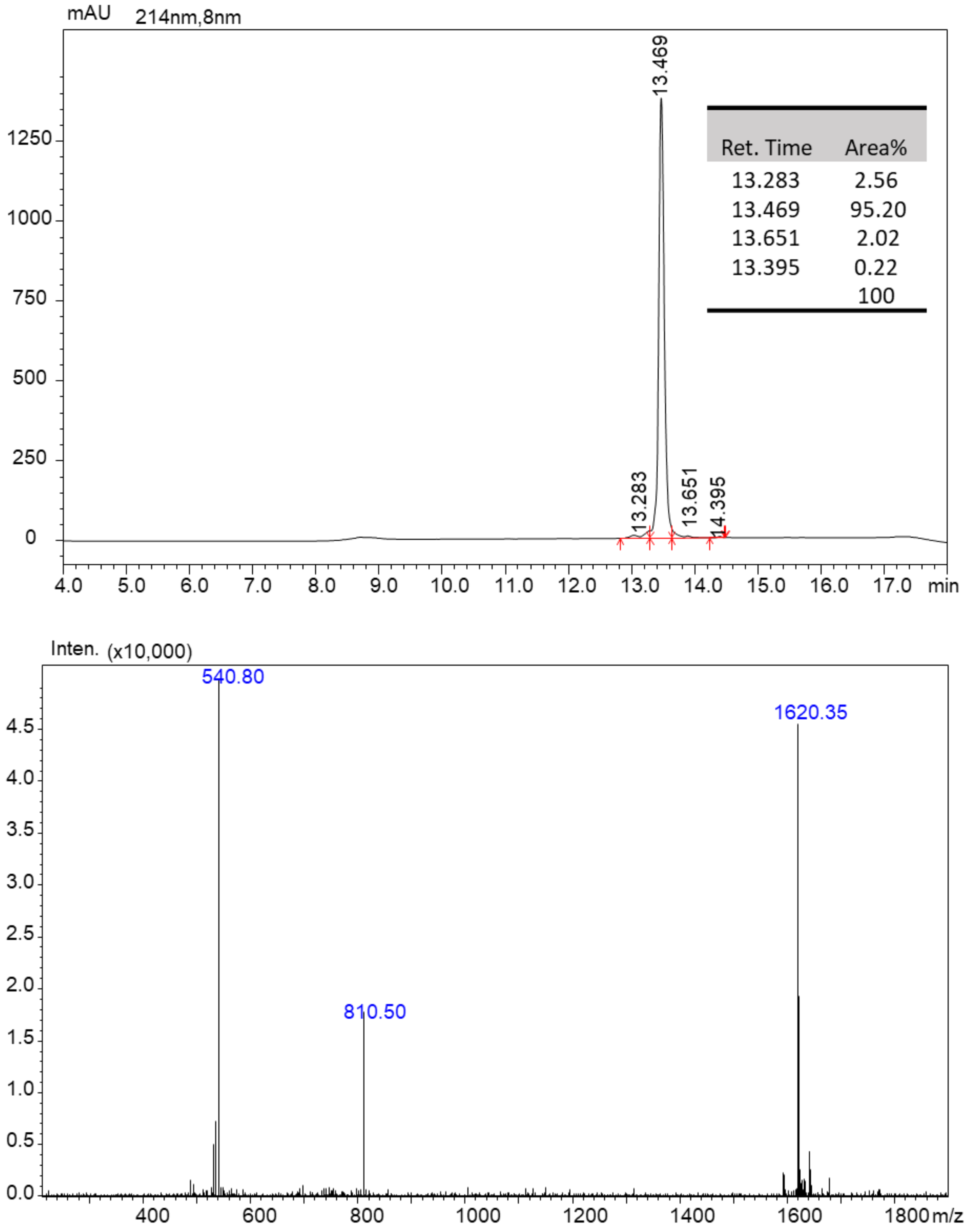

Figure S19. LC-MS Profile for peptide **25**. (A) LC profile at 214 nm. (B) MS spectrum of the peak at 10.71 min.

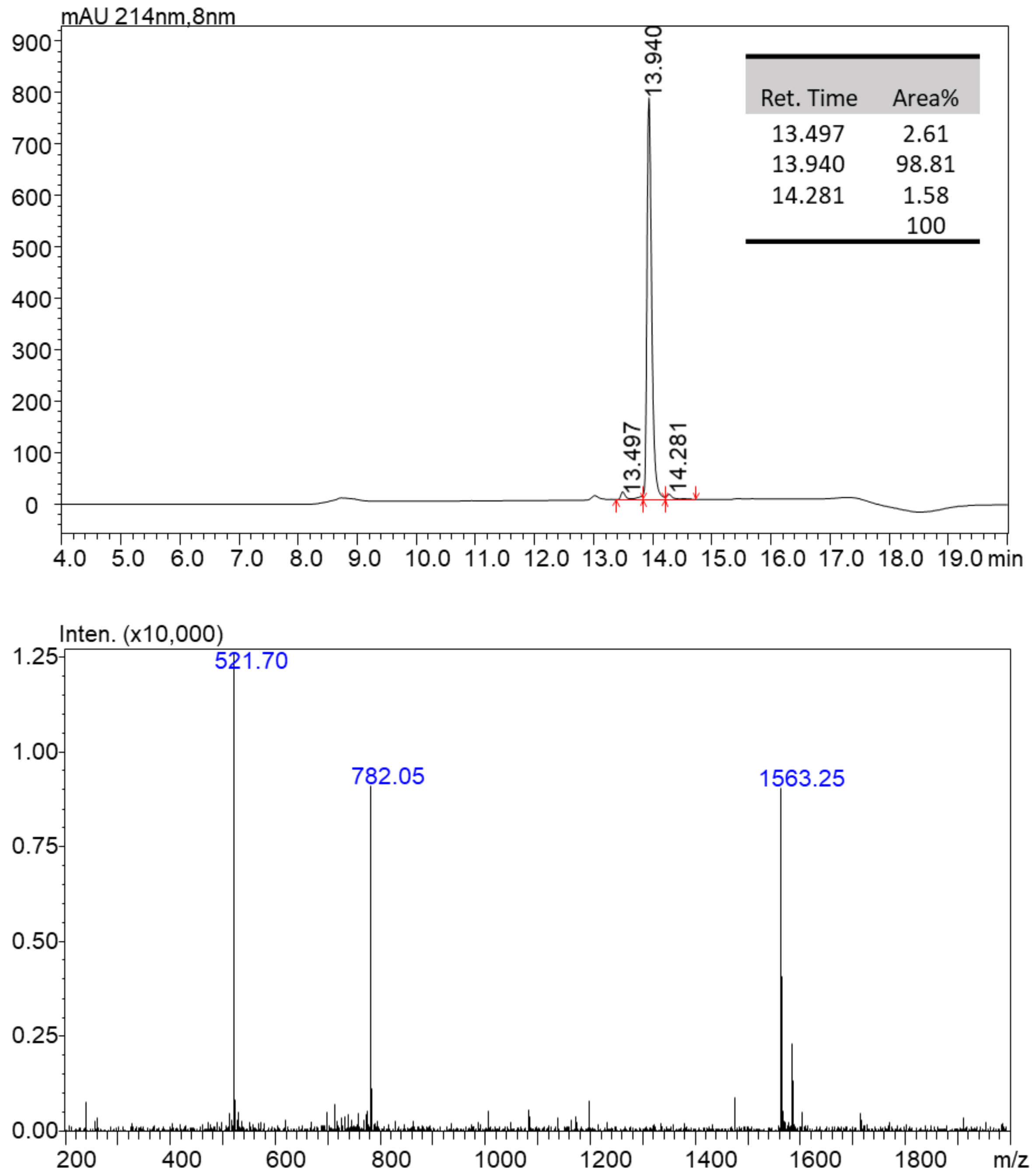

Figure S20. LC-MS Profile for peptide **26**. (A) LC profile at 214 nm. (B) MS spectrum of the peak at 13.94 min.

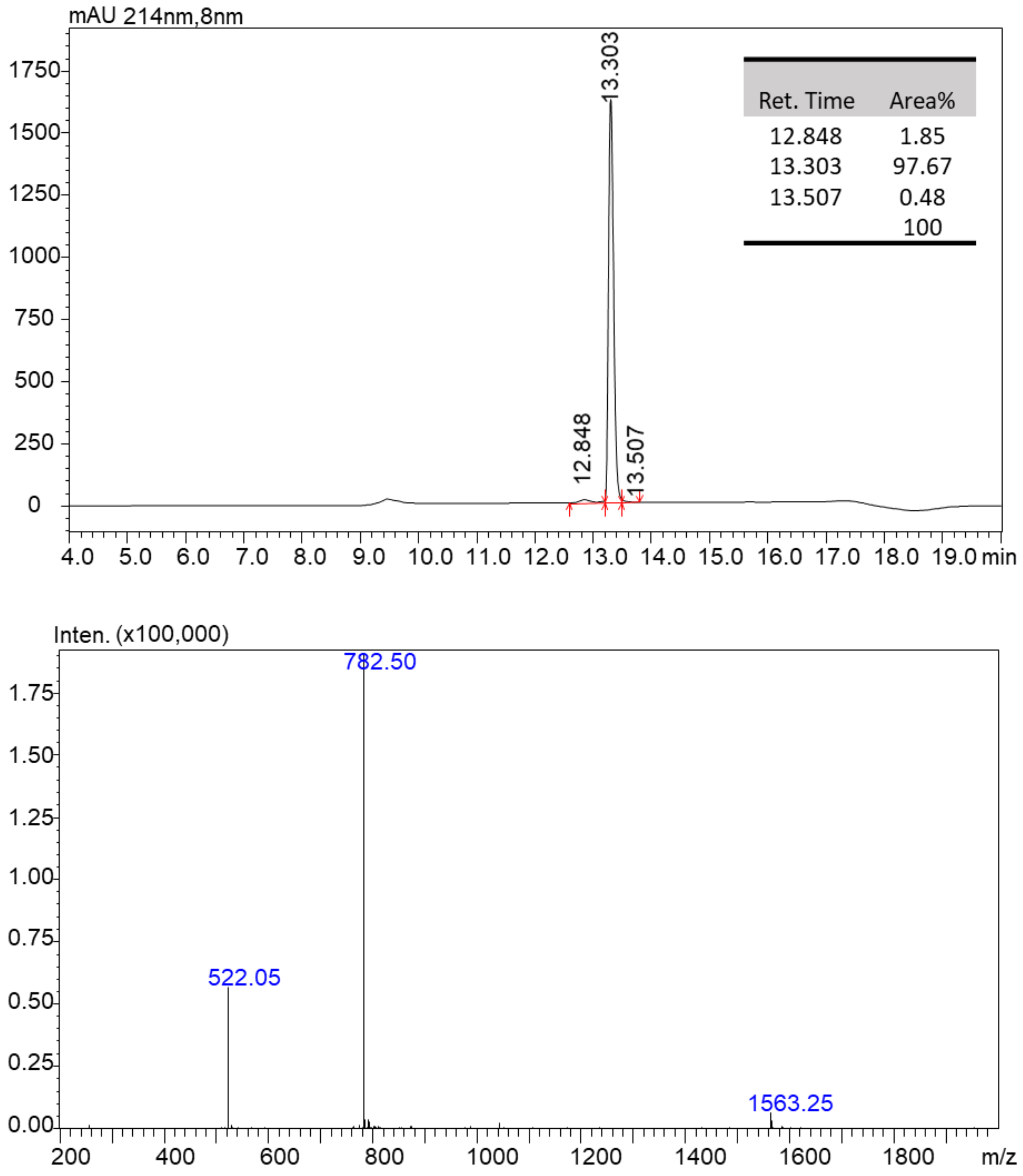

Figure S21. LC-MS Profile for peptide **27**. (A) LC profile at 214 nm. (B) MS spectrum of the peak at 13.30 min.

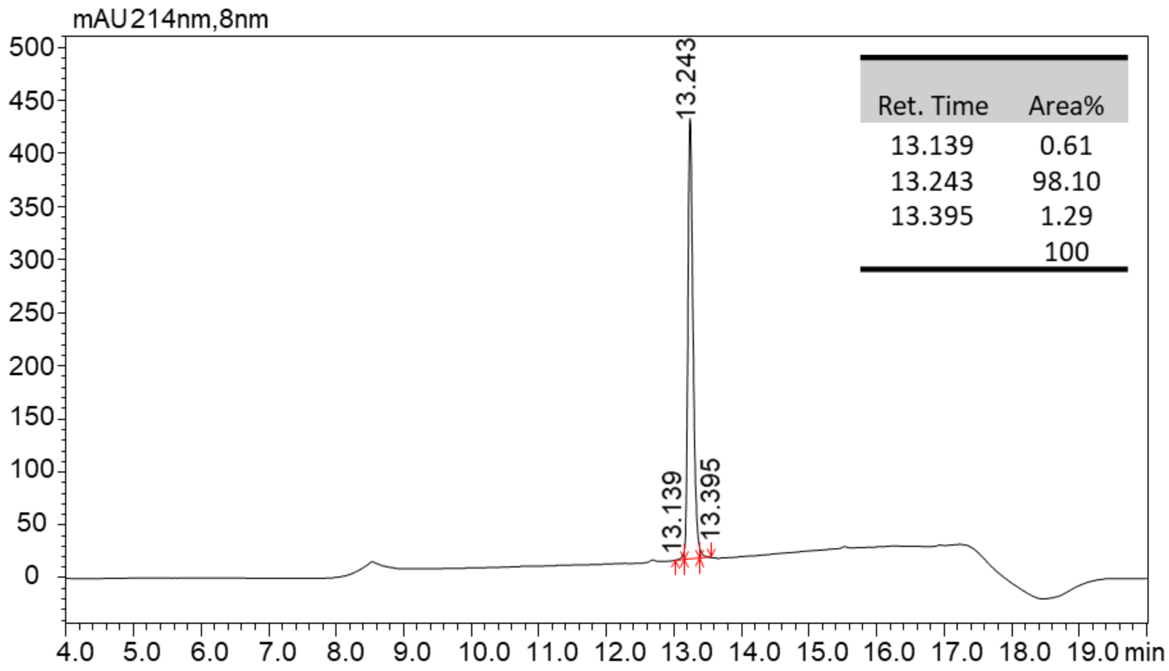

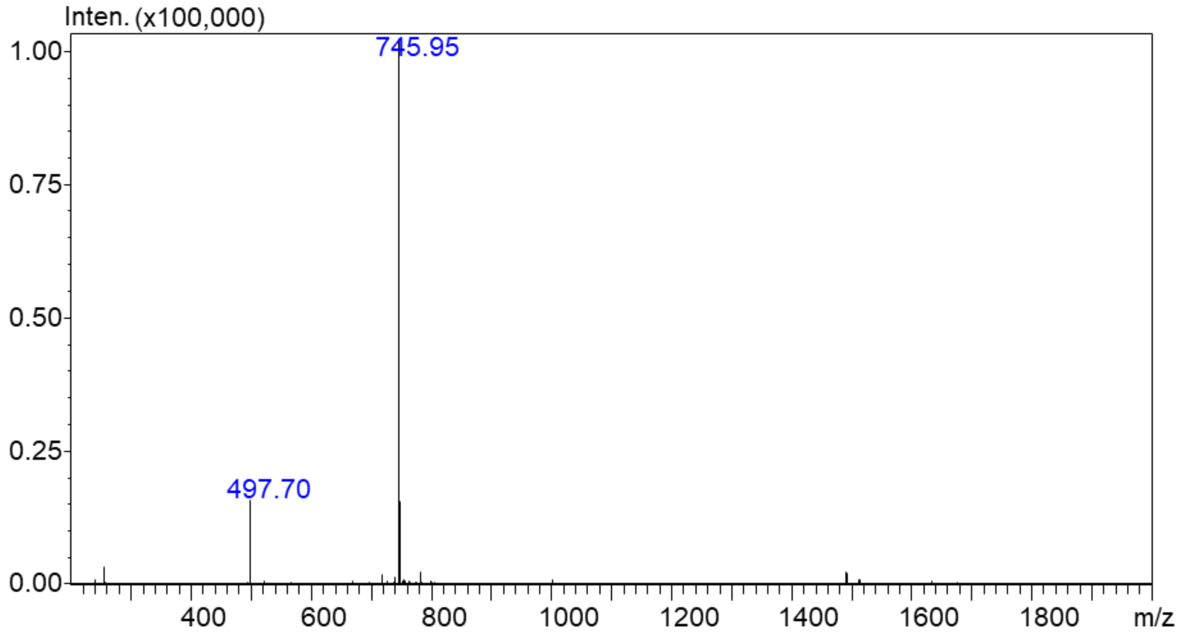

Figure S22. LC-MS Profile for peptide **28**. (A) LC profile at 214 nm. (B) MS spectrum of the peak at 13.24 min.

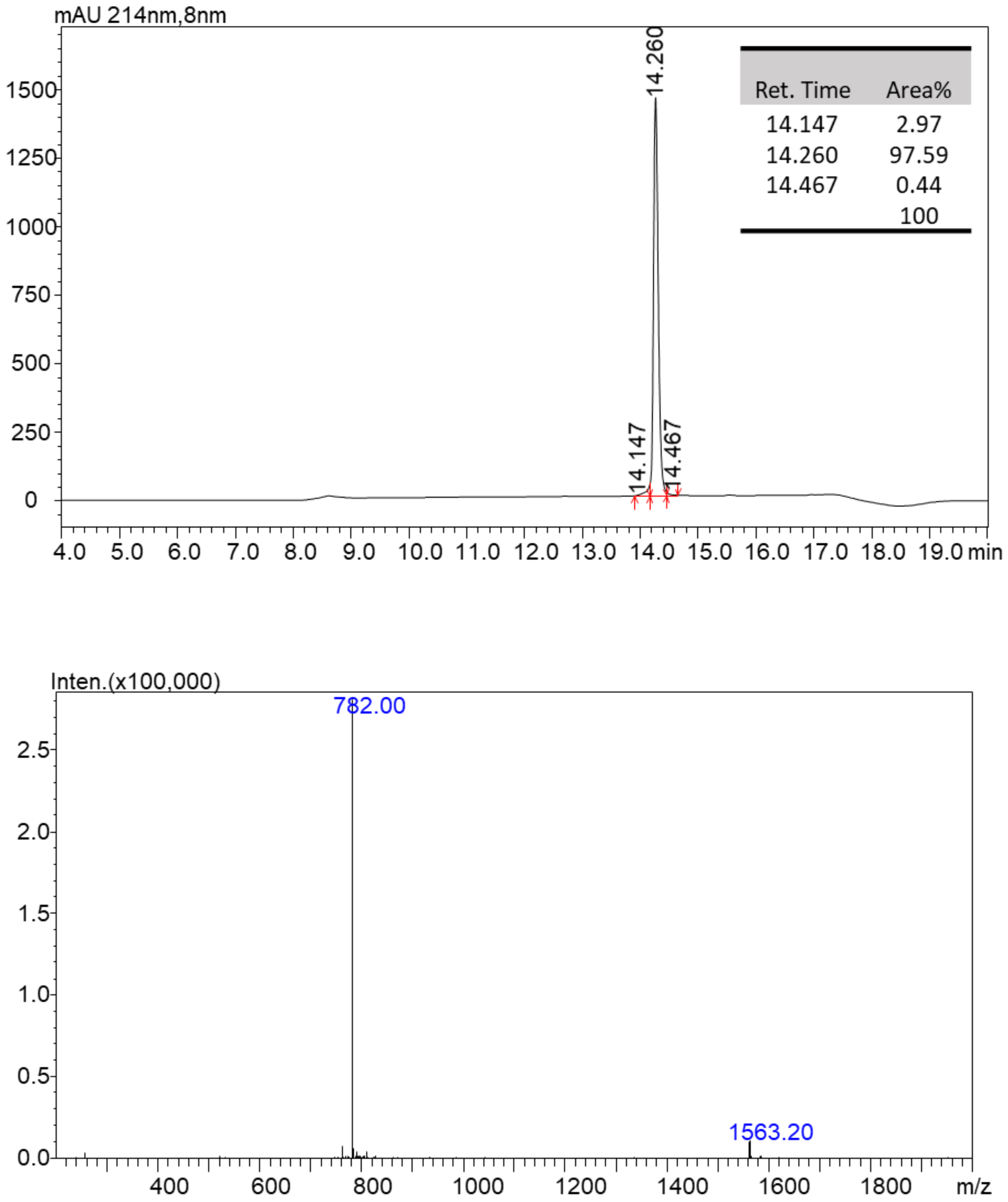

Figure S23. LC-MS Profile for peptide **29**. (A) LC profile at 214 nm. (B) MS spectrum of the peak at 14.26 min.

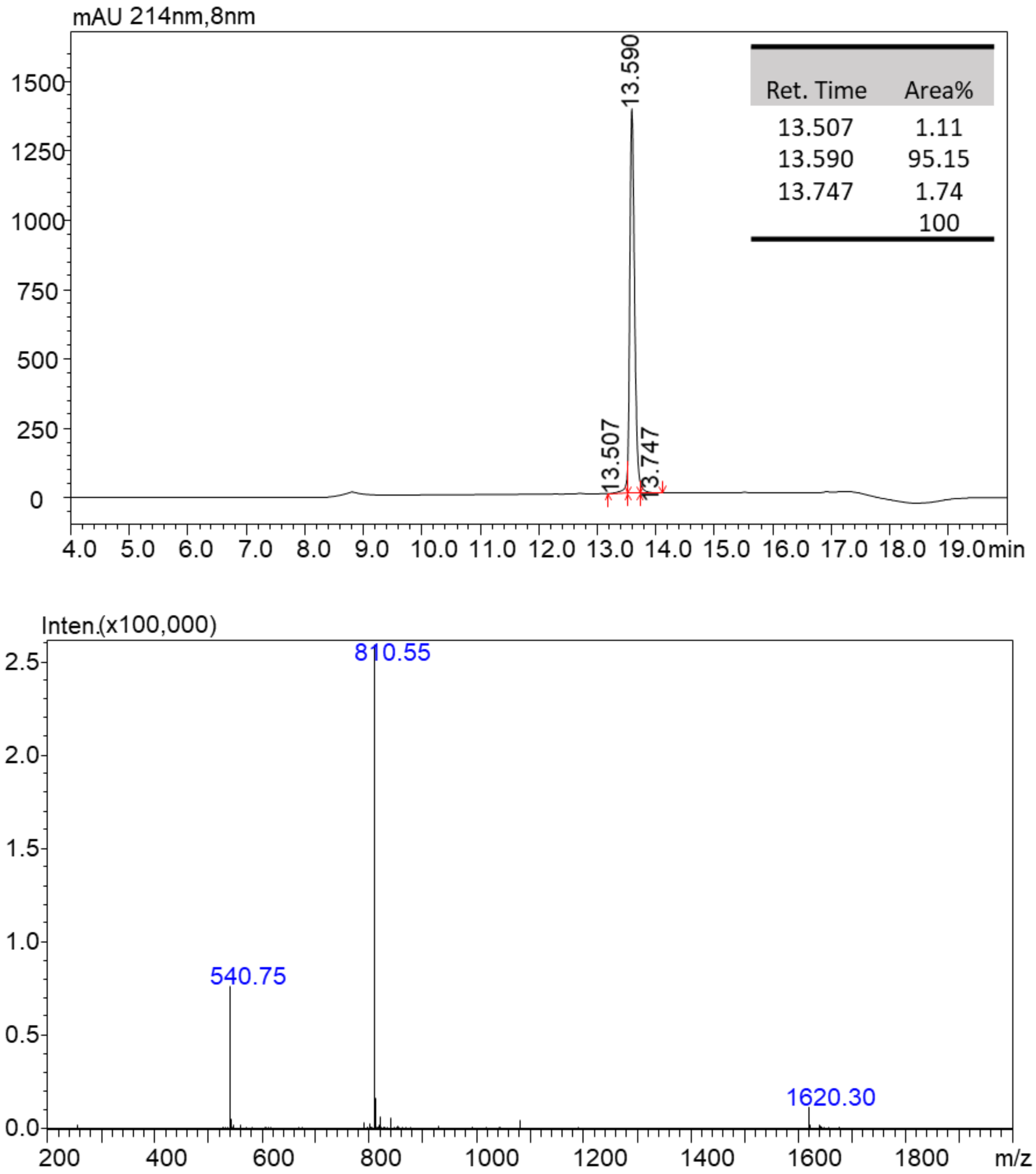

Figure S24. LC-MS Profile for peptide **30**. (A) LC profile at 214 nm. (B) MS spectrum of the peak at 13.59 min.

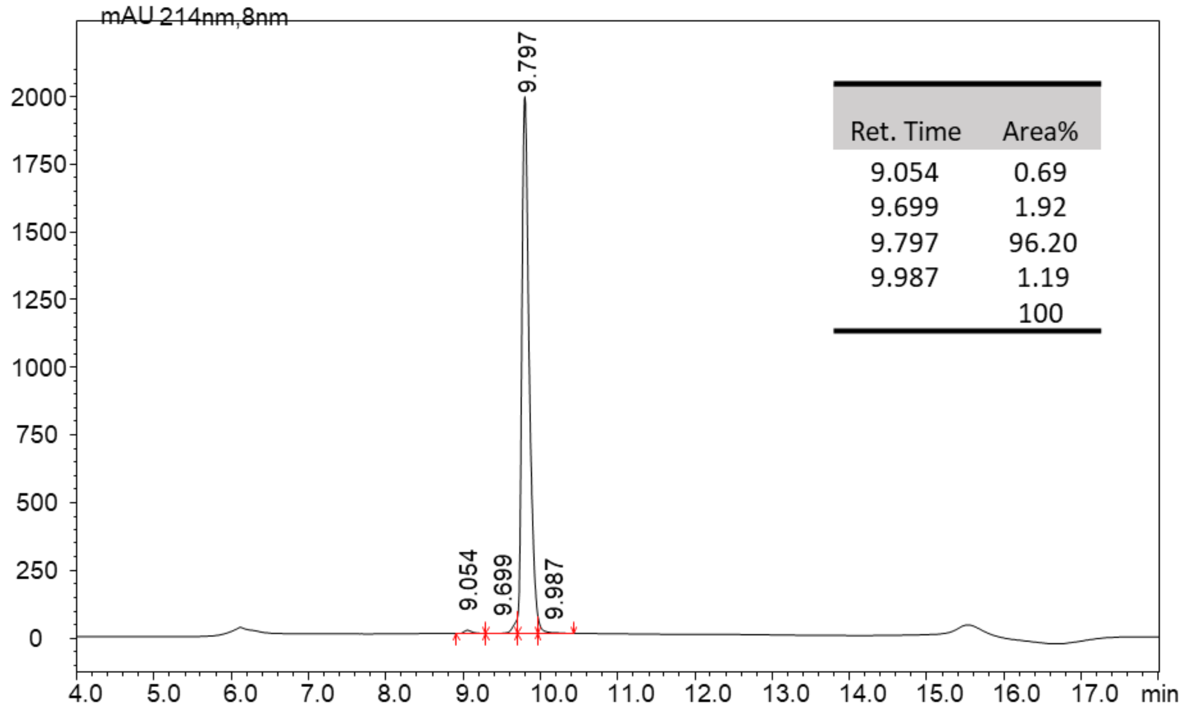

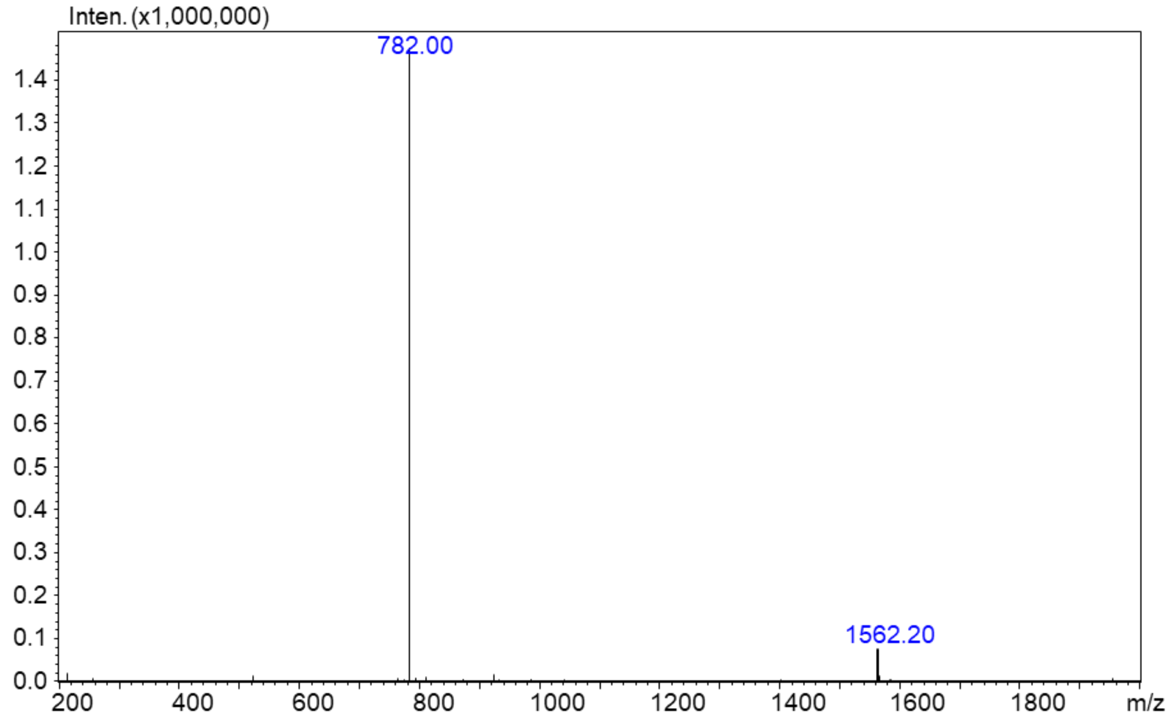

Figure S25. LC-MS Profile for peptide **31**. (A) LC profile at 214 nm. (B) MS spectrum of the peak at 9.79 min.

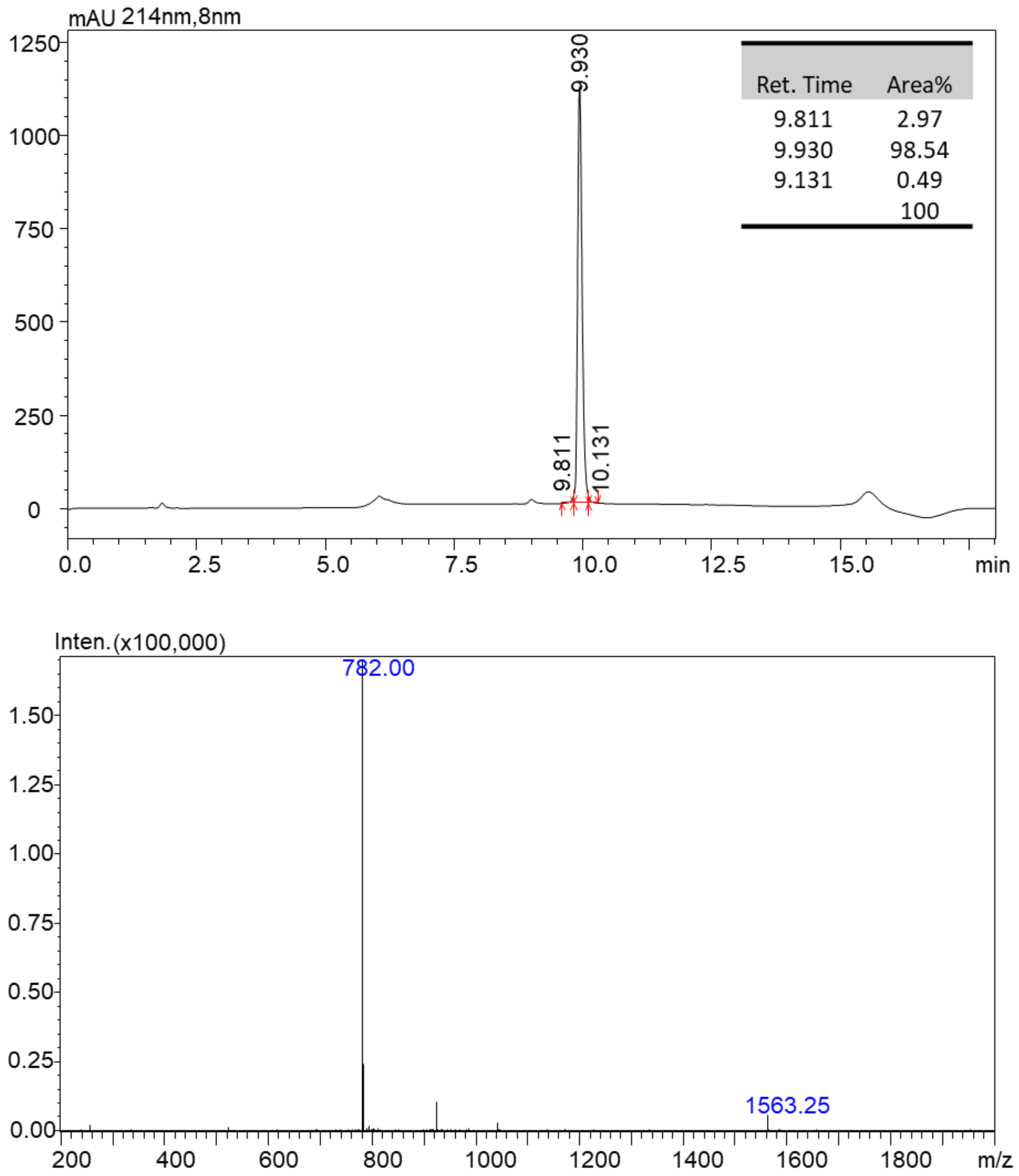

Figure S26. LC-MS Profile for peptide **32**. (A) LC profile at 214 nm. (B) MS spectrum of the peak at 9.93 min.

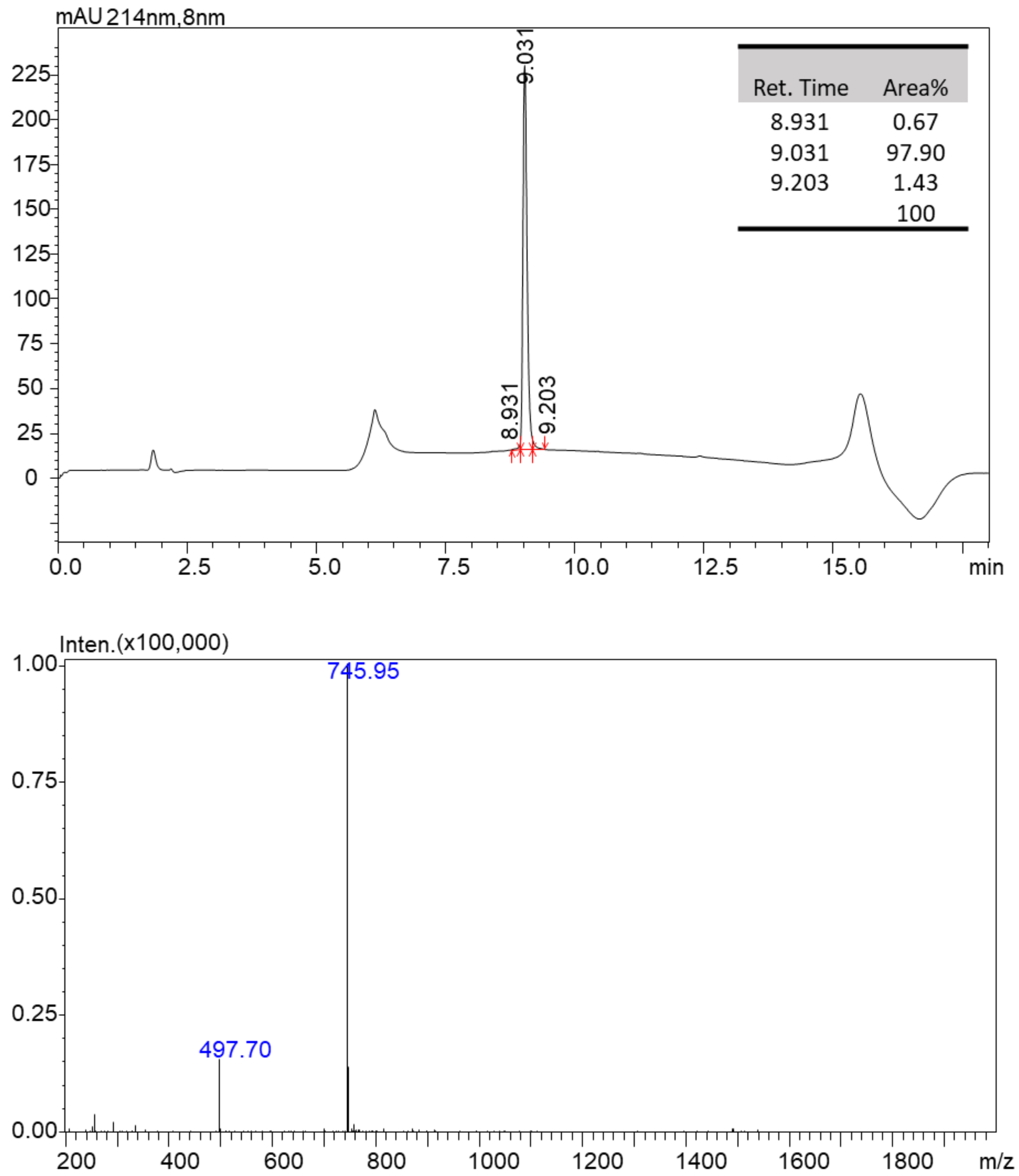

Figure S27. LC-MS Profile for peptide **33**. (A) LC profile at 214 nm. (B) MS spectrum of the peak at 9.03 min.

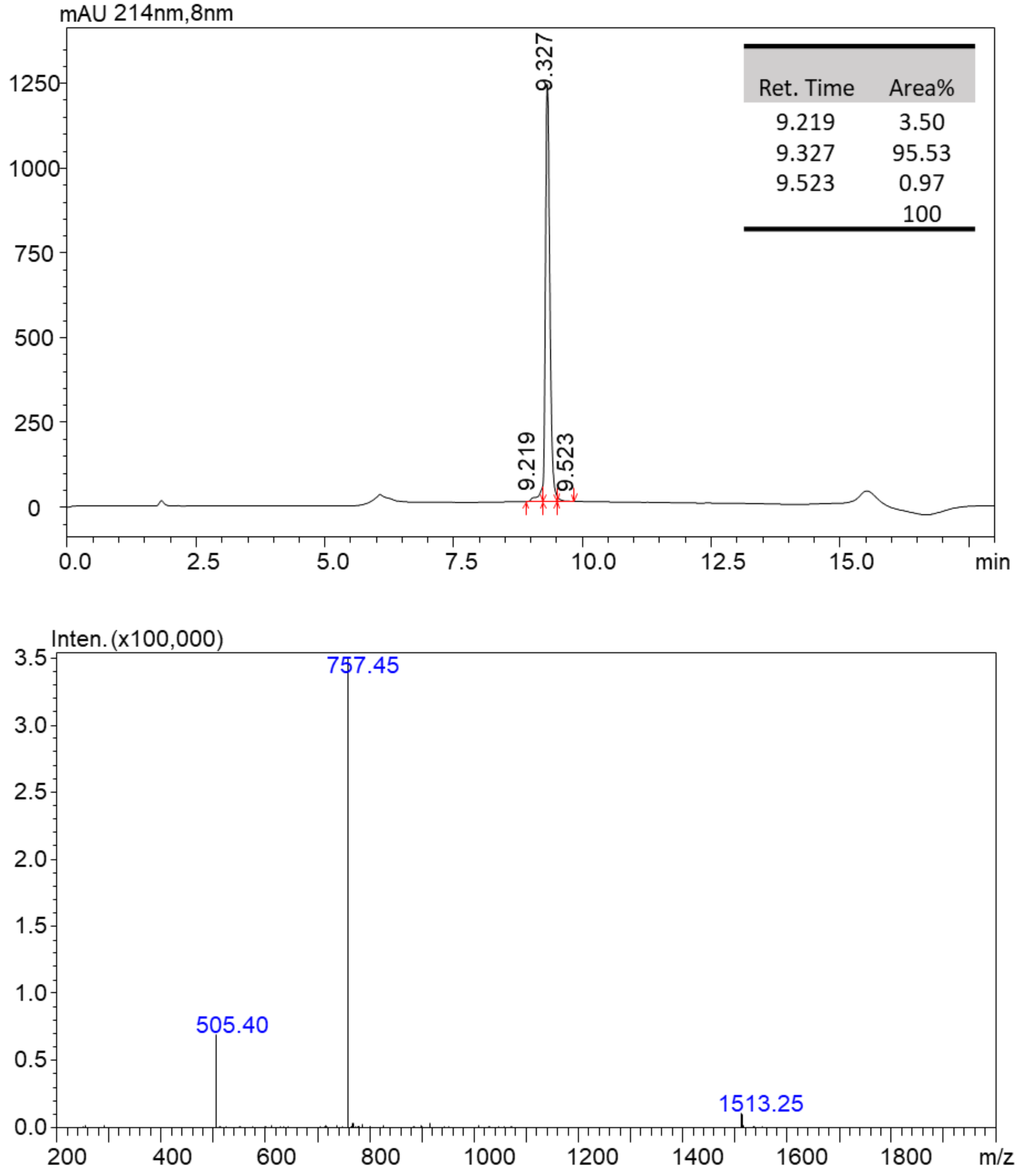

Figure S28. LC-MS Profile for peptide **34**. (A) LC profile at 214 nm. (B) MS spectrum of the peak at 9.33 min.

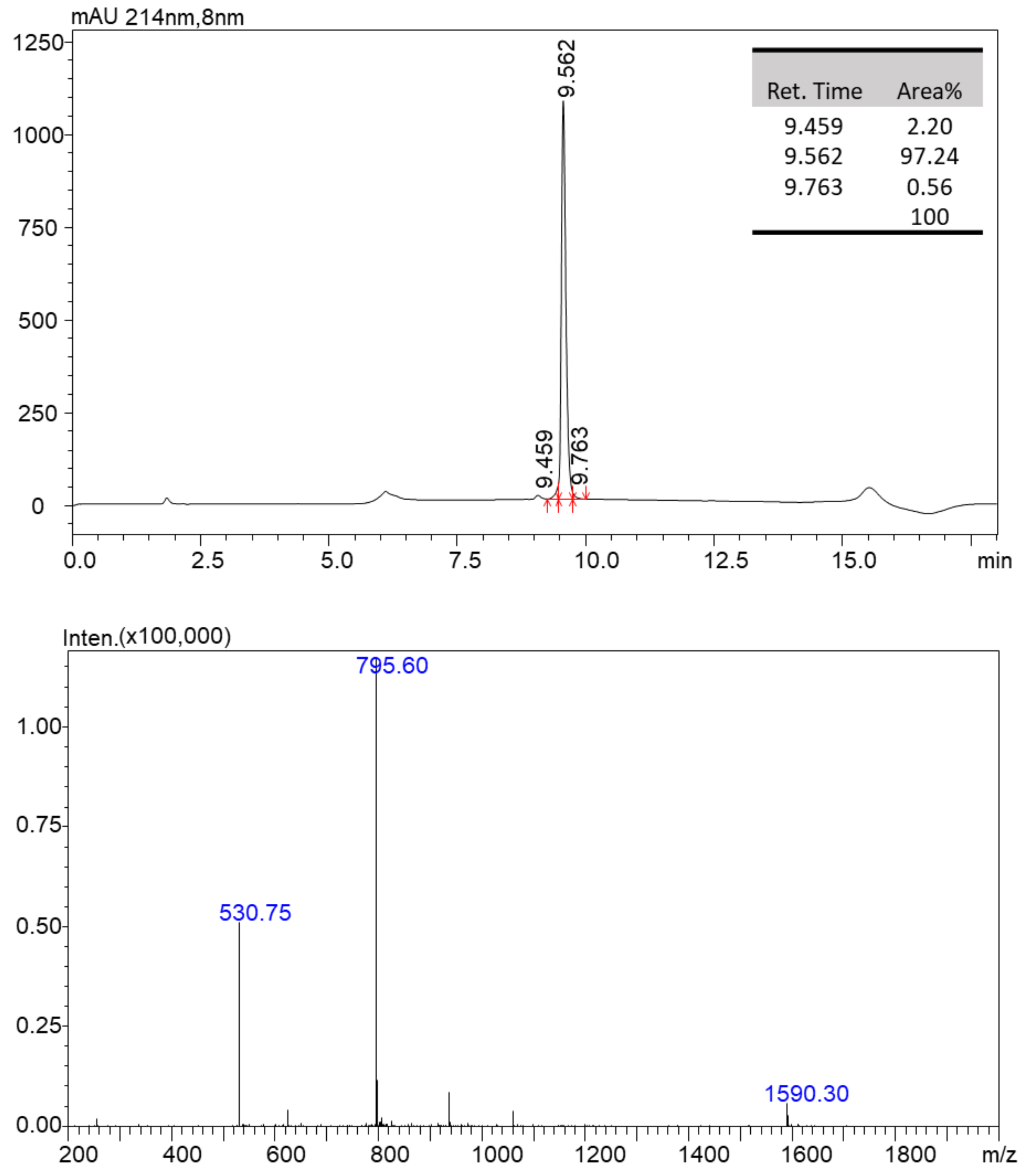

Figure S29. LC-MS Profile for peptide **35** (A) LC profile at 214 nm. (B) MS spectrum of the peak at 9.56 min.

Table S1: MICs against *P. aeruginoa* (Pa) PAO1 and PAO1R* strains

Peptide	Pa PAO1	Pa PAO1R
Poly B	0.5	16
Laterocidine (peptide **1**)	8	8
Peptide **11**	2	2

*The polymyxin-resistant paired strain of PAO1.

Table S2: MIC against *A. baumannii* 5075 strains

Peptide	*A. baumannii* 5075 S	*A. baumannii* 5075 R	*A. baumannii* 5075 D
**Laterocidine (peptide 1)**	8	>32	>32
**Peptide 11**	8	32	32

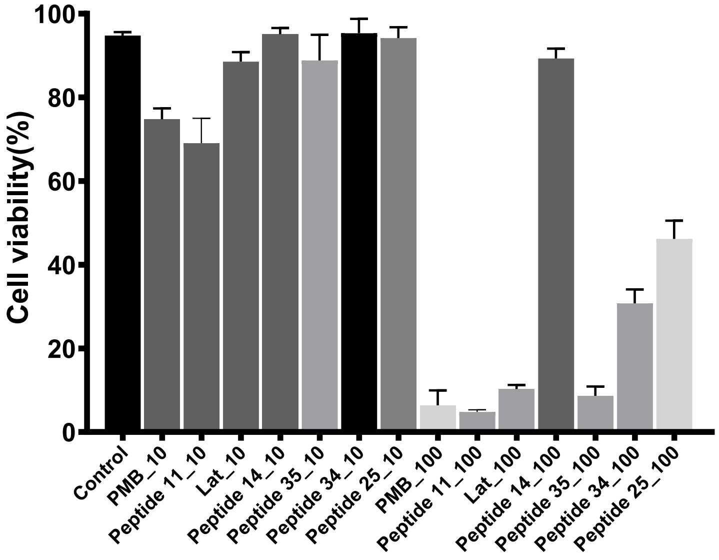

Figure S30. Cell viability of HK-2 cells after 24 h of treatment with polymyxin B (PMB) or laterocidine (Lat) and its analogues (Peptides **11**, **14**, **25**, **34**, and **35**) at concentrations of 0.01 mM and 0.1 mM (Mean ± SD; n = 3).

NMR Data:

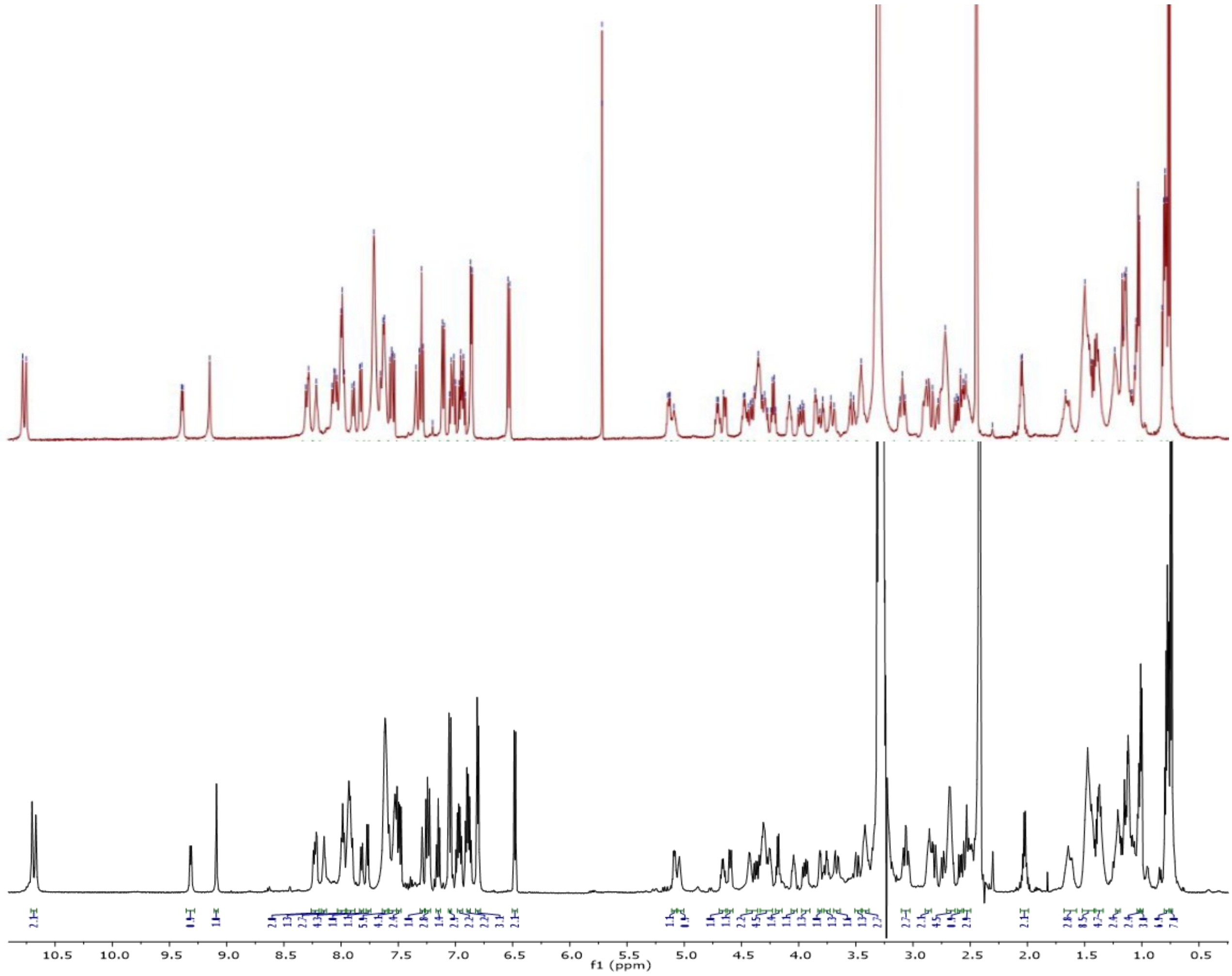

Figure S31. Overlay of literature laterocidine NMR(Black) and chemically synthesized laterocidine NMR (Brown) ^{1}H NMR (600 MHz, DMSO d6).

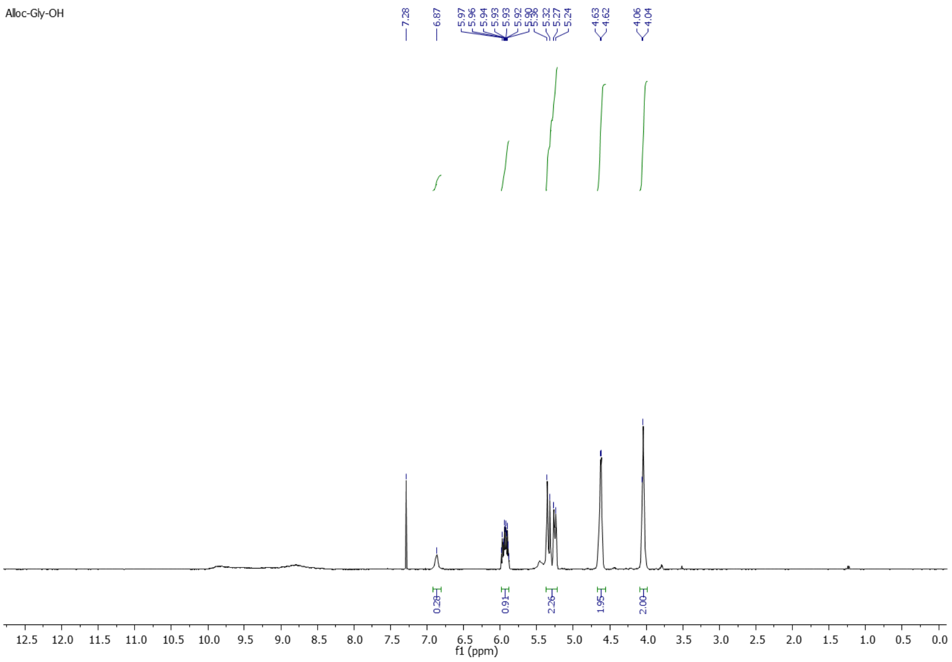

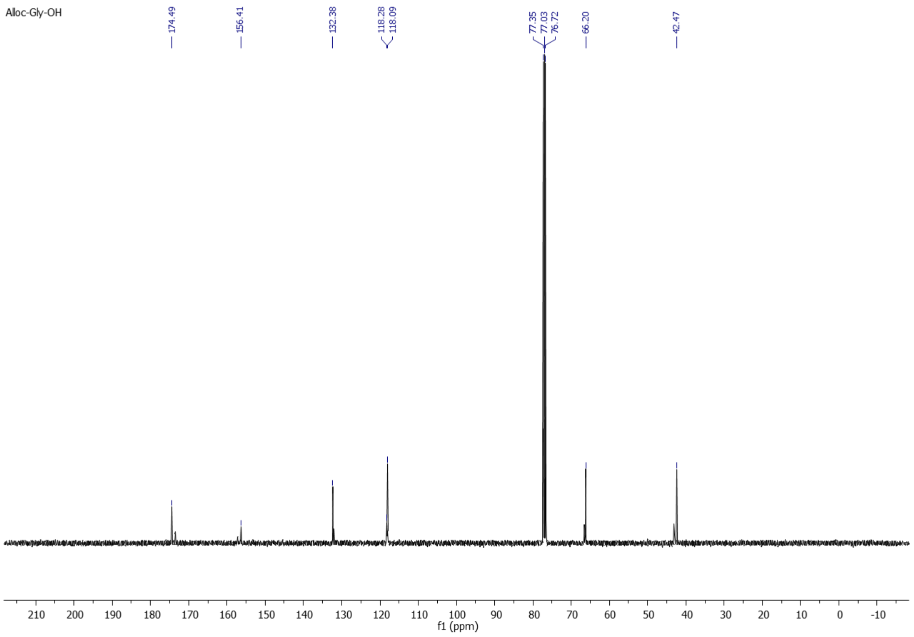

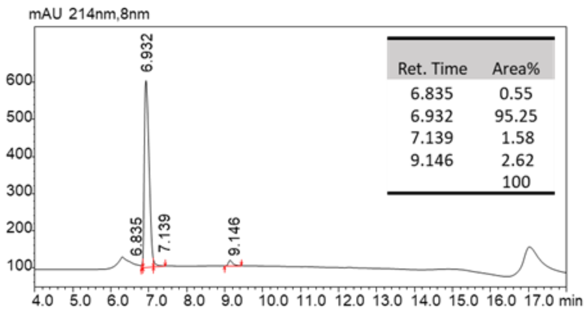

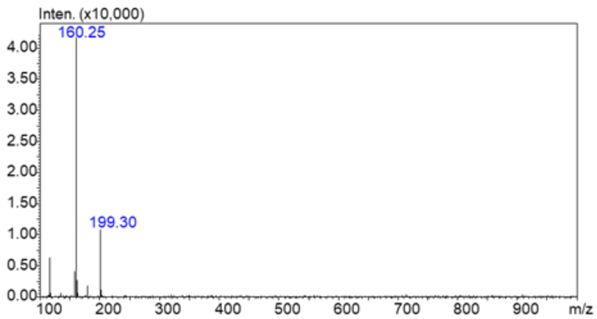

Figure S32. ^{1}H NMR, ^{13}C NMR and LC-MS of Alloc-Gly-OH

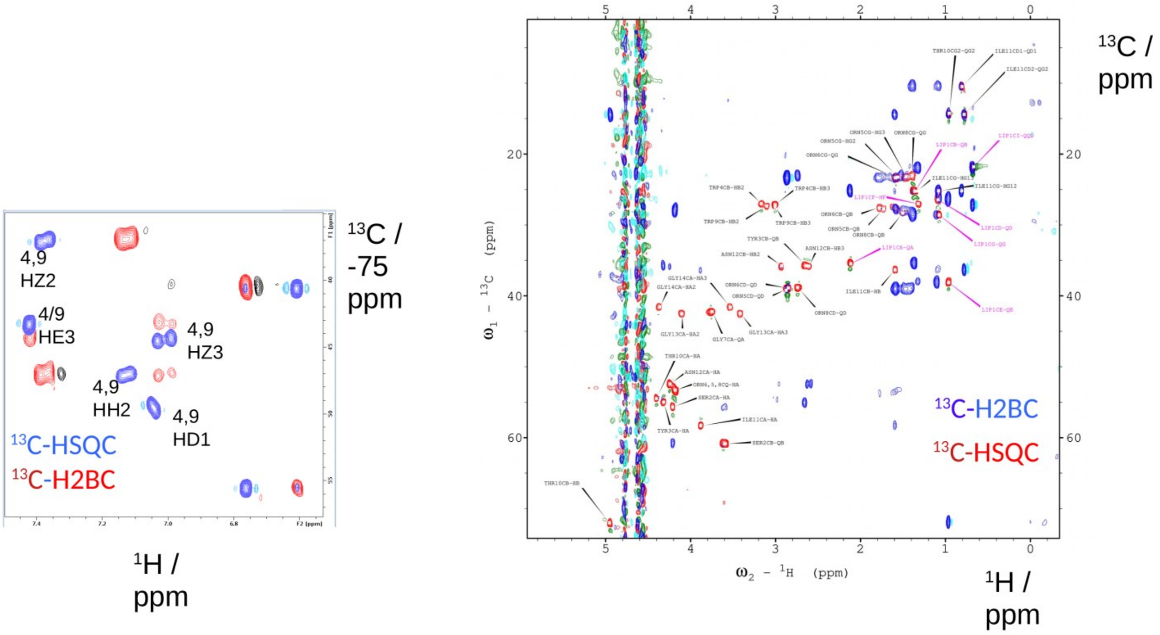

Figure S33. Superposition of 2D ^{13}C HSQC and 2D ^{13}C H2BC spectra of 1.5 mM laterocidine recorded at 23 °C in 50 mM acetate buffer (d_3), 10% D_2O, pH 4.5 showing the assignments of the aliphatic (right) and aromatic peaks (left). The aromatic region is folded by a 75 ppm ^{13}C sweep width requiring this to be added to the observed chemical shifts. Note that the numbering shown here starts with the lipid as residue 1, i.e., Lip1, D-Ser2, D-Tyr3, D-Trp4, Orn5, D-Orn6, Gly7, Orn8, Trp9, Thr10, Ile11, Asn12, Gly13, Gly14.

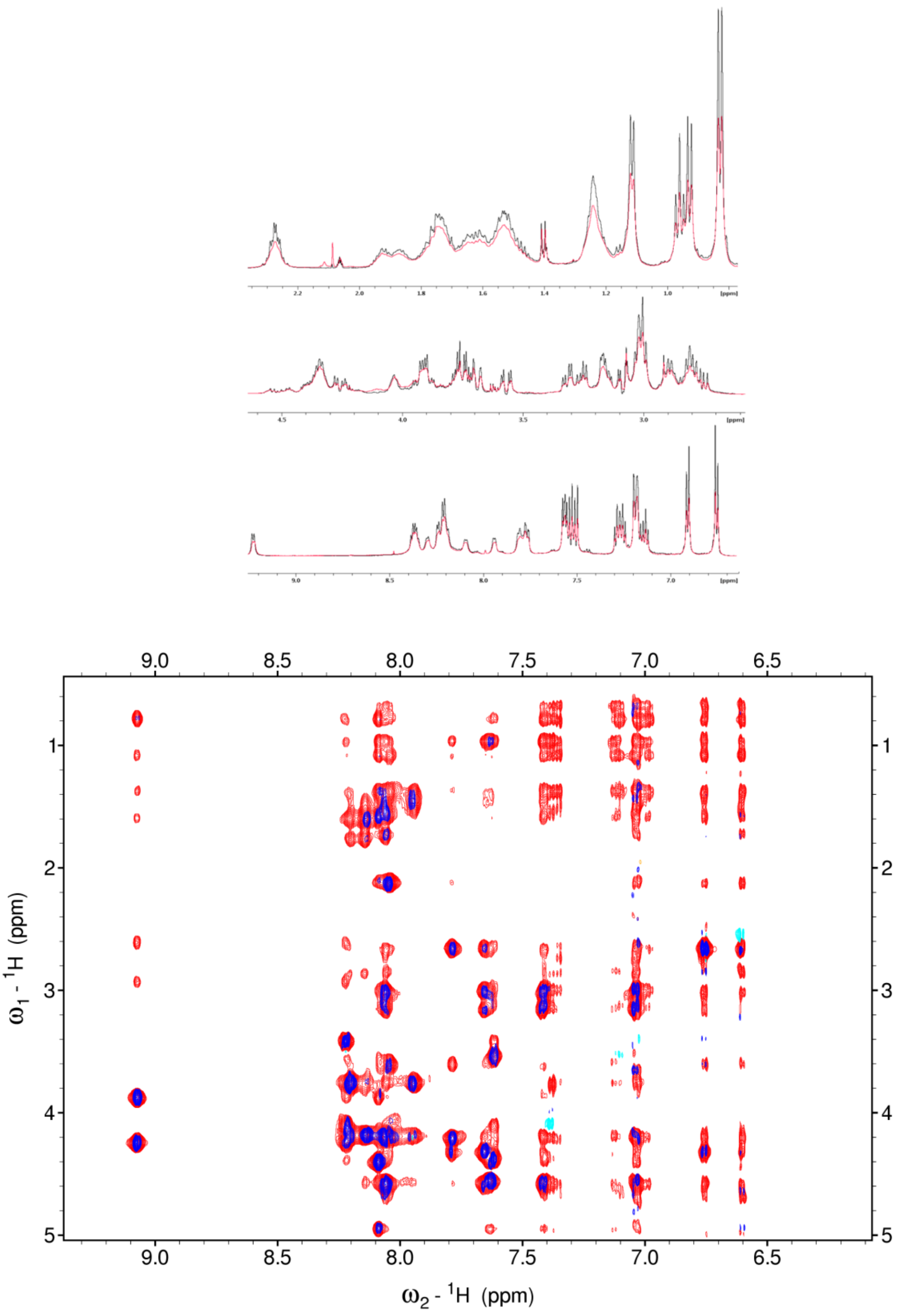

Figure S34. Top: Amide/aromatic region of the ^{1}H NMR spectrum during a titration of 1.5mM Nat (blue) after the addition of 16 µL (red) of 10 mg/mL *E. coli* O111:B4 LPS. Bottom: Superposition of 2D NOESY spectra of 1.5 mM laterocidine (red) and after addition of *E.coli* LPS O111:B4 (black), both recorded at 23 °C in 50 mM acetate buffer (d3), 10% D2O, pH 4.5. The 2D NOESY mixing times were 100 ms and the experiment times were 1.5 h.

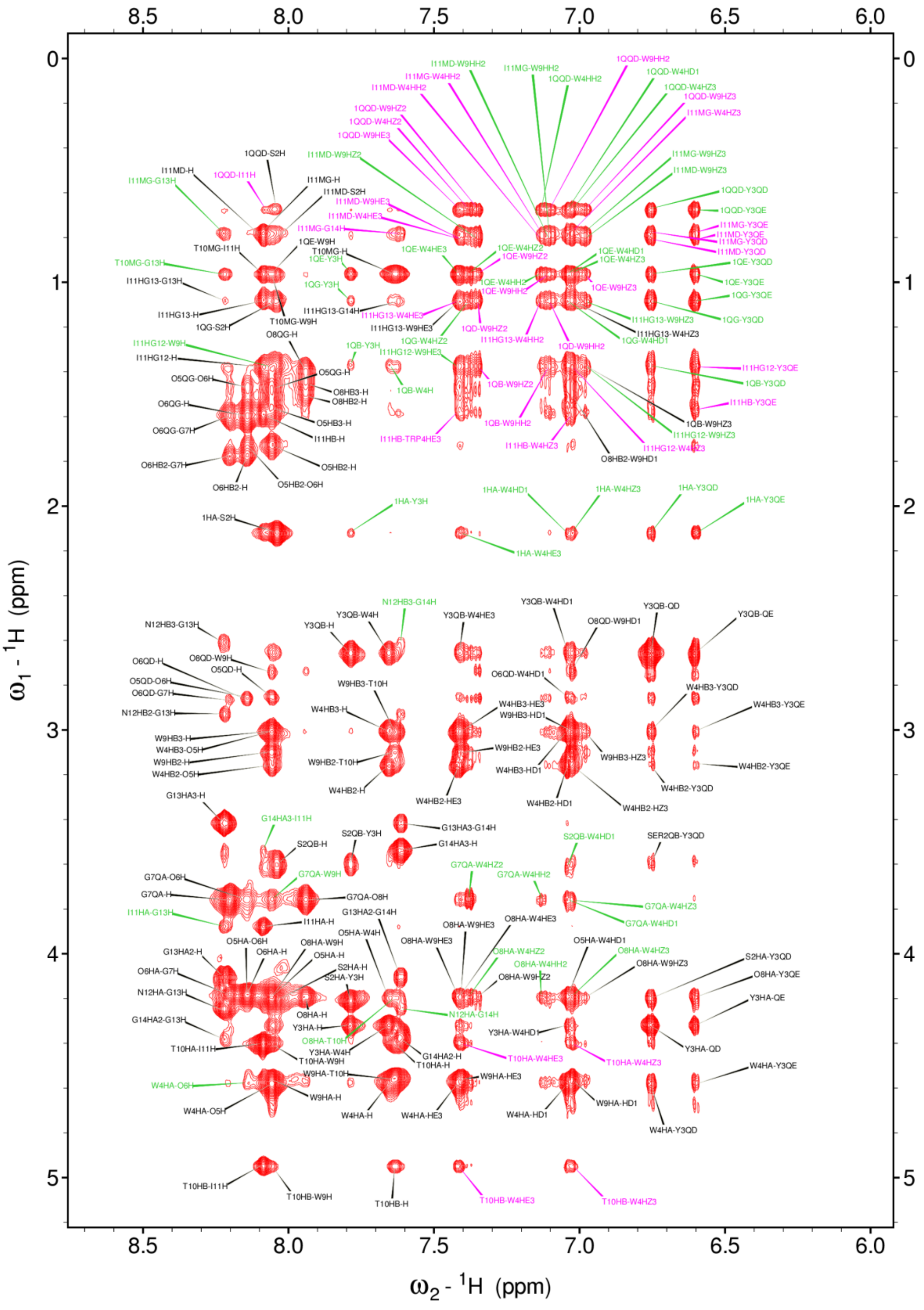

Figure S35. Part of the amide/aromatic-sidechain region of the 600 MHz ^{1}H 2D transferred ^{1}H-^{1}H NOESY NMR spectrum (140 ms mixing time) of a 500 μL sample of 1.5 mM laterocidine recorded at 25 °C in 50 mM acetate buffer (d_3), 10% D_2O, pH 4.5 after the addition of 16 μL of a 10 mg/mL *E. coli* O111:B4 LPS showing peak assignments. Those NOEs in green and magenta correspond to interesting medium or long range ^{1}H-^{1}H interactions in

laterocidine when bound to LPS. The experiment time was 4.5 h. Note that the numbering shown here starts with the lipid as residue 1, i.e., Lip1, D-Ser2, D-Tyr3, D-Trp4, Orn5, D-Orn6, Gly7, Orn8, Trp9, Thr10, Ile11, Asn12, Gly13, Gly14.

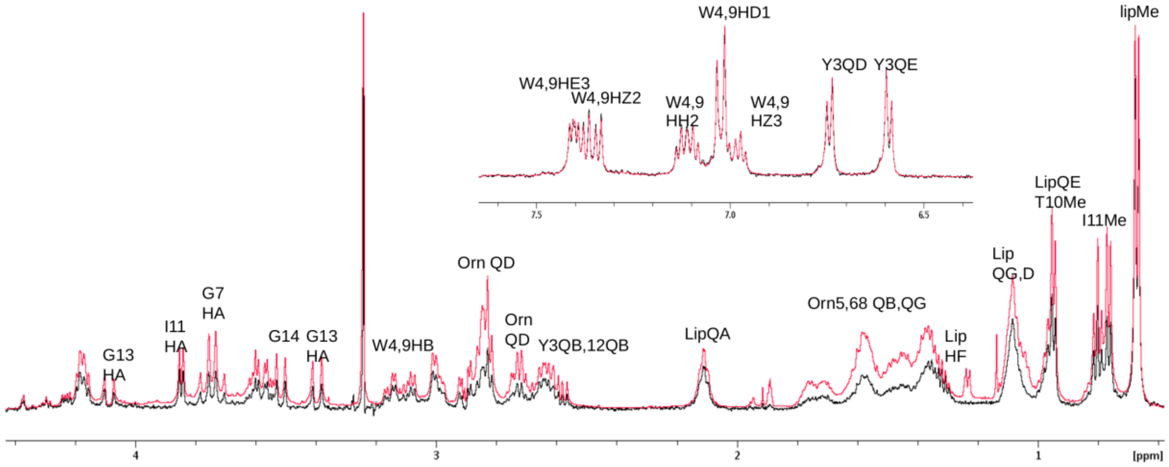

Figure S36. Overlay of the ‘on’ ^{1}H STD NMR experiment (red, after saturation at -4 ppm for 2.5s) with the ‘off’ reference (after saturation for 2.5s at 100 ppm) recorded on a sample of 0.75 mM laterocidine after the addition of 6 µL of 10 mg/mL *E.coli* O111:B4 LPS recorded in 50 mM Acetate(d3), pH 4.5, D_2O, 25 °C. The peak at 3.24 ppm is from a contaminant.

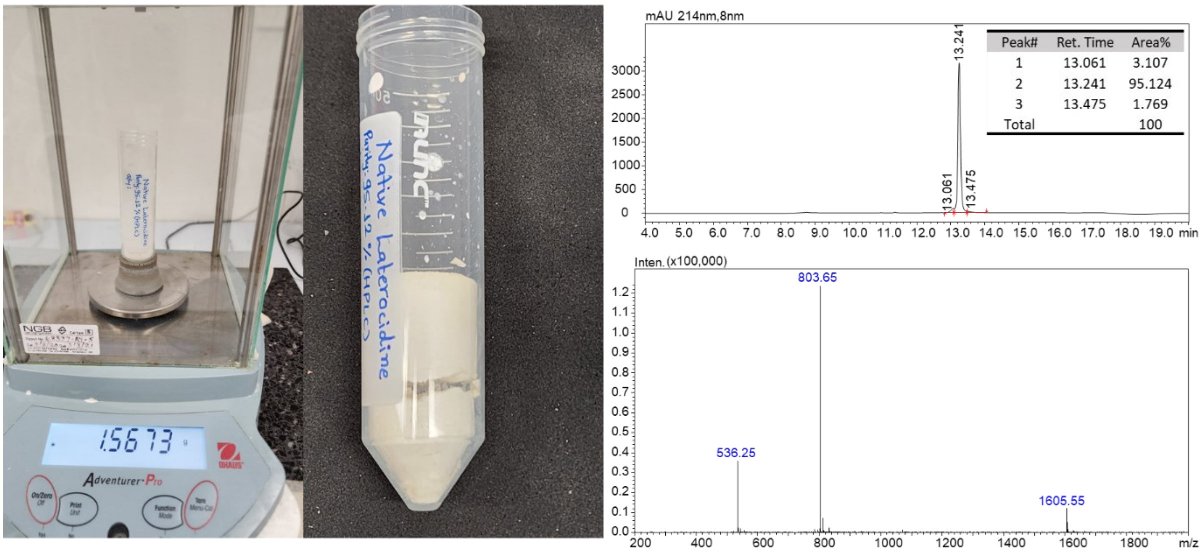

Figure S37. Gram-scale synthesis of native laterocidine: TFA salt was obtained in a yield of 1.6 g (9.9 %) from a 10 mmol scale.

Table S3: ^{1}H and ^{13}C chemical shift of laterocidine.

Res No	Res name	Atom	Shift / ppm
0	LIP	QG	1.084
0	LIP	CD	26.386
0	LIP	CE	38.115
0	LIP	QE	0.969
0	LIP	CF	27.096
0	LIP	HF	1.321
0	LIP	CI	21.844
0	LIP	QQD	0.678
0	LIP	CA	35.311
0	LIP	HA	2.117
0	LIP	CB	25.161
0	LIP	QB	1.364
0	LIP	CG	28.36
1	D-SER	H	8.041
1	D-SER	CA	55.497
1	D-SER	HA	4.209
1	D-SER	CB	60.828
1	D-SER	QB	3.603
2	D-TYR	H	7.788
2	D-TYR	CA	54.994
2	D-TYR	HA	4.32
2	D-TYR	CB	35.69

2	D-TYR	QB	2.656
2	D-TYR	QD	6.756
2	D-TYR	QE	6.601
2	D-TYR	CD1	130.329
2	D-TYR	CE1	115.455
3	D-TRP	H	7.656
3	D-TRP	HA	4.582
3	D-TRP	CB	27.007
3	D-TRP	HB2	3.16
3	D-TRP	HB3	3.006
3	D-TRP	CD1	124.334
3	D-TRP	CE3	117.927
3	D-TRP	HD1	7.044
3	D-TRP	HE3	7.419
3	D-TRP	CZ3	119.276
3	D-TRP	CZ2	111.783
3	D-TRP	HE1	9.993
3	D-TRP	HZ3	7.025
3	D-TRP	CH2	121.749
3	D-TRP	HZ2	7.382
3	D-TRP	HH2	7.134
4	D-ORN	CG	23.345
4	D-ORN	QG	1.467
4	D-ORN	CD	38.788

4	D-ORN	QD	2.853
4	D-ORN	H	8.058
4	D-ORN	HA	4.193
4	D-ORN	CB	27.807
4	D-ORN	HB3	1.556
4	D-ORN	HB2	1.728
5	ORN	H	8.143
5	ORN	CA	53.234
5	ORN	HA	4.183
5	ORN	CB	27.61
5	ORN	HB3	1.624
5	ORN	HB2	1.778
5	ORN	CG	23.424
5	ORN	QG	1.597
5	ORN	CD	38.993
5	ORN	QD	2.86
6	GLY	H	8.203
6	GLY	CA	42.12
6	GLY	QA	3.759
7	D-ORN	H	7.941
7	D-ORN	HA	4.193
7	D-ORN	CB	28.321
7	D-ORN	HB2	1.502
7	D-ORN	CG	23.029

7	D-ORN	HB3	1.452
7	D-ORN	QG	1.396
7	D-ORN	CD	38.857
7	D-ORN	QD	2.742
8	TRP	H	8.062
8	TRP	CA	53.486
8	TRP	HA	4.558
8	TRP	CB	27.333
8	TRP	HB2	3.091
8	TRP	HB3	3.011
8	TRP	CD1	124.334
8	TRP	CE3	118.265
8	TRP	HD1	7.029
8	TRP	HE3	7.411
8	TRP	CZ3	119.164
8	TRP	CZ2	111.67
8	TRP	HE1	9.979
8	TRP	HZ3	6.984
8	TRP	CH2	121.936
8	TRP	HZ2	7.353
8	TRP	HH2	7.106
9	THR	H	7.638
9	THR	CA	54.391
9	THR	HA	4.402

9	THR	CB	71.988
9	THR	HB	4.954
9	THR	QG2	0.964
9	THR	CG2	14.42
10	ILE	H	8.087
10	ILE	CA	58.213
10	ILE	HA	3.877
10	ILE	CB	36.298
10	ILE	HB	1.591
10	ILE	QG2	0.786
10	ILE	CG2	21.59
10	ILE	HG12	1.378
10	ILE	HG13	1.083
10	ILE	QD1	0.812
10	ILE	CD1	10.313
11	ASN	H	9.078
11	ASN	CA	52.379
11	ASN	HA	4.249
11	ASN	CB	35.682
11	ASN	HB2	2.931
11	ASN	HB3	2.611
11	ASN	HD21/22	Not observed
12	GLY	H	8.224
12	GLY	CA	42.421

12	GLY	HA2	4.104
12	GLY	HA3	3.415
13	GLY	H	7.618
13	GLY	CA	41.516
13	GLY	HA2	4.374
13	GLY	HA3	3.536

Table S4: NMR structural statistics:

<table>
<tr><td>NOEs:</td><td>376</td></tr>
<tr><td>short-range, |i-j|<=1:</td><td>195</td></tr>
<tr><td>medium-range, 1<|i-j|<5:</td><td>120</td></tr>
<tr><td>long-range, |i-j|>=5:</td><td>61</td></tr>
<tr><td>CYANA target function:</td><td>0.37 +/- 0.004 Å^2</td></tr>
<tr><td>RMS from experimental restraints[a]</td><td><41></td></tr>
<tr><td>Distances (Å)</td><td>0.017 ± 0.0024</td></tr>
<tr><td>Max NOE violation (Å)</td><td>0.13</td></tr>
<tr><td>Deviations from idealized covalent geometry</td><td></td></tr>
<tr><td>Bonds (Å)</td><td>0.0018 ± 0.00026</td></tr>
<tr><td>Angles (°)</td><td>0.3863 ± 0.0347</td></tr>
<tr><td>Impropers (°)</td><td>0.1831 ± 0.0272</td></tr>
<tr><td>Lennard-Jones potential energy[b]</td><td></td></tr>
<tr><td>E_{LJ} (kcal·mol^{-1})</td><td>-72.51 ± 1.52</td></tr>
<tr><td>RMSD to the mean structure (Å)</td><td></td></tr>
</table>

Backbone atoms	0.14 +/- 0.03 Å
All non-hydrogen atoms	0.47 +/- 0.13 Å

[a]<41> are the final 41 XplorNIH simulated annealing structures accepted from 100 calculated with no NOE violation > 0.13 Å. [b]The Lennard-Jones-van der Waas energy was not included in the target function for simulated annealing.

Table S5: Amide temperature coefficients (-ppb/K)

D-ser1	6.9
D-Tyr2	9.3
D-Trp3	6.3
D-Orn4	7.5
Orn5	9.2
Gly6	7.4
D-Orn7	2.5
Trp8	7.6
Thr9	0.7
Ile10	5.8
Asn11	6.3
Gly12	6.6
Gly13	0.9

Table S6: NOE Restraints

0	OCTN	HA	10	ILE	QD1	5.06
0	OCTN	HA	0	OCTN	HF	5.09
0	OCTN	HA	0	OCTN	QD	4.32
0	OCTN	HA	0	OCTN	QE	4.57
0	OCTN	HA	0	OCTN	QG	4.01
0	OCTN	HA	0	OCTN	QQD	4.73
0	OCTN	HA	1	DSER	H	3.62
0	OCTN	HA	1	DSER	HA	5.04
0	OCTN	HA	1	DSER	QB	5.11
0	OCTN	HA	2	DTYR	H	5.43
0	OCTN	HA	2	DTYR	QD	5.32
0	OCTN	HA	2	DTYR	QE	5.16
0	OCTN	HA	3	DTRP	H	6
0	OCTN	HA	3	DTRP	HD1	6
0	OCTN	HA	3	DTRP	HE3	6
0	OCTN	HA	3	DTRP	HZ3	6

0	OCTN	HA	5	SORN	QD	6
0	OCTN	HA	8	TRP	HH2	6
0	OCTN	QB	0	OCTN	QD	3.73
0	OCTN	QB	0	OCTN	QE	4.28
0	OCTN	QB	1	DSER	HA	6
0	OCTN	QB	1	DSER	QB	5.71
0	OCTN	QB	2	DTYR	H	5.54
0	OCTN	QB	2	DTYR	QB	6
0	OCTN	QB	2	DTYR	QD	5.2
0	OCTN	QB	3	DTRP	H	6
0	OCTN	QB	3	DTRP	HD1	5.8
0	OCTN	QB	3	DTRP	HE1	5.15
0	OCTN	QB	8	TRP	HH2	5.38
0	OCTN	QB	8	TRP	HZ2	5.97
0	OCTN	QD	0	OCTN	QQD	4.26
0	OCTN	QD	2	DTYR	QE	5.54
0	OCTN	QD	3	DTRP	HE1	5.91
0	OCTN	QD	3	DTRP	HH2	6
0	OCTN	QD	3	DTRP	HZ2	6
0	OCTN	QD	3	DTRP	HZ3	6
0	OCTN	QD	7	DORN	HA	5.76
0	OCTN	QD	8	TRP	HH2	4.95
0	OCTN	QD	8	TRP	HZ2	5.43
0	OCTN	QD	8	TRP	HZ3	5.37
0	OCTN	QE	10	ILE	HB	6
0	OCTN	QE	10	ILE	HG13	4.95
0	OCTN	QE	0	OCTN	QQD	3.61
0	OCTN	QE	1	DSER	H	6
0	OCTN	QE	1	DSER	HA	6

0	OCTN	QE	1	DSER	QB	6
0	OCTN	QE	2	DTYR	H	4.92
0	OCTN	QE	2	DTYR	QB	4.89
0	OCTN	QE	2	DTYR	QD	5.09
0	OCTN	QE	2	DTYR	QE	4.82
0	OCTN	QE	3	DTRP	HD1	5.8
0	OCTN	QE	3	DTRP	HE1	4.75
0	OCTN	QE	3	DTRP	HH2	6
0	OCTN	QE	3	DTRP	HZ2	4.97
0	OCTN	QE	3	DTRP	HZ3	4.61
0	OCTN	QE	7	DORN	HA	5.03
0	OCTN	QE	8	TRP	HE3	4.49
0	OCTN	QE	8	TRP	HH2	6
0	OCTN	QE	8	TRP	HZ2	5.39
0	OCTN	QE	8	TRP	HZ3	5.11
0	OCTN	QG	0	OCTN	HF	4.17
0	OCTN	QG	0	OCTN	QE	3.11
0	OCTN	QG	0	OCTN	QQD	3.69
0	OCTN	QG	1	DSER	H	4.67
0	OCTN	QG	1	DSER	HA	5.01
0	OCTN	QG	1	DSER	QB	5.43
0	OCTN	QG	2	DTYR	H	6
0	OCTN	QG	2	DTYR	QB	5.1
0	OCTN	QG	2	DTYR	QD	4.94
0	OCTN	QG	2	DTYR	QE	5.19
0	OCTN	QG	3	DTRP	H	6
0	OCTN	QG	3	DTRP	HD1	6
0	OCTN	QG	3	DTRP	HE1	5.6
0	OCTN	QQD	10	ILE	HA	6

0	OCTN	QQD	10	ILE	HB	5.11
0	OCTN	QQD	1	DSER	H	5.23
0	OCTN	QQD	1	DSER	HA	6
0	OCTN	QQD	1	DSER	QB	5.71
0	OCTN	QQD	2	DTYR	QB	4.85
0	OCTN	QQD	2	DTYR	QD	4.84
0	OCTN	QQD	2	DTYR	QE	4.82
0	OCTN	QQD	3	DTRP	HB3	5.85
0	OCTN	QQD	3	DTRP	HD1	6
0	OCTN	QQD	3	DTRP	HE1	5.62
0	OCTN	QQD	3	DTRP	HH2	6
0	OCTN	QQD	3	DTRP	HZ2	6
0	OCTN	QQD	3	DTRP	HZ3	5.11
0	OCTN	QQD	7	DORN	HA	6
0	OCTN	QQD	8	TRP	HB2	6
0	OCTN	QQD	8	TRP	HE3	4.54
0	OCTN	QQD	8	TRP	HH2	6
0	OCTN	QQD	8	TRP	HZ2	5.79
0	OCTN	QQD	8	TRP	HZ3	4.93
1	DSER	H	2	DTYR	H	4.73
1	DSER	H	2	DTYR	QB	5.37
1	DSER	H	2	DTYR	QD	6
1	DSER	HA	2	DTYR	QB	5.05
1	DSER	HA	2	DTYR	QD	5.11
1	DSER	HA	3	DTRP	HD1	4.31
1	DSER	QB	2	DTYR	H	4.86
1	DSER	QB	2	DTYR	QB	5.93
1	DSER	QB	3	DTRP	HD1	5.85
2	DTYR	H	2	DTYR	QB	4.04

2	DTYR	H	2	DTYR	QD	4.64
2	DTYR	H	2	DTYR	QE	4.95
2	DTYR	H	3	DTRP	H	4.32
2	DTYR	H	3	DTRP	HD1	6
2	DTYR	H	3	DTRP	HE3	6
2	DTYR	HA	2	DTYR	QD	4.29
2	DTYR	HA	2	DTYR	QE	5.62
2	DTYR	HA	3	DTRP	HB3	5.42
2	DTYR	HA	3	DTRP	HD1	5.19
2	DTYR	QB	3	DTRP	H	4.38
2	DTYR	QB	3	DTRP	HA	5.5
2	DTYR	QB	3	DTRP	HD1	5.7
2	DTYR	QB	3	DTRP	HE1	6
2	DTYR	QB	3	DTRP	HE3	6
2	DTYR	QD	10	ILE	HG13	6
2	DTYR	QD	10	ILE	QD1	5.39
2	DTYR	QD	10	ILE	QG2	5.95
2	DTYR	QD	3	DTRP	H	4.83
2	DTYR	QD	3	DTRP	HA	5.28
2	DTYR	QD	3	DTRP	HB2	6
2	DTYR	QD	3	DTRP	HB3	5.27
2	DTYR	QD	3	DTRP	HD1	6
2	DTYR	QD	3	DTRP	HE1	5.27
2	DTYR	QD	3	DTRP	HE3	4.87
2	DTYR	QD	3	DTRP	HH2	6
2	DTYR	QD	3	DTRP	HZ2	6
2	DTYR	QD	3	DTRP	HZ3	6
2	DTYR	QE	10	ILE	HB	6
2	DTYR	QE	10	ILE	HG13	5.15

2	DTYR	QE	10	ILE	QD1	5.44
2	DTYR	QE	10	ILE	QG2	5.09
2	DTYR	QE	3	DTRP	H	5.45
2	DTYR	QE	3	DTRP	HA	5.69
2	DTYR	QE	3	DTRP	HB2	5.79
2	DTYR	QE	3	DTRP	HB3	5.4
2	DTYR	QE	3	DTRP	HD1	6
2	DTYR	QE	3	DTRP	HE1	5.29
2	DTYR	QE	3	DTRP	HE3	4.85
2	DTYR	QE	3	DTRP	HZ2	6
2	DTYR	QE	3	DTRP	HZ3	4.68
2	DTYR	QE	7	DORN	HA	5.18
2	DTYR	QE	8	TRP	H	5.41
3	DTRP	H	3	DTRP	HB3	4.16
3	DTRP	H	3	DTRP	HE1	5.35
3	DTRP	H	4	DORN	H	4.38
3	DTRP	H	4	DORN	HA	5.27
3	DTRP	HA	3	DTRP	HD1	3.84
3	DTRP	HA	3	DTRP	HE3	4.99
3	DTRP	HA	5	SORN	H	6
3	DTRP	HB2	3	DTRP	HD1	3.85
3	DTRP	HB2	3	DTRP	HE1	5.25
3	DTRP	HB2	3	DTRP	HZ3	5.63
3	DTRP	HB2	4	DORN	H	4.69
3	DTRP	HB2	4	DORN	HA	6
3	DTRP	HB3	4	DORN	H	4.32
3	DTRP	HB3	4	DORN	HA	6
3	DTRP	HD1	4	DORN	H	4.27
3	DTRP	HD1	4	DORN	HA	5.01

3	DTRP	HD1	4	DORN	QG	6
3	DTRP	HD1	5	SORN	HA	5.31
3	DTRP	HD1	5	SORN	QD	5.25
3	DTRP	HD1	6	GLY	H	5.46
3	DTRP	HD1	6	GLY	QA	5.29
3	DTRP	HE1	5	SORN	HA	5.66
3	DTRP	HE1	6	GLY	H	5.21
3	DTRP	HE1	6	GLY	QA	4.89
3	DTRP	HE1	7	DORN	HA	6
3	DTRP	HE3	9	THR	H	4.84
3	DTRP	HE3	9	THR	HA	4.91
3	DTRP	HE3	9	THR	HB	6
3	DTRP	HE3	10	ILE	HB	6
3	DTRP	HE3	4	DORN	H	5.47
3	DTRP	HE3	4	DORN	QG	5.66
3	DTRP	HE3	6	GLY	QA	5.57
3	DTRP	HE3	7	DORN	HA	6
3	DTRP	HH2	10	ILE	HG12	6
3	DTRP	HH2	10	ILE	HG13	6
3	DTRP	HH2	10	ILE	QD1	6
3	DTRP	HH2	10	ILE	QG2	6
3	DTRP	HH2	6	GLY	QA	5.24
3	DTRP	HH2	7	DORN	HA	5.13
3	DTRP	HH2	8	TRP	H	5.66
3	DTRP	HH2	8	TRP	HE3	4.01
3	DTRP	HZ2	10	ILE	HG13	5.61
3	DTRP	HZ2	10	ILE	QD1	6
3	DTRP	HZ2	5	SORN	QD	6
3	DTRP	HZ2	6	GLY	H	6

3	DTRP	HZ2	6	GLY	QA	5.22
3	DTRP	HZ2	7	DORN	HA	5.39
3	DTRP	HZ3	9	THR	H	4.82
3	DTRP	HZ3	9	THR	HA	6
3	DTRP	HZ3	9	THR	HB	5.25
3	DTRP	HZ3	9	THR	QG2	6
3	DTRP	HZ3	10	ILE	HB	6
3	DTRP	HZ3	10	ILE	HG12	4.59
3	DTRP	HZ3	10	ILE	HG13	4.4
3	DTRP	HZ3	10	ILE	QD1	4.87
3	DTRP	HZ3	10	ILE	QG2	5.13
3	DTRP	HZ3	6	GLY	H	5.54
3	DTRP	HZ3	6	GLY	QA	4.88
3	DTRP	HZ3	7	DORN	H	5.63
3	DTRP	HZ3	7	DORN	HA	4.28
3	DTRP	HZ3	8	TRP	H	4.3
4	DORN	H	4	DORN	QD	5.02
4	DORN	H	4	DORN	QG	4.22
4	DORN	H	6	GLY	H	6
4	DORN	HA	4	DORN	QD	4.84
4	DORN	HB2	4	DORN	QD	4.21
4	DORN	HB2	5	SORN	H	5.05
4	DORN	HB2	5	SORN	HA	4.5
4	DORN	HB3	4	DORN	QD	3.63
4	DORN	QD	5	SORN	H	5.16
4	DORN	QG	5	SORN	H	5.15
4	DORN	QG	5	SORN	HA	6
5	SORN	H	5	SORN	QD	6
5	SORN	H	5	SORN	QG	4.32

5	SORN	H	6	GLY	QA	5.06
5	SORN	HA	5	SORN	QD	4.25
5	SORN	HA	5	SORN	QG	3.7
5	SORN	HA	6	GLY	QA	5.01
5	SORN	QB	6	GLY	QA	5.81
5	SORN	QD	6	GLY	H	5.39
5	SORN	QD	6	GLY	QA	6
5	SORN	QG	6	GLY	H	4.56
5	SORN	QG	6	GLY	QA	5.15
6	GLY	H	7	DORN	H	4.49
6	GLY	QA	9	THR	QG2	6
6	GLY	QA	7	DORN	HA	5.26
6	GLY	QA	7	DORN	QG	6
6	GLY	QA	8	TRP	H	4.94
7	DORN	H	9	THR	H	6
7	DORN	H	9	THR	QG2	5.75
7	DORN	H	7	DORN	QD	5.6
7	DORN	H	7	DORN	QG	4.47
7	DORN	H	8	TRP	H	4.17
7	DORN	H	8	TRP	HD1	5.81
7	DORN	HA	9	THR	H	5.07
7	DORN	HA	10	ILE	HG13	6
7	DORN	HA	7	DORN	QD	4.94
7	DORN	HA	7	DORN	QG	4.07
7	DORN	HA	8	TRP	H	3.47
7	DORN	HA	8	TRP	HB2	6
7	DORN	HA	8	TRP	HD1	6
7	DORN	HA	8	TRP	HE1	5.15
7	DORN	HA	8	TRP	HE3	5.1

7	DORN	HA	8	TRP	HH2	6
7	DORN	HA	8	TRP	HZ2	5.79
7	DORN	HA	8	TRP	HZ3	6
7	DORN	QB	8	TRP	H	4.25
7	DORN	QB	8	TRP	HD1	4.92
7	DORN	QB	8	TRP	HE3	5.81
7	DORN	QD	8	TRP	H	5.31
7	DORN	QD	8	TRP	HD1	6
7	DORN	QD	8	TRP	HE1	5.35
7	DORN	QD	8	TRP	HZ2	6
7	DORN	QG	8	TRP	H	4.49
8	TRP	H	9	THR	H	4.56
8	TRP	H	9	THR	HA	4.68
8	TRP	H	9	THR	HB	6
8	TRP	H	9	THR	QG2	4.71
8	TRP	H	10	ILE	HG13	4.44
8	TRP	H	8	TRP	HE1	5.47
8	TRP	H	8	TRP	HE3	4.4
8	TRP	HA	9	THR	QG2	4.4
8	TRP	HA	8	TRP	HD1	4
8	TRP	HA	8	TRP	HE1	5.6
8	TRP	HA	8	TRP	HE3	5.03
8	TRP	HB2	9	THR	H	4.48
8	TRP	HB2	9	THR	QG2	4.52
8	TRP	HB2	10	ILE	HG12	5.18
8	TRP	HB2	10	ILE	HG13	5.08
8	TRP	HB2	10	ILE	QD1	6
8	TRP	HB2	10	ILE	QG2	5.24
8	TRP	HB2	8	TRP	HZ3	5.23

8	TRP	HB3	9	THR	H	4.75
8	TRP	HB3	9	THR	QG2	5.11
8	TRP	HB3	10	ILE	HG13	5.85
8	TRP	HE3	9	THR	H	6
8	TRP	HE3	10	ILE	H	5.08
8	TRP	HE3	10	ILE	HB	6
8	TRP	HE3	10	ILE	HG12	4.68
8	TRP	HE3	10	ILE	HG13	4.78
8	TRP	HE3	10	ILE	QD1	6
8	TRP	HE3	10	ILE	QG2	4.92
8	TRP	HH2	10	ILE	QD1	5.4
8	TRP	HH2	10	ILE	QG2	6
8	TRP	HZ2	10	ILE	QD1	6
8	TRP	HZ3	10	ILE	HG12	5.67
8	TRP	HZ3	10	ILE	HG13	5
8	TRP	HZ3	10	ILE	QD1	5.13
8	TRP	HZ3	10	ILE	QG2	4.79
9	THR	H	9	THR	QG2	3.83
9	THR	H	10	ILE	HG12	6
9	THR	H	10	ILE	HG13	5.68
9	THR	HA	9	THR	QG2	3.8
9	THR	HA	10	ILE	H	3.42
9	THR	HA	10	ILE	HA	5.43
9	THR	HA	10	ILE	HB	5.37
9	THR	HA	10	ILE	QD1	5.51
9	THR	HB	10	ILE	H	4.62
9	THR	QG2	10	ILE	H	4.32
9	THR	QG2	10	ILE	HA	4.86
9	THR	QG2	11	ASN	H	6

9	THR	QG2	12	GLY	H	4.94
9	THR	QG2	13	GLY	QA	5.45
10	ILE	H	10	ILE	HB	3.95
10	ILE	H	10	ILE	HG12	4.6
10	ILE	H	10	ILE	HG13	4.45
10	ILE	H	10	ILE	QD1	5.07
10	ILE	H	10	ILE	QG2	4.36
10	ILE	H	11	ASN	H	5.36
10	ILE	H	13	GLY	H	4.65
10	ILE	H	13	GLY	QA	5.41
10	ILE	HA	10	ILE	QD1	4.42
10	ILE	HA	10	ILE	QG2	4.08
10	ILE	HA	11	ASN	HA	5.25
10	ILE	HA	12	GLY	H	5.12
10	ILE	HB	10	ILE	QD1	4.08
10	ILE	HB	11	ASN	H	5.04
10	ILE	HG12	11	ASN	H	5.16
10	ILE	HG12	12	GLY	H	6
10	ILE	HG12	13	GLY	H	6
10	ILE	HG13	11	ASN	H	5.13
10	ILE	HG13	11	ASN	QB	5.81
10	ILE	HG13	13	GLY	H	5.58
10	ILE	QD1	11	ASN	H	6
10	ILE	QG2	10	ILE	HG13	3.9
10	ILE	QG2	11	ASN	H	4.3
10	ILE	QG2	11	ASN	HA	4.63
10	ILE	QG2	11	ASN	HB2	4.79
10	ILE	QG2	11	ASN	HB3	4.79
10	ILE	QG2	11	ASN	QB	4.1

10	ILE	QG2	12	GLY	H	4.91
10	ILE	QG2	13	GLY	H	4.97
11	ASN	H	12	GLY	H	4.46
11	ASN	H	13	GLY	H	5.57
11	ASN	HA	13	GLY	H	5.34
11	ASN	HB2	12	GLY	H	5.07
11	ASN	HB2	13	GLY	H	5.64
11	ASN	HB3	12	GLY	H	5.07
11	ASN	HB3	13	GLY	H	5.64
12	GLY	H	13	GLY	H	4.23
12	GLY	H	13	GLY	HA2	5.47
12	GLY	H	13	GLY	HA3	5.47
Ether bond restraints						
9	THR	OG1	13	GLY	C	1.47
9	THR	OG1	13	GLY	CA	2.55
9	THR	OG1	13	GLY	O	2.36

References:

1 Roberts, K. D. *et al.* Antimicrobial Activity and Toxicity of the Major Lipopeptide Components of Polymyxin B and Colistin: Last-Line Antibiotics against Multidrug-Resistant Gram-Negative Bacteria. *ACS Infectious Diseases* **1**, 568-575, doi:10.1021/acsinfecdis.5b00085 (2015).

2 Institute, C. a. L. S. in *M100-S23* (Clinical and Laboratory Standards Institute, Wayne, PA, USA, 2013).

3 Zoete, V., Cuendet, M. A., Grosdidier, A. & Michielin, O. SwissParam: A fast force field generation tool for small organic molecules. *Journal of Computational Chemistry* **32**, 2359-2368, doi:https://doi.org/10.1002/jcc.21816 (2011).

4 Van Der Spoel, D. *et al.* GROMACS: Fast, flexible, and free. *Journal of Computational Chemistry* **26**, 1701-1718, doi:https://doi.org/10.1002/jcc.20291 (2005).

5 Klauda, J. B. *et al.* Update of the CHARMM All-Atom Additive Force Field for Lipids: Validation on Six Lipid Types. *The Journal of Physical Chemistry B* **114**, 7830-7843, doi:10.1021/jp101759q (2010).

6 Parrinello, M. & Rahman, A. Polymorphic transitions in single crystals: A new molecular dynamics method. *Journal of Applied Physics* **52**, 7182-7190, doi:10.1063/1.328693 (1981).

7 Nosé, S. A molecular dynamics method for simulations in the canonical ensemble. *Molecular Physics* **52**, 255-268, doi:10.1080/00268978400101201 (1984).

8 Hoover, W. G. Canonical dynamics: Equilibrium phase-space distributions. *Physical Review A* **31**, 1695-1697, doi:10.1103/PhysRevA.31.1695 (1985).

9 Darden, T., York, D. & Pedersen, L. Particle mesh Ewald: An N·log(N) method for Ewald sums in large systems. *The Journal of Chemical Physics* **98**, 10089-10092, doi:10.1063/1.464397 (1993).
